# Synthesis and SAR of Tetracyclic Inhibitors of Protein Kinase CK2 Derived from Furocarbazole W16

**DOI:** 10.1002/cmdc.202000040

**Published:** 2020-04-27

**Authors:** Lukas Kröger, Constantin G. Daniliuc, Deeba Ensan, Sebastian Borgert, Christian Nienberg, Miriam Lauwers, Michaela Steinkrüger, Joachim Jose, Markus Pietsch, Bernhard Wünsch

**Affiliations:** ^1^ Institut für Pharmazeutische und Medizinische Chemie Westfälische Wilhelms-Universität Münster Corrensstraße 48 8149 Münster Germany; ^2^ Organisch-Chemisches Institut Westfälische Wilhelms-Universität Münster Corrensstraße 40 48149 Münster Germany; ^3^ Medizinische Fakultät Universität Köln Gleueler Straße 24 50931 Köln Germany; ^4^ Cells-in-Motion Cluster of Excellence (EXC 1003–CiM) Westfälische Wilhelms-Universität Münster Waldeyerstraße 15 48149 Münster Germany

**Keywords:** CK2 inhibitors, enzyme inhibition, epimerization, kinases, stereochemistry, tetracyclic systems

## Abstract

The serine/threonine kinase CK2 modulates the activity of more than 300 proteins and thus plays a crucial role in various physiological and pathophysiological processes including neurodegenerative disorders of the central nervous system and cancer. The enzymatic activity of CK2 is controlled by the equilibrium between the heterotetrameric holoenzyme CK2α_2_β_2_ and its monomeric subunits CK2α and CK2β. A series of analogues of W16 ((3a*R*,4*S*,10*S*,10a*S*)‐4‐{[(*S*)‐4‐benzyl‐2‐oxo‐1,3‐oxazolidin‐3‐yl]carbonyl}‐10‐(3,4,5‐trimethoxyphenyl)‐4,5,10,10a‐tetrahydrofuro[3,4‐*b*]carbazole‐1,3(3a*H*)‐dione ((+)‐**3** **a**)) was prepared in an one‐pot, three‐component Levy reaction. The stereochemistry of the tetracyclic compounds was analyzed. Additionally, the chemically labile anhydride structure of the furocarbazoles **3** was replaced by a more stable imide (**9**) and *N*‐methylimide (**10**) substructure. The enantiomer (−)‐**3** **a** (*K*_i_=4.9 μM) of the lead compound (+)‐**3** **a** (*K*_i_=31 μM) showed a more than sixfold increased inhibition of the CK2α/CK2β interaction (protein‐protein interaction inhibition, PPII) in a microscale thermophoresis (MST) assay. However, (−)‐**3** **a** did not show an increased enzyme inhibition of the CK2α_2_β_2_ holoenzyme, the CK2α subunit or the mutated CK2α′ ^C336S^ subunit in the capillary electrophoresis assay. In the pyrrolocarbazole series, the imide (−)‐**9** **a** (*K*_i_=3.6 μM) and the *N*‐methylimide (+)‐**10** **a** (*K*_i_=2.8 μM) represent the most promising inhibitors of the CK2α/CK2β interaction. However, neither compound could inhibit enzymatic activity. Unexpectedly, the racemic tetracyclic pyrrolocarbazole (±)‐**12**, with a carboxy moiety in the 4‐position, displays the highest CK2α/CK2β interaction inhibition (*K*_i_=1.8 μM) of this series of compounds.

## Graphical abstract

**No get‐together**: Tetracyclic inhibitors of the protein‐protein interaction of the protein kinase CK2 subunits to form the tetrameric holoenzyme have been developed. Particular attention was paid to the stereochemistry and the group X resulting in furo‐ and pyrrolocarbazoles. Some furocarbazoles showed PPII in the low‐micromolar range, inhibition of the tetrameric holoenzyme and weak inhibition of two subunits.

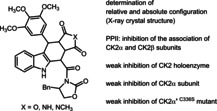

## Introduction

A multitude of physiological processes is affected by the phosphorylation state of proteins, which is controlled by various protein kinases and phosphatases.^1^ Introduction of the very polar phosphate moiety induces a conformational change of the protein and thus a modification of its biological and pharmacological properties. In particular, phosphorylation of substrate proteins displays a very important mechanism of inter‐ and intracellular signal transduction. Moreover, kinases control cellular processes such as metabolism, transcription, and cell cycle.^2^, ^3^, ^4^ Due to their key role in several physiological processes, various protein kinases emerged as promising targets for the development of novel drugs.^5^, ^6^ The kinase inhibitor imatinib, which was introduced into the market in 2001, paved the way for the development of protein kinase inhibitors. Today, such inhibitors are approved for the treatment of cancer, inflammation and rheumatoid arthritis.^7^, ^8^

The protein kinase CK2 (previously known as casein kinase 2, CK2) was detected in several eukaryotic organisms.^9^, ^10^ The human CK2 transfers a phosphate moiety from ATP or GTP to serine or threonine residues in various proteins.^11^ It forms a heterotetramer consisting of two CK2α and two CK2β subunits. The monomeric subunits and the tetrameric holoenzyme are postulated to be in an equilibrium, which controls the enzymatic properties of CK2 in the cell.^12^ Each CK2α subunit is bound to a CK2β dimer, forming contacts with both CK2β subunits.^13^ The CK2α subunit contains the ATP binding site and is able to transfer a phosphate group to a substrate, even as a monomer.^14^ However, stability, catalytic activity and selectivity of CK2α are regulated by the CK2β subunit,^15^ with enzymatic activity and substrate spectrum being different from those of the tetrameric holoenzyme.^16^ According to Pinna,^17^ three classes of CK2 substrates are distinguished on the basis of the quaternary structure of the active enzyme. Class I substrates (e. g., inhibitor‐2 of protein phosphatase‐1) are phosphorylated by both the CK2 holoenzyme and the CK2α/CK2α‘ subunits, whereas class II substrates, such as calmodulin, are exclusively accepted by the CK2α/CK2α‘ subunits.^18^ Addition of CK2β subunit inhibits the phosphorylation of the latter substrates, which is mitigated by polycationic compounds, such as polylysine. Both effects were shown to be mediated by the N‐terminal acidic loop (D^55^LEPDEELED^64^) of CK2β.^19^ In contrast, phosphorylation of class III substrates, among them eukaryotic initiation factor 2β (eIF2β) and pancreatic transcription factor (PDX‐1),^20^, ^21^ requires the presence of the CK2β subunit with an intact N‐terminal acidic loop.^22^ The acidic loop is thought to interact with a cluster of basic residues in the class III substrates located up to 33 residues away from the phosphoacceptor site, with addition of polycations preventing holoenzyme‐dependent substrate phosphorylation most probably by competing with the basic cluster of the substrate for binding to the acidic loop of CK2β.^22^, ^23^, ^24^

The presence of CK2 is essential for the survival of eukaryotic cells, since it modulates the activity of more than 300 proteins by phosphorylation.^25^, ^26^ Due to the large number of substrates accepted by CK2, it plays a crucial role in various physiological and pathophysiological processes including regulation of the cell cycle, proliferation, angiogenesis, repairing of DNA damage, embryogenesis, suppression of apoptosis and influence on CNS activity.^27^, ^28^, ^29^, ^30^, ^31^, ^32^, ^33^

**Figure 1:**
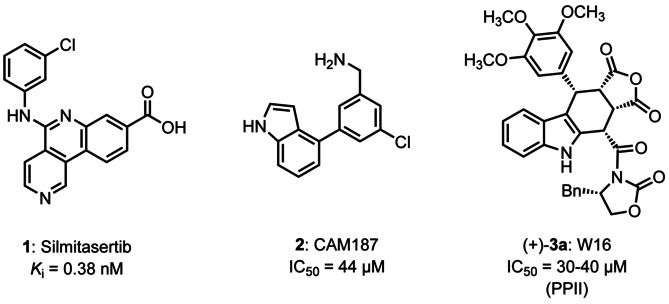
CK2 inhibitors: silmitasertib (**1**, CX‐4945) is an ATP‐competitive CK2 inhibitor; CAM187 (**2**) binds selectively to the CK2α subunit and inhibits its association with the CK2β subunit (protein‐protein interaction inhibition); W16 (**3** **a**) inhibits the association of the CK2α and CK2β subunits and thus modulates the kinase activity and selectivity of the CK2α subunit.

It has been shown that CK2 is involved in several CNS disorders. An increased expression of CK2 was observed in neurons containing pathological neurofibrillary tangles (Alzheimer's disease)^34^, ^35^ and it contributes to the formation of Levy bodies (Parkinson's disease).^36^, ^37^ In particular, its role in the development of cancer is well investigated.^38^ CK2 is upregulated in almost all kinds of cancer, promoting cell proliferation and preventing apoptosis.^38^, ^39^, ^40^, ^41^, ^42^ Because of the high number of substrates and its constitutive activity, CK2 was considered to be “non‐druggable” for many years. Recently, the development of the CK2 inhibitor silmitasertib (**1**, CX‐4945, Figure 1) refuted this assumption.^43^ The ATP‐competitive CK2 inhibitor **1** is in late stage clinical trials for the treatment of cholangiocarcinoma (bile duct cancer) and in early clinical trials for the treatment of various other cancers including hematological and lymphoid malignancies.^44^

In general, kinase inhibitors competing with ATP for binding to the ATP binding site (e. g., silmitasertib) suffer from low selectivity, since the ATP binding site is highly conserved in different kinases.^45^ Inhibition of the interaction between the CK2α and CK2β subunits (protein‐protein interaction, PPI) represents a further strategy to inhibit the kinase CK2.^46^ Very recently, the non‐ATP‐competitive ligand CAM187 (**2**, Figure 1) was reported to bind to the CK2α subunit and inhibit the interaction with the CK2β subunit (IC_50_=44 μM). Although **2** was able to affect this PPI, it did not significantly inhibit CK2 activity.^47^ The same behavior was observed for the cyclic 13 amino acid peptide Pc and its derivatives, which were designed as CK2β mimetics to inhibit CK2 subunit association.^48^, ^49^, ^50^

In this project, the furocarbazole W16 ((+)‐3a, Figure 1) served as lead compound for the optimization of the inhibition of CK2α and CK2β subunit interaction. With an IC_50_ value of 30–40 μM, W16 ((+)‐3a) shows weak inhibition of CK2α/CK2β interaction. On the other hand, inhibition (IC_50_=20 μM) of the catalytic activity of monomeric CK2α was observed.^51^ Compound (+)‐3a with the rigid tetracyclic furocarbazole framework represents a rather large molecule, which violates Lipinski's rule of 5.^52^ In particular its molecular weight of 610 Da exceeds the defined upper limit of 500 Da. However, for inhibition of PPIs larger molecules are required (e. g., cyclic peptide Pc has a molecular weight of 1,409 Da^49^), since the compounds are supposed to interact with the large surface of a protein rather than with a deep binding pocket. The furocarbazole (+)‐3a is a very good starting point, since it allows diverse structural modifications.^53^ In this report, we will focus on the furan moiety and the stereochemistry, since the relationships between the configuration and the PPI inhibition (PPII) within this compound class have not been investigated yet.

## Results and Discussion

### Synthesis of tetracyclic furo‐ and pyrrolocarbazoles

**Scheme 1:**
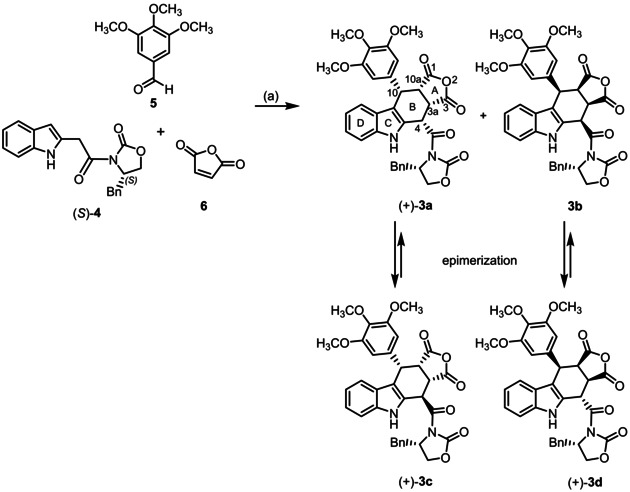
Synthesis of furocarbazole derivatives (+)‐**3** **a**, (+)‐**3** **c** and (+)‐**3** **d**. Reagents and reaction conditions: a) CuSO_4_^.^5H_2_O, toluene, reflux, 24 h. Absolute configuration of the products: (+)‐**3** **a**: S‐3aR,4S,10S,10aS; **3** **b**: S‐3aS,4R,10R,10aR; (+)‐**3** **c**: S‐3aR,4R,10S,10aS; (+)‐**3** **d**: S‐3aS,4S,10R,10aR.

The furocarbazole derivative (+)‐**3** **a** had been synthesized as shown in Scheme 1 by Levy reaction of (indol‐2‐yl)acetamide (S)‐**4**, 3,4,5‐trimethoxybenzaldehyde (**5**) and maleic anhydride (**6**).^51^, ^54^ To compare both the stereochemistry and the biological activity of new analogues with those of the lead compound (+)‐**3** **a**, the Levy reaction should be performed as reported in literature.^51^, ^54^ For this purpose, (indol‐2‐yl)acetamide (S)‐**4** was prepared by reaction of (2‐nitrophenyl)acetyl chloride with Meldrum's acid, followed by aminolysis of the reactive triacyl intermediate with (*S*)‐4‐benzyloxazolidin‐2‐one and final reduction with Zn/NH_4_Cl (details see the Supporting Information).^54^, ^55^

The three‐component Levy reaction of (indol‐2‐yl)acetamide (*S*)‐**4**, benzaldehyde **5** and maleic anhydride (**6**) in the presence of CuSO_4_^.^5H_2_O at reflux temperature for 24 h led to a mixture of diastereomeric products **3**. (Scheme1) Column chromatographic separation of the mixture resulted in pure (+)‐**3** **c** and a mixture of (+)‐**3** **a** and (+)‐**3** **d**, which were separated by recrystallization. Unexpectedly, the fourth diastereomer **3** **b** could not be detected or isolated.

**Figure 2:**
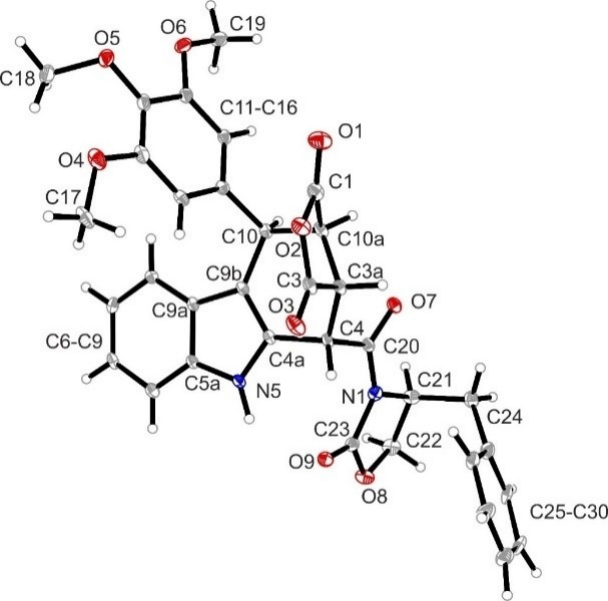
X‐ray crystal structure of (+)‐**3** **d**. Compound (+)‐**3** **d** crystallized in the orthorhombic space group *P*2_1_2_1_2_1_. Thermal ellipsoids are shown with 20  % probability. The *S* configuration of C21 in the oxazolidine ring and *cis*,*cis*,*trans*‐configuration of the substituents in ring B are shown (*S*‐3a*S*,4*S*,10*R*,10a*R* configuration). The Flack parameter was refined to 0.1(2).

The structure including the stereochemistry of (+)‐**3** **a** was confirmed by comparison of its spectroscopic data with those given in the literature for the lead compound (+)‐**3** **a**.^51^ The absolute configuration of (+)‐**3** **d** was determined unequivocally by X‐ray crystal structure analysis. (Figure 2) Moreover, the *cis*,*cis*,*trans*‐configuration of the substituents at ring B was confirmed by the down‐field shift of the 4‐H signal (*δ*=6.02 ppm) and the small coupling constant between 4‐H and 3a‐H (*J*=1.8 Hz; Table S1 in the Supporting Information).^54^, ^56^ Similar NMR data obtained for (+)‐**3** **c** (*δ*(4‐H)=6.07 ppm, *J*(4‐H/3a‐H)=2.7 Hz, Table S1) clearly show the same relative *cis*,*cis*,*trans*‐configuration of the substituents in ring B. As the *S* configuration of C‐4 in the oxazolidine ring is pre‐defined by the reactant (*S*)‐**4**, the absolute *S*‐3a*R*,4*R*,10*S*,10a*S* configuration for (+)‐**3** **c** was unequivocally assigned.

According to the mechanism of the Levy reaction including a Diels‐Alder reaction as key step, the *cis*,*cis*,*cis*‐configured stereoisomers (+)‐**3** **a** and **3** **b** are expected to be the primary products. However, it has been shown that high temperature, prolonged reaction times, polar solvents and bases lead to epimerization of the kinetically formed *cis*,*cis*,*cis*‐configured diastereomers into the thermodynamically favored *cis*,*cis*,*trans*‐configured diastereomers.^56^ This epimerization nicely explains the formation of the *cis*,*cis*,*trans*‐configured products (+)‐**3** **c** and (+)‐**3** **d**. A complete epimerization of **3** **b** may be responsible for the exclusive isolation of (+)‐**3** **d**.

The enantiomer (−)‐**3** **a** was prepared in the same manner (36 % yield) starting the Levy reaction with the enantiomer (*R*)‐**4**. Here, the corresponding diastereomers (−)‐**3** **c** and (−)‐**3** **d** could not be isolated. Altogether, the high reactivity (instability) and fast epimerization which already occurred during recrystallization experiments of **3** stimulated the bio‐isosteric replacement of the cyclic anhydride substructure by cyclic imides.

**Scheme 2:**
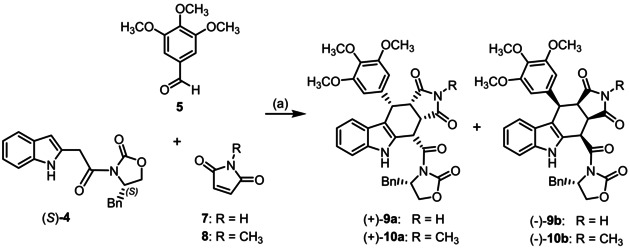
Synthesis of cis,cis,cis‐configured pyrrolocarbazole derivatives (+)‐**9** **a**, (‐)‐**9** **b**, (+)‐**10** **a**, and (‐)‐**10** **b**. Reagents and reaction conditions: a) CuSO_4_^.^5H_2_O, toluene, 130 °C (pressure resistant Schlenk flask), 16–22 h. (+)‐**9** **a** (28 %), (−)‐**9** **b** (36 %), (+)‐**10** **a** (26 %), (−)‐**10** **b** (37 %). Absolute configuration of the products: (+)‐**9** **a**, (+)‐**10** **a**: S‐3aR,4S,10S,10aS configuration; (−)‐**9** **b**, (‐)‐**10** **b** S‐3aS,4R,10R,10aR configuration. The enantiomers (−)‐**9** **a**, (+)‐**9** **b**, (−)‐**10** **a**, and (+)‐**10** **b** were prepared in the same manner.

Thus, reaction of (indol‐2‐yl)acetamide (S)‐**4** with 3,4,5‐trimethoxybenzyladehyde (**5**) and maleimide (**7**) or *N*‐methylmaleimide (**8**) in boiling toluene provided the diastereomeric *cis*,*cis*,*cis*‐configured pyrrolocarbazoles (+)‐**9** **a**/(−)‐**9** **b** and (+)‐**10** **a**/(−)‐**10** **b**, respectively. (Scheme 2) The corresponding enantiomers (−)‐**9** **a**, (+)‐**9** **b**, (−)‐**10** **a**, and (+)‐**10** **b** were prepared by the same Levy‐reaction using enantiomeric (indol‐2‐yl)acetamide (*R*)‐**4** as starting material.

**Figure 3:**
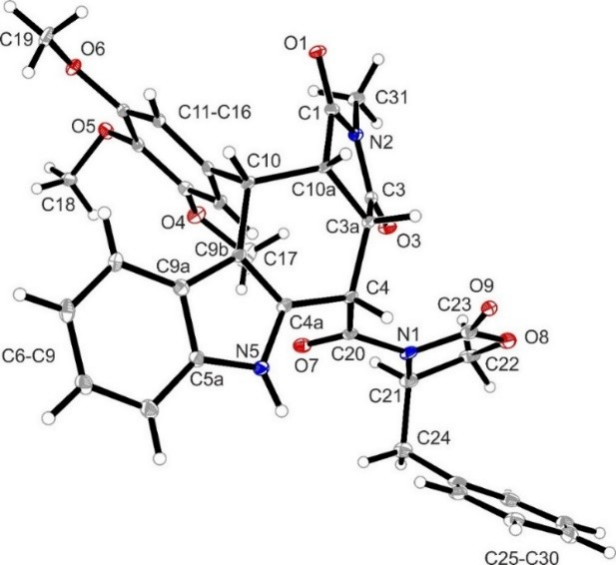
X‐ray crystal structure of (+)‐**10** **b**. Compound (+)‐**10** **b** crystallized in the hexagonal space‐group *P*6_*5*_. Thermal ellipsoids are shown with 20 % probability. The *R* configuration of C21 in the oxazolidine ring and *cis*,*cis*,*cis*‐configuration of the substituents in ring B are shown (*R*‐3a*S*,4*S*,10*S*,10a*S* configuration). The Flack parameter was refined to 0.04(9).

As described above, the relative *cis*,*cis*,*cis*‐configuration of the tetracyclic pyrrolocarbazoles **9** **a**,**b** and **10** **a**,**b** was confirmed by the high‐field shift of the 4‐H signal (*δ*=5.35–5.51 ppm) and the large coupling constant between 4‐H and 3a‐H (*J*=11.5–11.6 Hz). However, an X‐ray crystal structure analysis was needed to correctly assign the absolute configuration. In Figure 3, the crystal structure of (+)‐**10** **b** is displayed proving the absolute *R*‐3a*S*,4*S*,10*S*,10a*S* configuration. Based on this absolute configuration of (+)‐**10** **b**, the absolute configuration of the other stereoisomers (+)‐**10** **a**, (−)‐**10** **a** and (−)‐**10** **b** as well as that of the corresponding maleimide derivatives (+)‐**9** **a**, (+)‐**9** **a**, (+)‐**9** **b** and (−)‐**9** **b** could be assigned unambiguously by comparing spectroscopic data and specific optical rotation of the compounds (Table S2).

During purification and recrystallization, the pyrrolocarbazoles **9** **a**,**b** and **10** **a**,**b** turned out to be much more stable than the corresponding furocarbazoles **3**. Formation of C‐4 epimers was not observed. Even heating to reflux of an acetonitrile solution of (+)‐**10** **a** with and without DIPEA or TFA led only to small amounts of C‐4‐epimer (analyzed by ^1^H NMR spectroscopy). Since this epimerization was accompanied by the formation of several side products, another strategy was pursued for the synthesis of the corresponding *cis*,*cis*,*trans*‐configured stereoisomers **9** **c** and **9** **d**.

**Scheme 3:**
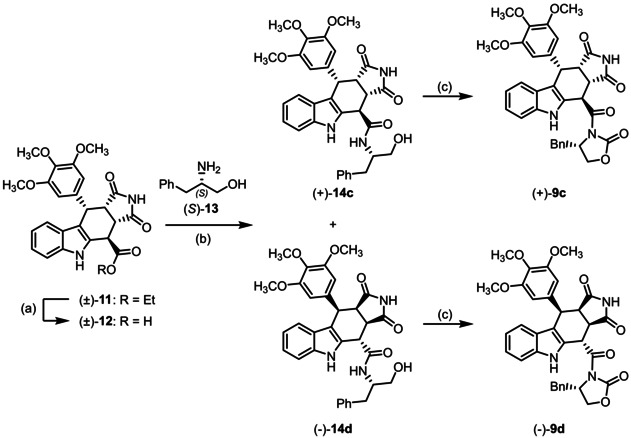
Synthesis of cis,cis,trans‐configured pyrrolocarbazole derivatives (+)‐**9** **c** and (−)‐**9** **d**. Reagents and reaction conditions: a) NaOH, H_2_O, THF, RT, 30 min, 96 %; b) 1. (COCl)_2_, DMF, CH_2_Cl_2_, RT, 3 h, concentration in vacuo; 2. (S)‐**13**, DMF, CH_2_Cl_2_, DIPEA; RT, 1 h, (+)‐**14** **c** (28 %), (−)‐**14** **d** (26 %); c) CDI, DMF, 60 °C, 16 h. (+)‐**9** **c** (20 %), (‐)‐**9** **d** (42 %). Absolute configuration of the products: (+)‐**9** **c**: S‐3aS,4R,10S,10aS configuration; (−)‐**9** **d** S‐3aR,4S,10R,10aR configuration. The enantiomers (−)‐**9** **c** and (+)‐**9** **d** were prepared in the same manner.

For the synthesis of *cis*,*cis*,*trans*‐configured stereoisomers **9** **c** and **9** **d** (Scheme 3), racemic tetracyclic ester (±)‐**11** 55, 56 was hydrolyzed with NaOH to afford carboxylic acid (±)‐**12** in 96 % yield. Activation of acid (±)‐**12** with oxalyl chloride and subsequent coupling with *S*‐configured phenylalaninol (*S*)‐**13** led to enantiomerically pure amides (+)‐**14** **c** and (−)‐**14** **d**. The oxazolidinone moiety of (+)‐**9** **c** and (−)‐**9** **d** was established by cyclization of the amidoalcohols (+)‐**14** **c** and (−)‐**14** **d** with CDI.

**Figure 4:**
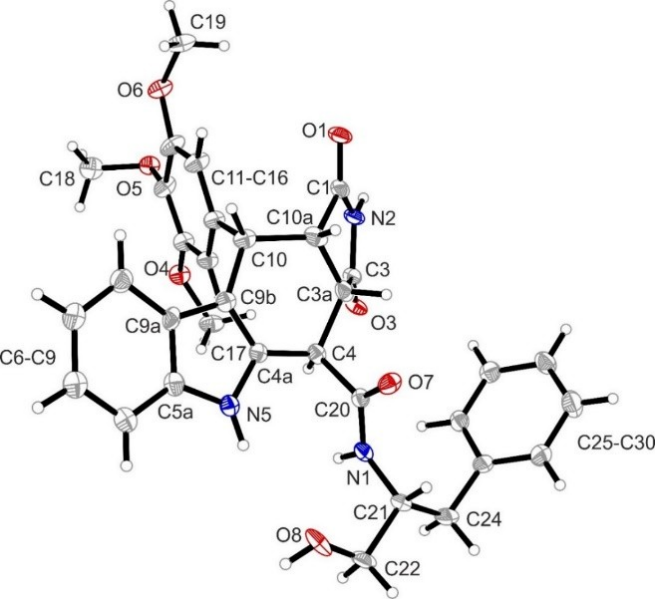
X‐ray crystal structure of (+)‐**14** **d**. Compound (+)‐**14** **d** crystallized in the monoclinic space group *P*2_1_. Thermal ellipsoids are shown with 20 % probability. The *R* configuration of C21 of the *N*‐(3‐hydroxy‐1‐phenylpropan‐2‐yl) substituent and *cis*,*cis*,*trans*‐configuration of the substituents in ring B are shown (*R*‐3a*S*,4*R*,10*S*,10a*S* configuration). The Flack parameter was refined to 0.02(9).

The relative and absolute configuration of (+)‐**14** **d** was determined by X‐ray crystal structure analysis. The structure of (+)‐**14** **d** displayed in Figure 4 clearly shows *cis*,*cis*,*trans*‐configuration of the substituents at ring B. Moreover, the *R* configuration of the N‐substituent coming from (*R*)‐phenylalaninol (*R*)‐**13** and 3a*S*,4*R*,10*S*,10a*S* configuration of the four centers of chirality in the tetracyclic ring system of (+)‐**14** **d** are shown. Careful comparison of NMR spectra including ROESY 2D spectra allowed the unequivocal assignment of the absolute configuration for the remaining isomers (−)‐**14** **d**, (+)‐**14** **c**, and (‐)‐**14** **c** as well as for the following products (+)‐**9** **d**, (−)‐**9** **d**, (+)‐**9** **c**, and (‐)‐**9** **c**. Exemplarily, the enantiomeric purity of pyrrolocarbazoles (+)‐**9** **c**, (‐)‐**9** **c**, (+)‐**9** **d**, and (‐)‐**9** **c** was analyzed by chiral HPLC using a Daicel Chiralpak^®^ IA column. All tested compounds show high enantiomeric purity (Table S3).

### Pharmacological evaluation

#### Inhibition of the CK2α/CK2β interaction

The inhibition of the interaction between the CK2α and the CK2β subunit was determined in a microscale thermophoresis (MST) assay. At first the *K*_D_ value of the CK2α/CK2β interaction was determined by addition of a constant amount of fluorescently labeled CK2β subunit to increasing concentrations of the CK2α subunit and by analysis of the thermophoretic shift. This experiment led to a *K*_D_ value of 12±1 nM (*n*=4) for the CK2α/CK2β interaction.^57^ A significantly increased dissociation constant *K*_D_’ of the two CK2 subunits in the presence of test compound (50 or 100 μM) indicated an inhibition of the CK2α/CK2β interaction: (Table S4) Because of a low solubility of some derivatives, the test compounds were also investigated at lower concentrations (10 or 20 μM), which, however, resulted in non‐significantly changed dissociation constant *K*_D_’. (Table S4) A re‐analysis of all those MST experiments with significantly increased values of *K*_D_’ by fitting the *K*_D_ value of the CK2α/CK2β‐interaction in the absence of test compound to a global value (*K*_D_=11 nM) yielded *K*_i_ values for the PPII by the test compounds which are summarized in Table 1.

**Table 1:** CK2α/CK2β interaction inhibition and inhibition of the activities of holoenzyme CK2α_2_β_2_, the CK2α subunit and the mutated CK2α’ ^C336S^ subunit.

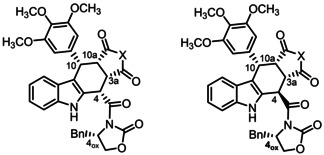								
Compd.	X	Config. 4_ox_	Config. 3a‐4‐10‐10a	Inhibition of CK2α/CK2β_2_ interaction *K*_i_ [μM]^[a]^	Inhibition (%) of CK2α_2_β_2_ [c=10 μM]^[b]^	Inhibition of CK2α_2_β_2_ IC_50_ [μM]^[b]^	Inhibition (%) of CK2α [c=10 μM]^[b]^	Inhibition (%) of CK2α’ ^C336S^ [*c*=10 μM]^[b]^
(+)‐**3** **a** (W‐16)	O	*S*	*R*‐*S*‐*S*‐*S*	31±14	84±5	1.9	76±4	85±13
(−)‐**3** **a**	O	*R*	*S*‐*R*‐*R*‐*R*	4.9±1.8	89±3	2.7	73±4	93±2
(+)‐**3** **c**	O	*S*	*R*‐*R*‐*S*‐*S*	prec.	66±16	6.5	65±4	52±38
(+)‐**3** **d**	O	*S*	*S*‐*S*‐*R*‐*R*	42±6	92±7	3.8	43±13	n.s.
(+)‐**9** **a**	NH	*S*	*S*‐*S*‐*S*‐*S*	n.s.	20±4	n.d.	n.s.	n.s.
(−)‐**9** **a**	NH	*R*	*R*‐*R*‐*R*‐*R*	3.6±0.6	n.s.	n.d.	n.s.	n.s.
(+)‐**9** **b**	NH	*R*	*S*‐*S*‐*S*‐*S*	4.9±0.8	12±7	n.d.	n.s.	n.s.
(−)‐**9** **b**	NH	*S*	*R*‐*R*‐*R*‐*R*	4.4±0.3	n.s.	n.d.	n.s.	n.s.
(+)‐**9** **c**	NH	*S*	*S*‐*R*‐*S*‐*S*	prec.	<50	n.d.	n.d.	n.d.
(‐)‐**9** **c**	NH	*R*	*R*‐*S*‐*R*‐*R*	prec.	<50	n.d.	n.d.	n.d.
(+)‐**9** **d**	NH	*R*	*S*‐*R*‐*S*‐*S*	n.s.	<50	n.d.	n.d.	n.d.
(−)‐**9** **d**	NH	*S*	*R*‐*S*‐*R*‐*R*	n.s.	<50	n.d.	n.d.	n.d.
(+)‐**10** **a**	NCH_3_	*S*	*S*‐*S*‐*S*‐*S*	2.8±0.9	26±7	n.d.	41±11	53±15
(−)‐**10** **a**	NCH_3_	*R*	*R*‐*R*‐*R*‐*R*	8.5±2.9	29±14	n.d.	n.s.	30±23
(+)‐**10** **b**	NCH_3_	*R*	*S*‐*S*‐*S*‐*S*	7.2±1.5	31±9	n.d.	n.s.	n.s.
(−)‐**10** **b**	NCH_3_	*S*	*R*‐*R*‐*R*‐*R*	3.7 ± 0.7	15±11	n.d.	n.s.	n.s.
(±)‐**11**	NH	CO_2_Et	*RS‐SR‐RS‐RS*	32±17	n.s.	n.d.	n.s.	n.s.
(±)‐**12**	NH	CO_2_H	*RS‐SR‐RS‐RS*	1.8±0.8	n.s.	n.d.	n.s.	n.s.
(+)‐**14** **c**	NH	*S*	*S*‐*R*‐*S*‐*S*	n.s.	<50	n.d.	n.d.	n.d.
(−)‐**14** **c**	NH	*R*	*R‐S‐R‐R*	n.s.	<50	n.d.	n.d.	n.d.
(+)‐**14** **d**	NH	*R*	*S*‐*R*‐*S*‐*S*	n.s.	<50	n.d.	n.d.	n.d.
(−)‐**14** **d**	NH	*S*	*R‐S‐R‐R*	n.s.	<50	n.d.	n.d.	n.d.

[a] Mean±SEM values of 2–4 separate experiments resulting from a global fit of all included data sets. A global *K*_D_ value of 11 nM was calculated for the CK2α/CK2β‐interaction in this global analysis. [b] Mean value±standard deviation (SD) of three independent experiments. *prec.=precipitation; n.s.=not significant; n.d.=not determined

In order to ensure the validity of the MST method for determination of *K*_i_ values of test compounds at the CK2α/CK2β interaction site, the lead compound W16 ((+)‐**3** **a**) was prepared and pharmacologically evaluated. In the MST assay, a *K*_i_ value of 31 μM was found which is very close to the reported IC_50_ value of 30–40 μM.^51^ The diastereomer (+)‐**3** **d** displayed a similar affinity as (+)‐**3** **a**. Unfortunately, the inhibition of the CK2α/CK2β interaction by the stereoisomer ((+)‐**3** **c** could not be recorded due to solubility problems. The solubility of these relatively large molecules (molecular weight, MW, of 610 Da and, thus, exceeding Lipinski's upper limit of MW=500 Da) is a general problem. Unexpectedly, the enantiomer (−)‐**3** **a** revealed an approximately sixfold increased inhibition of the CK2α/CK2β interaction compared to the lead compound W16 ((+)‐**3** **a**). This result clearly indicates the major role of the stereochemistry of these complex molecules on their biological activity (Table 1).

Due to the high reactivity (low stability) of ligands **3**, the reactive anhydride substructure of **3** was replaced by a chemically more stable imide substructure. In the class of secondary imides **9**, strong inhibition of the CK2α/CK2β interaction was observed, in particular for those compounds with the same stereochemistry as (+)‐**3** **a** and (‐)‐**3** **a**. (It should be noted, that the stereodescriptor for the center of chirality in 3a‐position is changed upon exchange of the O‐atom by a N‐atom.) The affinities of (−)‐**9** **a** (*K*_i_=3.6 μM) and (+)‐**9** **b** (*K*_i_=4.9 μM) are in the same low‐micromolar range as the dissociation constant of (−)‐**3** **a** (*K*_i_=4.9 μM). Again, some of the test compounds ((+)‐**9** **c** and (−)‐**9** **c**) precipitated during the MST assay due to low solubility (Table 1).

Analysis of the inhibition of the CK2α/CK2β interaction by the tertiary imides **10** containing an additional methyl moiety at the N‐atom also resulted in dissociation constants in the low micromolar range, with (+)‐**10** **a** (*K*_i_=2.8 μM) and (−)‐**10** **b** (*K*_i_=3.7 μM) being the most potent tertiary imides (Table 1).

It can be concluded that chemical stabilization of the anhydrides **3** by imides **9** and **10** is well tolerated without loss of inhibition of the CK2 subunit interaction. Moreover, the stereochemistry of the tetracyclic system appears to be crucial for the biological activity.

In addition to the final products **3**, **9**, and **10**, the biological activity of the racemic ester (±)‐**11**, the racemic carboxylic acid (±)‐**12** and the 2‐hydroxyethylamides **14** was assessed in the MST assay. With a *K*_i_ value of 1.8 μM the racemic acid (±)‐**12** exhibited the most potent inhibition of the CK2α/CK2β interaction among the analyzed compounds, whereas the intermediates **14** showed no activity (Table 1).

#### Inhibition of the enzymatic activity

In addition to analyzing the influence of the test compounds on the CK2α/CK2β interaction, the inhibition of the enzymatic activity of the holoenzyme (CK2α_2_β_2_), the CK2α subunit, and the mutated CK2α’ ^C336S^ subunit was investigated in a capillary electrophoresis (CE) assay. In this assay, the decapeptide RRRDDDSDDD, a known class I substrate suitable for assaying the kinase activity of both the CK2 holoenzyme and the catalytically active CK2 subunits,^50^, ^58^ was reacted with ATP in the presence of enzyme, which transfers a phosphate group to Ser7 of the decapeptide. Due to the additional negative charge of the phosphate group, the decapeptide and the phosphorylated decapeptide can be separated by CE. A reduced amount of phosphorylated decapeptide, that is, product, indicates inhibition of the kinase.^59^ The mutated CK2α’ ^C336S^ subunit was included into this study, since this subunit has also kinase activity and can also form an active holoenzyme, but is more stable than the wild‐type CK2α’ subunit. The data on the inhibition of the kinase activities of CK2α_2_β_2_, CK2α subunit and mutated CK2α’ ^C336S^ subunit are summarized in Table 1.

In the first screening with a concentration of test compound of 10 μM only some of the stereoisomeric furocarbazoles **3** showed more than 50 % inhibition of the CK2 holoenzyme, the CK2α subunit, and the mutated CK2α′ ^C336S^ subunit. Therefore, IC_50_ values for the inhibition of CK2α_2_β_2_ were determined only for the furocarbazoles **3**. Interestingly, the lead compound (+)‐**3** **a** (W‐16) exhibited the strongest inhibition of CK2α_2_β_2_ (IC_50_=1.9 μM). The enantiomer (−)‐**3** **a** (IC_50_=2.7 μM) was only slightly less potent and the inhibitory activities of the diastereomers (+)‐**3** **c** and (+)‐**3** **d** were also in the same range.

The analogous imides **9** and **10**, the synthesis educts (±)‐**11**, (±)‐**12** and the 2‐hydroxyethylamides **14** did not inhibit the kinases at a relevant extend.

## Conclusion

In order to investigate SAR, four stereoisomeric furocarbazoles **3** resulting from partial epimerization at C‐4, eight stereoisomeric pyrrolocarbazoles **9** and four stereoisomeric *N*‐methyl‐pyrrolocarbazoles **10** were prepared in an one‐pot, three‐component Levy reaction. The relative and absolute configuration of the different products was assigned unequivocally by X‐ray crystal structure analysis and NMR spectroscopy.

The stereochemistry has a high impact on the CK2α/CK2β‐interaction inhibition (protein protein interaction inhibition, PPII) as the enantiomer (−)‐**3** **a** (*K*_i_=4.9 μM) is more than 6‐fold more active than the lead compound (+)‐**3** **a** (*K*_i_=31 μM) in the MST assay. The rather labile anhydride structure of the lead compound (+)‐**3** **a** was replaced by chemically and biologically more stable imide and *N*‐methylimide substructure resulting in pyrrolocarbazoles (−)‐**9** **a** (*K*_i_=3.6 μM) and (+)‐**10** **a** (*K*_i_=2.8 μM), respectively, with stronger CK2α/CK2β‐interaction inhibition than those of the furocarbazoles **3**. Therefore, it was concluded that replacement of the anhydride structure (O) of **3** by an imide (NH, **9**) or *N*‐methylimide (NCH_3_, **10**) does not only increase the chemical and biological stability but also the inhibition of the CK2α/CK2β‐interaction.

However, the increased PPII of the stereoisomeric imide analogues **9** and **10** did not result in stronger enzyme inhibition. In the CE assay, the stereoisomeric imides **9** and **10** were not able to inhibit the kinase, neither the holoenzyme CK2α_2_β_2_ nor the CK2α subunit nor the mutated CK2α′ ^C336S^ subunit. Obviously, intervention into the association‐dissociation equilibrium of the CK2 holoenzyme CK2α_2_β_2_ does not necessarily lead to higher enzyme inhibition.

For some of the synthesized furocarbazoles **3** and pyrrolocarbazoles **9** and **10**, inhibition of the CK2α/CK2β interaction as well as the effect on the activities of the CK2α_2_β_2_ holoenzyme and that of the CK2α subunits could not be recorded due to precipitation of the compounds during dilution or during the assays.

## Experimental Section

### Chemistry, general methods

Oxygen and moisture sensitive reactions were carried out under nitrogen dried with silica gel with moisture indicator (orange gel, VWR, Darmstadt, Germany) and in dry glassware (Schlenk flask or Schlenk tube). Temperature was controlled with dry ice/acetone (−78 °C), ice/water (0 °C), Cryostat (Julabo TC100E‐F, Seelbach, Germany), magnetic stirrer MR 3001 K (Heidolph, Schwalbach, Germany) or RCT CL (IKA, Staufen, Germany), together with temperature controller EKT HeiCon (Heidolph) or VT‐5 (VWR) and PEG or silicone bath. All solvents were of analytical or technical grade quality. *o*‐Xylene and toluene were dried with molecular sieves (3 Å). Demineralized water was used. Thin layer chromatography (tlc): tlc silica gel 60 F_254_ on aluminum sheets (VWR). Flash chromatography (fc): Silica gel 60, 40–63 μm (VWR); parentheses include: diameter of the column (Ø), length of the stationary phase (l), fraction size (v) and eluent. Automated flash chromatography: Isolera^TM^ Spektra One (Biotage^®^); parentheses include: cartridge size, flow rate, eluent, fractions size was always 20 mL. Melting point: Melting point system MP50 (Mettler Toledo, Gießen, Germany), open capillary, uncorrected. MS: MicroTOFQII mass spectrometer (Bruker Daltonics, Bremen, Germany); deviations of the found exact masses from the calculated exact masses were 5 mDa or less; the data were analyzed with DataAnalysis^®^ (Bruker Daltonics). NMR: NMR spectra were recorded in deuterated solvents on Agilent DD2 400 MHz and 600 MHz spectrometers (Agilent, Santa Clara CA, USA); chemical shifts (*δ*) are reported in parts per million (ppm) against the reference substance tetramethylsilane and calculated using the solvent residual peak of the undeuterated solvent; coupling constants are given with 0.5 Hz resolution; assignment of ^1^H and ^13^C NMR signals was supported by 2‐D NMR techniques where necessary. IR: FT/IR Affinity^®^‐1 spectrometer (Shimadzu, Düsseldorf, Germany) using ATR technique.

### HPLC method for the determination of the purity

Equipment 1: Pump: L‐7100, degasser: L‐7614, autosampler: L‐7200, UV detector: L‐7400, interface: D‐7000, data transfer: D‐line, data acquisition: HSM‐Software (all from Merck Hitachi, Darmstadt, Germany); Equipment 2: Pump: LPG‐3400SD, degasser: DG‐1210, autosampler: ACC‐3000T, UV‐detector: VWD‐3400RS, interface: DIONEX UltiMate 3000, data acquisition: Chromeleon 7 (equipment and software from Thermo Fisher Scientific, Lauenstadt, Germany); column: LiChrospher^®^ 60 RP‐select B (5 μm), LiChroCART^®^ 250–4 mm cartridge; flow rate: 1.0 mL/min; injection volume: 5.0 μL; detection at *λ*=210 nm; solvents: A: demineralized water with 0.05 % (*v/v*) trifluoroacetic acid, B: CH_3_CN with 0.05 % (*v/v*) trifluoroacetic acid; gradient elution (% A): 0–4 min: 90  %; 4–29 min: gradient from 90  % to 0  %; 29–31 min: 0  %; 31–31.5 min: gradient from 0  % to 90  %; 31.5–40 min: 90  %.

#### Exemplary procedure for the synthesis of tetracyclic CK2 PPI inhibitors

(3a*R*,4*S*,10*S*,10a*S*)‐4‐{[(*S*)‐4‐Benzyl‐2‐oxo‐1,3‐oxazolidin‐3‐yl]carbonyl}‐10‐(3,4,5‐trimethoxyphenyl)‐4,5,10,10a‐tetrahydrofuro[3,4‐*b*]carbazole‐1,3(3a*H*)‐dione ((+)‐3a)^53^ and (3a*R*,4*R*,10*S*,10a*S*)‐4‐{[(*S*)‐4‐Benzyl‐2‐oxo‐1,3‐oxazolidin‐3‐yl]carbonyl}‐10‐(3,4,5‐trimethoxyphenyl)‐4,5,10,10a‐tetrahydrofuro[3,4‐*b*]carbazole‐1,3(3a*H*)‐dione ((+)‐3c) and (3a*S*,4*S*,10*R*,10a*R*)‐4‐{[(*S*)‐4‐Benzyl‐2‐oxo‐1,3‐oxazolidin‐3‐yl]carbonyl}‐10‐(3,4,5‐trimethoxyphenyl)‐4,5,10,10a‐tetrahydrofuro[3,4‐*b*]carbazole‐1,3(3a*H*)‐dione ((+)‐3d)


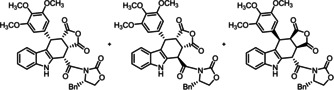


Under N_2_, indole (*S*)‐**4** (334 mg, 1.00 mmol), maleic anhydride (**6**, 292 mg, 2.98 mmol) and 3,4,5‐trimethoxybenzaldehyde (**5**, 293 mg, 1.49 mmol) were dissolved in dry toluene (10 mL) in a pressure resistant Schlenk tube. Crushed CuSO_4_ ⋅ 5 H_2_O (28.5 mg, 0.11 mmol) was added to the solution and the mixture was heated to reflux for 24 h (oil bath temperature 130 °C). After cooling to room temperature, the mixture was filtered and the filter was washed with CH_2_Cl_2_ (3×10 mL). The filtrate was concentrated in vacuo and the residue was purified by automatic flash column chromatography (cartridge: SNAP 100 g, flow rate 50 mL/min, ethyl acetate/cyclohexane=20 : 80 → 100:0). At first, a mixture of (+)‐**3** **a** and (+)‐**3** **d** then (+)‐**3** **c** was eluted.

(+)‐**3** **c** (second compound during fc): Colorless solid, mp 228 °C, yield 110 mg (18 %). ^1^H NMR (600 MHz, [D_6_]DMSO): δ (ppm)=3.01 (dd, *J*=13.6/8.4 Hz, 1H, PhC*H_2_*CH), 3.06 (dd, *J*=13.5/4.1 Hz, 1H, PhC*H_2_*CH), 3.61 (s, 3H, 4‐OCH_3_), 3.63 (s, 6H, 3‐OCH_3_, 5‐OCH_3_), 4.02 (t, *J*=9.0 Hz, 1H, 10a‐H), 4.27 (dd, *J*=8.7/3.7 Hz, 1H, OC*H_2_*CH), 4.46 (t, *J*=8.6 Hz, 1H, OC*H_2_*CH), 4.64 (dd, *J*=9.3/2.6 Hz, 1H, 3a‐H), 4.72–4.80 (m, 1H, PhCH_2_C*H*), 4.89 (d, *J*=8.5 Hz, 1H, 10‐H), 6.07 (d, *J*=2.7 Hz, 1H, 4‐H), 6.33 (s, 2H, 2‐H_TMP_, 6‐H_TMP_), 6.90 (ddd, *J*=8.0/6.9/1.0 Hz, 1H, 8‐H), 7.04–7.09 (m, 3H, 9‐H, 2‐H_benzyl_, 6‐H_benzyl_), 7.10 (ddd, *J*=8.1/6.9/1.2 Hz, 1H, 7‐H), 7.12–7.18 (m, 2H, 3‐H_benzyl_, 5‐H_benzyl_), 7.17–7.21 (m, 1H, 4‐H_benzyl_), 7.47 (dt, *J*=8.2/1.0 Hz, 1H, 6‐H), 10.82 (s, 1H, 5‐H).

For recrystallization, the previously obtained mixture of (+)‐**3** **a** and (+)‐**3** **d** was dissolved in a mixture of ethyl acetate and *tert*‐butyl methyl ether under reflux. The solution was allowed to cool down to room temperature. The formed precipitate was filtered off and washed with cold *tert*‐butyl methyl ether (2 x 10 mL) to give (+)‐**3** **d**. The filtrate was concentrated in vacuo to give (+)‐**3** **a**.

(+)‐**3** **d** (precipitate obtained after recrystallization): Colorless solid, mp 229 °C, yield 15 mg (2 %). ^1^H NMR (600 MHz, CDCl_3_): δ (ppm)=2.84 (dd, *J*=13.4/9.6 Hz, 1H, PhC*H_2_*CH), 3.36 (dd, *J*=13.4/3.5 Hz, 1H (PhC*H_2_*CH), 3.74 (s, 6H, 3‐OCH_3_, 5‐OCH_3_), 3.88 (s, 3H, 4‐OCH_3_), 3.97 (dd, *J*=8.9/1.8 Hz, 1H, 3a‐H), 4.21–4.34 (m, 3H, 10a‐H, OC*H_2_*CH), 4.55–4.63 (m, 1H, PhCH_2_C*H*), 4.69 (d, *J*=9.9 Hz, 1H, 10‐H), 6.02 (d, *J*=1.8 Hz, 1H, 4‐H), 6.39 (s, 2H, 2‐H_TMP_, 6‐H_TMP_), 6.66 (d, *J*=8.1 Hz, 1H, 9‐H), 6.87 (ddd, *J*=8.1/7.0/1.0 Hz, 1H, 8‐H), 7.14 (ddd, *J*=8.2/7.0/1.2 Hz, 1H, 7‐H), 7.19–7.23 (m, 2H, 2‐H_benzyl_, 6‐H_benzyl_), 7.28–7.42 (m, 4H, 6‐H, 3‐H_benzyl_, 4‐H_benzyl_, 5‐H_benzyl_), 8.85 (s, 1H, 5‐H).

(+)‐**3** **a** (compound isolated from the filtrate after recrystallization): Colorless solid, mp 187 °C, yield 170 mg (28  %). ^1^H NMR (600 MHz, CDCl_3_): δ (ppm)=2.76 (dd, *J*=13.8/11.0 Hz, 1H, PhC*H_2_*CH), 3.65 (dd, *J*=13.7/3.1 Hz, 1H, PhC*H_2_*CH), 3.74 (s, 6H, 3‐OCH_3_, 5‐OCH_3_), 3.75 (s, 3H, 4‐OCH_3_), 3.89 (dd, *J*=9.2/7.9 Hz, 1H, 10a‐H), 4.20–4.29 (m, 2H, OC*H_2_*CH), 4.56 (dd, *J*=10.2/9.1 Hz, 1H, 3a‐H), 4.69–4.74 (m, 1H,OCH_2_C*H*), 4.86 (d, *J*=7.9 Hz, 1H, 10‐H), 5.39 (d, *J*=10.1 Hz, 1H, 4‐H), 6.53 (s, 2H, 2‐H_TMP_, 6‐H_TMP_), 7.03 (ddd, *J*=7.9/7.0/0.9 Hz, 1H, 8‐H), 7.16–7.22 (m, 3H, 7‐H, 2‐H_benzyl_, 6‐H_benzyl_), 7.25–7.39 (m, 5H, 5‐H, 8‐H, 3‐H_benzyl_, 4‐H_benzyl_, 5‐H_benzyl_), 8.25 (s, 1H, 5‐H).

3a*R*,4*R*,10*R*,10a*R*)‐4‐{[(*R*)‐4‐Benzyl‐2‐oxo‐1,3‐oxazolidin‐3‐yl]carbonyl}‐10‐(3,4,5‐trimethoxyphenyl)‐4,5,10,10a‐tetrahydropyrrolo[3,4‐*b*]carbazole‐1,3(2*H*,3a*H*)‐dione ((‐)‐9a) and (3a*S*,4*S*,10*S*,10a*S*)‐4‐{[(*R*)‐4‐Benzyl‐2‐oxo‐1,3‐oxazolidin‐3‐yl]carbonyl}‐10‐(3,4,5‐trimethoxyphenyl)‐4,5,10,10a‐tetrahydropyrrolo[3,4‐*b*]carbazole‐1,3(2*H*,3a*H*)‐dione ((+)‐9b)


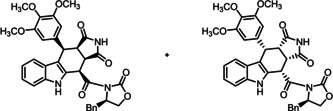


Under N_2_, indole (*R*)‐**4** (168 mg, 0.50 mmol), maleimide (**7**, 146 mg, 1.50 mmol) and 3,4,5‐trimethoxybenzaldehyde (**5**, 147 mg, 0.75 mmol) were dissolved in dry toluene (10 mL) in a pressure resistant Schlenk tube. Crushed CuSO_4_ ⋅ 5 H_2_O (12.6 mg, 0.05 mmol) was added to the solution and the mixture was heated to reflux for 22 h (oil bath temperature 130 °C). After cooling to room temperature, the mixture was filtered and the filter was washed with CH_2_Cl_2_ (3×10 mL). The filtrate was concentrated in vacuo and the residue was purified by flash column chromatography (ethyl acetate/cyclohexane=45 : 55, Ø 3 cm, *h*=18 cm, *V*=20 mL). The first obtained fraction was again purified by flash column chromatography (ethyl acetate/CH_2_Cl_2_=2 : 8, Ø 4 cm, *h*=18 cm, *V*=30 mL) to give (−)‐**9** **a**. The second obtained fraction was also purified once more by flash column chromatography (ethyl acetate/CH_2_Cl_2_=3 : 7, Ø 4 cm, *h*=19 cm, *V*=30 mL) to give (+)‐**9** **b**.

(−)‐**9** **a** [*R*_f_=0.41 (ethyl acetate/CH_2_Cl_2_=2 : 8)]: Yellow solid, mp 216 °C, yield 107 mg (35 %). ^1^H NMR (600 MHz, [D_6_]DMSO): δ (ppm)=2.85 (dd, *J*=13.6/10.1 Hz, 1H, PhC*H_2_*CH), 3.46 (dd, *J*=13.6/2.9 Hz, 1H, PhC*H_2_*CH), 3.55 (s, 3H, 4‐OCH_3_), 3.58 (t, *J*=8.3 Hz, 1H, 10a‐H), 3.67 (s, 6H, 3‐OCH_3_, 5‐OCH_3_), 4.31 (dd, *J*=9.0/2.2 Hz, 1H, C*H*_*2*oxazolidine_), 4.35 (dd, *J*=11.6/8.6 Hz, 1H, 3a‐H), 4.42 (t, *J*=8.4 Hz, 1H, C*H*_*2*oxazolidine_), 4.65–4.72 (m, 1H, C*H*_oxazolidine_), 4.72 (d, *J*=7.6 Hz, 1H, 10‐H), 5.35 (d, *J*=11.6 Hz, 1H, 4‐H), 6.72 (s, 2H, 2‐H_TMP_, 6‐H_TMP_), 6.91 (ddd, *J*=8.0/6.9/0.7 Hz, 1H, 8‐H), 7.05 (ddd, *J*=8.2/7.0/1.1 Hz, 1H, 7‐H), 7.25–7.29 (m, 1H, 4‐H_benzyl_), 7.31–7.38 (m, 6H, 6‐H, 9‐H, 2‐H_benzyl_, 3‐H_benzyl_, 5‐H_benzyl_, 6‐H_benzyl_), 10.77 (s, 1H, 2‐H), 10.81 (s, 1H, 5‐H).

(+)‐**9** **b** [*R*_f_=0.32 (ethyl acetate/CH_2_Cl_2_=2 : 8)]: Yellow solid, mp 225 °C, yield 70 mg (23 %). ^1^H NMR (600 MHz, [D_6_]DMSO): δ (ppm)=2.99 (dd, *J*=13.5/8.7 Hz, 1H, PhC*H_2_*CH), 3.54 (dd, *J*=8.9/8.0 Hz, 1H, 10a‐H), 3.56 (s, 3H, 4‐OCH_3_), 3.66 (s, 6H, 3‐OCH_3_, 5‐OCH_3_), 4.23 (dd, *J*=11.6/8.5 Hz, 1H, 3a‐H), 4.30 (dd, *J*=9.0/3.7 Hz, 1H, C*H*_*2*oxazolidine_), 4.39 (t, *J*=8.7 Hz, 1H, C*H*_*2*oxazolidine_), 4.66–4.70 (m, 1H, C*H*_oxazolidine_), 4.71 (d, *J*=7.5 Hz, 1H, 10‐H), 5.48 (d, *J*=11.6 Hz, 1H, 4‐H), 6.62 (s, 2H, 2‐H_TMP_, 6‐H_TMP_), 6.91 (ddd, *J*=7.9/7.0/1.0 Hz, 1H, 8‐H), 7.07 (ddd, *J*=8.1/7.0/1.2 Hz, 1H, 7‐H), 7.27–7.32 (m, 1H, 4‐H_benzyl_), 7.31–7.40 (m, 6H, 6‐H, 9‐H, 2‐H_benzyl_, 3‐H_benzyl_, 5‐H_benzyl_, 6‐H_benzyl_), 10.75 (s, 1H, 2‐H), 11.02 (s, 1H, 5‐H). Signal for the second PhC*H_2_*CH proton is overlaid by the H_2_O signal at 3.30 ppm.

(3a*R*,4*R*,10*R*,10a*R*)‐4‐{[(*R*)‐4‐Benzyl‐2‐oxo‐1,3‐oxazolidin‐3‐yl]carbonyl}‐2‐methyl‐10‐(3,4,5‐trimethoxyphenyl)‐4,5,10,10a‐tetrahydropyrrolo[3,4‐*b*]carbazole‐1,3(2*H*,3a*H*)‐dione ((‐)‐10a) and (3a*S*,4*S*,10*S*,10a*S*)‐4‐{[(*R*)‐4‐Benzyl‐2‐oxo‐1,3‐oxazolidin‐3‐yl]carbonyl}‐2‐methyl‐10‐(3,4,5‐trimethoxyphenyl)‐4,5,10,10a‐tetrahydropyrrolo[3,4‐*b*]carbazole‐1,3(2*H*,3a*H*)‐dione ((+)‐10b)


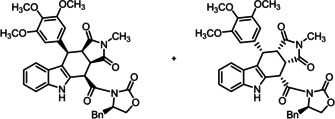


Under N_2_, indole (*R*)‐**4** (167 mg, 0.50 mmol), *N*‐methylmaleimide (**8**, 166 mg, 1.49 mmol) and 3,4,5‐trimethoxybenzaldehyde (**5**, 146 mg, 0.75 mmol) were dissolved in dry toluene (15 mL) in a pressure resistant Schlenk tube. Crushed CuSO_4_ ⋅ 5 H_2_O (12.9 mg, 0.05 mmol) was added to the solution and the mixture was heated to reflux for 16 h (oil bath temperature 130 °C). After cooling to room temperature, the mixture was filtered and the filter was washed with CH_2_Cl_2_ (3 x 10 mL). The filtrate was concentrated in vacuo and the residue was purified by flash column chromatography (ethyl acetate/cyclohexane=3 : 7→ 1 : 1, Ø 3 cm, *h*=20 cm, *V*=20 mL). After repeated purification by flash column chromatography (ethyl acetate/CH_2_Cl_2_=10 : 90→ 15 : 85, Ø 4 cm, *h*=20 cm, *V*=30 mL), the isomers (−)‐**10** **a** and (+)‐**10** **b** were isolated.

(−)‐**10** **a** [*R*_f_=0.22 (ethyl acetate/CH_2_Cl_2_ 1 : 9)]: Yellow solid, mp 238 °C, yield 82 mg (26 %). ^1^H NMR (600 MHz, [D_6_]DMSO): δ (ppm)=2.22 (s, 3H, NHC*H_3_*), 2.86 (dd, *J*=13.6/10.3 Hz, 1H, PhC*H_2_*CH), 3.51 (dd, *J*=13.6/2.9 Hz, 1H, PhC*H_2_*CH), 3.52 (s, 3H, 4‐OCH_3_), 3.65 (t, *J*=7.8 Hz, 1H, 10a‐H), 3.67 (s, 6H, 3‐OCH_3_, 5‐OCH_3_), 4.32 (dd, *J*=9.0/2.2 Hz, 1H, C*H*_*2*oxazolidine_), 4.36 (dd, *J*=11.5/7.8 Hz, 1H, 3a‐H), 4.43 (t, *J*=8.5 Hz, 1H, C*H*_*2*oxazolidine_), 4.69 (tt, *J*=7.7/2.6 Hz, 1H, C*H*_oxazolidine_), 4.72 (d, *J*=7.6 Hz, 1H, 10‐H), 5.38 (d, *J*=11.5 Hz, 1H, 4‐H), 6.63 (s, 2H, 2‐H_TMP_, 6‐H_TMP_), 6.91 (ddd, *J*=8.0/7.0/0.9 Hz, 1H, 8‐H), 7.06 (ddd, *J*=8.1/7.0/1.2 Hz, 1H, 7‐H), 7.25–7.31 (m, 1H, 4‐H_benzyl_), 7.31–7.39 (m, 6H, 6‐H, 9‐H, 2‐H_benzyl_, 3‐H_benzyl_, 5‐H_benzyl_, 6‐H_benzyl_), 10.81 (s, 1H, 5‐H).

(+)‐**10** **b** [*R*_f_=0.17 (ethyl acetate/CH_2_Cl_2_ 1 : 9)]: Yellow solid, mp 216 °C, yield 75 mg (24 %). ^1^H NMR (400 MHz, [D_6_]DMSO): δ (ppm)=2.19 (s, 3H, NHC*H_3_*), 3.00 (dd, *J*=13.5/8.8 Hz, 1H, PhC*H_2_*CH), 3.37 (dd, *J*=13.5/3.2 Hz, 1H, PhC*H_2_*CH), 3.53 (s, 3H, 4‐OCH_3_), 3.63 (t, *J*=7.7 Hz, 1H, 10a‐H), 3.66 (s, 6H, 3‐OCH_3_, 5‐OCH_3_), 4.24 (dd, *J*=11.5/8.0 Hz, 1H, 3a‐H), 4.31 (dd, *J*=9.0/3.8 Hz, 1H, C*H*_*2*oxazolidine_), 4.42 (t, *J*=8.7 Hz, 1H, C*H*_*2*oxazolidine_), 4.72 (d, *J*=7.3 Hz, 1H, 10‐H), 4.70–4.77 (m, 1H, C*H*_oxazolidine_), 5.51 (d, *J*=11.5 Hz, 1H, 4‐H), 6.54 (s, 2H, 2‐H_TMP_, 6‐H_TMP_), 6.92 (ddd, *J*=8.0/7.0/1.0 Hz, 1H, 8‐H), 7.08 (ddd, *J*=8.2/7.0/1.2 Hz, 1H, 7‐H), 7.25–7.43 (m, 7H, 6‐H, 9‐H, 2‐H_benzyl_, 3‐H_benzyl_, 4‐H_benzyl_, 5‐H_benzyl_, 6‐H_benzyl_), 11.04 (s, 1H, 5‐H).

### Microscale thermophoresis

The proteins CK2α^1−335^ and CK2β^1−193^ were recombinantly expressed and purified as described in literature,^50^, ^60^ with the exception of the first purification step on phosphocellulose, where the protein was eluted using high salt buffer (1 M NaCl, 25 mM Tris/HCl, pH 8.5) without applying a gradient. CK2β^1−193^ was fluorescently labelled using the Nanotemper Monolitz^TM^ NT.115 Protein Labeling Kit RED‐NHS according to the manufacturer's manual. To quantify the protein‐protein interaction in the presence of potential inhibitors, the investigated compounds were first dissolved in DMSO (2‐10 mM) and then 50‐fold diluted in ITC buffer (25 mM Tris‐HCl, 500 mM NaCl, pH 8.5) containing 0.1 % (*v*/*v*) Tween 20 to concentrations of either 40, 100 or 200 μM in 2 % (*v*/*v*) DMSO, i. e. twice the final concentrations: 20, 50 and 100 μM, respectively, in 1 % (*v*/*v*) DMSO, 0.05 % (*v*/*v*) Tween 20. Fluorescently labeled CK2β^1−193^ in ITC buffer was added to the mixture to a concentration of 40 nM, followed by a centrifugation step to remove aggregates. A volume of 10 μl of this mixture was then added to the same volume of CK2α^1−335^ in ITC buffer (16 serial dilutions between 0.305 and 10000 nM) to obtain final concentrations of fluorescently labelled CK2β^1−193^ and CK2α^1−335^ of 20 nM and 0.1526 ‐ 5000 nM, respectively. MST traces were recorded at room temperature using a Nanotemper Monolith™ NT.115 Series Instrument with Monolith™ NT.115 standard treated capillaries and normalized to initial fluorescence (MO.Affinity Analysis, Nanotemper). The change in normalized fluorescence (Δ*F*_norm_) was plotted against the CK2α^1−335^ concentration and analyzed^61^ resulting in dissociation constants *K*_D_ and *K*_D_’ for the CK2α^1−335^/CK2β^1−193^ interaction in the absence and presence of potential inhibitors, respectively (Table S4). Next, the statistical significance of the shift from *K*_D_ to *K*_D_’ was determined for each compound by means of an unpaired Student's t test (Table S4). For compounds showing a statistically significant difference between *K*_D_’ and *K*_D_, the MST experiments in the presence and absence of compound (altogether 42 experiments) were re‐analyzed by setting *K*_D_ to a global value and additionally applying the equation *K*_D_‘=*K*_D_ [1+(c_inhibitor_/*K*_i_)].^57^ This calculation resulted in a global value of *K*_D =_ 11 nM, that is, the same value as previously found^57^ by applying the same methodology, and the *K*_i_ values shown in the fifth column of Table 1. Data was analyzed and statistically evaluated with the program GraphPad Prism v.5.04 for Windows (GraphPad Software, San Diego, CA, USA).

### Capillary electrophoresis assay to determine the enzyme inhibition

Enzymatic activities with or without inhibitors were determined for the holoenzyme (CK2α_2_β_2_), the CK2α subunit, and the mutated CK2α′ ^C336S^ subunit by a capillary electrophoresis assay as described before.^59^ For this purpose, CK2α as well as (CK2α_2_β_2_) holoenzyme were purified after recombinant expression in *E. coli* BL21(DE3) and purified according the protocol of Grankowski et al.^62^ The mutated CK2α′ ^C336S^ subunit was purified by Ni‐NTA affinity chromatography using an N‐terminally attached His_6_ tag. Successful purification was controlled by SDS‐PAGE. Enzymatic activity was determined in the presence of 60 μM ATP and 114 μM of the substrate peptide RRRDDDSDDD. For both CK2α subunits an assay buffer containing 100 mM NaCl instead of 60 mM NaCl as for the holoenzyme, and 20 mM MgCl_2_ instead of 10 mM MgCl_2_ was applied. For the holoenzyme, 1 μg was added, whereas for CK2α and the mutated CK2α′ ^C336S^ subunit 0.25 μg was added each. For each compound inhibition was determined three times independently at an initial concentration of 10 μM and the mean value and the standard deviation (SD) were calculated. For compounds showing more than 60 % inhibition at a concentration of 10 μM with respect to the enzyme without inhibitor, but the same amount of DMSO used for solving, an IC_50_ value was determined again in three independent experiments.

## Supporting Information

Characteristic NMR data, a summary of the specific optical rotation, determination of enantiomeric purity by chiral HPLC, synthesis of (*S*)‐**4** and (*R*)‐**4** and the X‐ray crystal structure analysis of (+)‐**3** **d**, (+)‐**10** **b** and (+)‐**14** **d**. CCDC‐1951235, CCDC‐1951236 and CCDC‐1951237 contain the supplementary crystallographic data for these compounds. These data can be obtained free of charge from The Cambridge Crystallographic Data Centre via www.ccdc.cam.ac.uk/structures. In addition, dissociation constants obtained by MST for the CK2α^1−335^/CK2β^1−193^ interaction in the absence and presence of test compounds are provided. Finally, all ^1^H and ^13^C NMR spectra of the compounds are displayed.

## Conflict of interest

The authors declare no conflict of interest.

## Supporting information

As a service to our authors and readers, this journal provides supporting information supplied by the authors. Such materials are peer reviewed and may be re‐organized for online delivery, but are not copy‐edited or typeset. Technical support issues arising from supporting information (other than missing files) should be addressed to the authors.

Supplementary
