# Supplementary material for: Synthesis and SAR of Tetracyclic Inhibitors of Protein Kinase CK2 Derived from Furocarbazole W16
Source: ChemMedChem. 2020 Apr 27;15(10):871–81. doi: 10.1002/cmdc.202000040 (PMC7418559; doi:10.1002/cmdc.202000040)

ChemMedChem

Supporting Information

Synthesis and SAR of Tetracyclic Inhibitors of Protein Kinase CK2 Derived from Furocarbazole W16

Lukas Kröger, Constantin G. Daniliuc, Deeba Ensan, Sebastian Borgert, Christian Nienberg, Miriam Lauwers, Michaela Steinkrüger, Joachim Jose, Markus Pietsch, and Bernhard Wünsch*

Table of contents page

1. Characteristic NMR data S2
2. Summary of the specific optical rotation S3
3. Determination of enantiomeric purity by chiral HPLC S4
4. Synthesis of (*S*)-**4** and (*R*)-**4** S5
5. Procedures for the synthesis of tetracyclic CK2 PPI inhibitors S9
6. X-Ray crystallography S26
7. Inhibition of the CK2α/CK2β interaction analyzed by MST S30
8. References S31
9. ^{1}H and ^{13}C NMR spectra S32

1. Characteristic NMR data

Table 1: Chemical shifts and coupling constants of the protons of ring B.

		4-H	3a-H	10a-H	10-H
(+)-**3a**	δ [ppm]	5,39	4,56	3,89	4,86
	multiplicity	d	dd	dd	d
	J [Hz]	10,1	10,2/9,1	9,2/7,9	7,9
(+)-**3c**	δ [ppm]	6,07	4,64	4,02	4,89
	multiplicity	d	dd	t	d
	J [Hz]	2,7	9,3/2,6	9,0	8,5
(+)-**3d**	δ [ppm]	6,02	3,9	4,21 -4,34	4,69
	multiplicity	d	dd	m	d
	J [Hz]	1,8	8,9/1,8	-	9,9

2. Summary of the specific optical rotation

Table S2: Specific optical rotation of **9a**,**b** and **10a**,**b** used for the determination of the absolute configuration of *cis*,*cis*,*cis*-configured pyrrolocarbazoles.

compd.	$[\alpha]_D^{20}$	configuration of ring B	configuration C-4 of oxazolidine
(+)-**9a**	+78	3a*S*,4*S*,10*S*,10a*S*	*S*
(-)-**9a**	-80	3a*R*,4*R*,10*R*,10a*R*	*R*
(+)-**9b**	+62	3a*S*,4*S*,10*S*,10a*S*	*R*
(-)-**9b**	-59	3a*R*,4*R*,10*R*,10a*R*	*S*
(+)-**10a**	+89	3a*S*,4*S*,10*S*,10a*S*	*S*
(-)-**10a**	-78	3a*R*,4*R*,10*R*,10a*R*	*R*
(+)-**10b**	+82	3a*S*,4*S*,10*S*,10a*S*	*R*
(-)-**10b**	-78	3a*R*,4*R*,10*R*,10a*R*	*S*

3. Determination of enantiomeric purity by chiral HPLC

Equipment: Pump: L-6200A Intelligent Pump, injector: Rheodyne 7725i, DAD detector: L-7455, interface: D-7000, data acquisition: HSM-Software (all from LaChrom, Merck Hitachi). Column: Daicel Chiralpak® IA, 5 µm, 250 mm / 4.6 mm. Guard column: Daicel Chiralpak® IA 5 µm, 10 mm / 4 mm. Solvent: *i*-hexane/ethanol 70:30, isocratic elution. Flow rate: 1.0 mL/min. Injection volume: 5 µL. Detection wavelength: 210 nm. Calculation: manual integration. Calculation method: area %, use of blank subtraction from the same series.

Table S3: Enantiomeric purity of pyrrolocarbazoles (+)-**9c**, (-)-**9c**, (+)-**9d**, and (-)-**9c**.

	(+)-**9c**	(-)-**9c**	(+)-**9d**	(-)-**9d**
ee (%)	97.1	99.8	99.6	92.1

4. Synthesis of (*S*)-4 and (R)-4

Procedures for the synthesis of **15**, **11**, and (*S*)-**4** are already described in literature. Here, modified syntheses and additional spectroscopic data are given.

2,2-Dimethyl-5-[2-(2-nitrophenyl)acetyl]-1,3-dioxane-4,6-dione (15)[1]

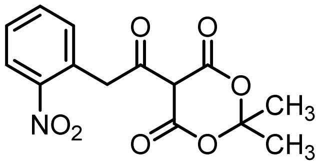

Under N_2, (2-nitrophenyl)acetic acid (12.5 g, 69.0 mmol) was suspended in dry CH_2Cl_2 (150 mL). Oxalyl chloride (11.1 g, 87.4 mmol) and a catalytic amount of DMF (5 drops) were added and the mixture was stirred at room temperature until no further gas production was observed (4 h). The mixture was then concentrated in vacuo and the residue was dissolved in dry CH_2Cl_2 (20 mL). The resulting mixture was added dropwise to a stirred solution of Meldrum's acid (9.94 g, 0.69 mmol) and DIPEA (19.6 g, 152 mmol) in dry CH_2Cl_2 (150 mL) at 0 °C. The mixture was allowed to warm to room temperature and stirring was continued for 2 h. After completion of the transformation, HCl solution (1 M in water, 200 mL) was added and the mixture was stirred for 30 min. The layers were separated and the aqueous layer was extracted with CH_2Cl_2 (3 x 75 mL). The combined organic layers were dried (Na_2SO_4), filtered and concentrated in vacuo. The residue was suspended in EtOH (50 mL) and the mixture was put into an ultrasonic bath for 30 min. The slurry was cooled down (-20 °C), filtered and the solid was washed with cold EtOH (3 x 30 mL) and cold Et_2O (4 x 30 mL). Colorless solid, mp 113 °C, yield 16.9 g (80 %). $C_{14}H_{13}NO_7$ (307.3). TLC (CH_2Cl_2/MeOH = 8:2): R_f = 0.32. Purity (HPLC): Decomposition during standard procedure. Due to decomposition of the product in DMSO-d_6, signals of 1-(2-nitrophenyl)propan-2-one, acetone and CO_2 are observed in the NMR spectra. Exact mass (ESI): m/z = 308.0747 (calcd. 308.0765 for $C_{14}H_{14}NO_7$ $[MH]^+$). IR (neat): ṽ [cm^{-1}] = 2978, 2886 (m, C-$H_{aliph.}$), 1735 (s, C=O), 1192 (s, C-O), 725 (s, C-$H_{arom.}$).

Ethyl 2-(indol-2-yl)acetate (11)[2]

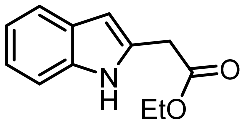

Under N_2, acylated Meldrum's acid **15** (7.80 g, 25.4 mmol) was suspended in EtOH (100 mL). The mixture was heated to reflux for 5 h. After completion of the transformation, the resulting solution was concentrated in vacuo. The residue was dissolved in a biphasic mixture of THF (100 mL) and saturated NH_4Cl solution (100 mL). Under vigorous stirring, Zn dust (16.6 g, 254 mmol) was added and the suspension was heated to 60 °C for 12 h. Saturated $NaHCO_3$ solution (20 mL) was added and the mixture was filtered through Celite®, which was then rinsed with ethyl acetate (100 mL). The layers of the filtrate were separated and the aqueous layer was extracted with ethyl acetate (100 mL). The combined organic layers were dried (Na_2SO_4), filtered and concentrated in vacuo. The residue was purified by flash column chromatography (CH_2Cl_2, Ø 8 cm, h = 15 cm, v = 65 mL). brown solid, mp 35 °C, yield 4.56 g (88 %). $C_{12}H_{13}NO_2$ (203.2). TLC (CH_2Cl_2): R_f = 0.54. Purity (HPLC): 97.3 %, (t_R = 19.5 min). ^{1}H NMR (600 MHz, DMSO-d_6): δ (ppm) = 1.21 (t, J = 7.1 Hz, 3H, $OCH_2C\mathit{H_3}$), 3.82 (s, 2H, $ArC\mathit{H_2}CO$), 4.12 (q, J = 7.1 Hz, 2H, $OC\mathit{H_2}CH_3$), 6.24 – 6.28 (m, 1H, 3-H_{indole}), 6.94 (ddd, J = 8.1/7.0/1.1 Hz, 1H, 5-H_{indole}), 7.02 (ddd, J = 8.1/7.0/1.2 Hz, 1H, 6-H_{indole}), 7.29 – 7.34 (m, 1H, 7-H_{indole}), 7.41 – 7.46 (m, 1H, 4-H_{indole}), 11.01 (s, 1H, 1-H_{indole}). ^{13}C NMR (151 MHz, DMSO-d_6): δ (ppm) = 14.1 (1C, $OCH_2\mathit{C}H_3$), 33.7 (1C, $Ar\mathit{C}H_2CO$), 60.5 (1C, $O\mathit{C}H_2CH_3$), 100.6 (1C, C-3_{indole}), 110.9 (1C, C-7_{indole}), 118.8 (1C, C-5_{indole}), 119.4 (1C, C-4_{indole}), 120.6 (1C, C-6_{indole}), 128.0 (1C, C-$3a_{indole}$), 131.9 (1C, C-2_{indole}), 136.1 (1C, C-$7a_{indole}$), 169.9 (1C, $ArCH_2\mathit{C}O$). Exact mass (ESI): m/z = 204.1019 (calcd. 204.1019 for $C_{12}H_{14}NO_2$ $[MH]^+$). IR (neat): ṽ [cm^{-1}] = 2978 (w, C-$H_{aliph.}$), 1720 (s, C=O), 1150 (s, C-O), 745 (m, C-$H_{arom.}$).

(*S*)-3-[2-(Indol-2-yl)acetyl]-4-benzyl-1,3-oxazolidin-2-one ((*S*)-4)[1]

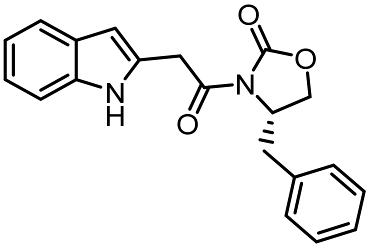

Under N_2, acylated Meldrum's acid **15** (3.38 g, 11.0 mmol) and (*S*)-4-benzyl-1,3-oxazolidin-2-one (1.77 g, 9.99 mmol) were dissolved in CH_3CN (30 mL). The mixture was heated to reflux for 3 h. After completion of the transformation, the resulting solution was concentrated in vacuo. The residue was dissolved in a biphasic mixture of THF (50 mL) and saturated NH_4Cl solution (50 mL). Under vigorous stirring, Zn dust (3.60 g, 55.1 mmol) was added and the suspension was heated to 60 °C for 16 h. Saturated $NaHCO_3$ solution (100 mL) was added and the mixture was filtered through Celite®, which was then rinsed with ethyl acetate (140 mL). The layers of the filtrate were separated and the aqueous layer was extracted with ethyl acetate (2 x 50 mL). The combined organic layers were dried (Na_2SO_4), filtered and concentrated in vacuo. The residue was purified by flash column chromatography (CH_2Cl_2/hexane = 8:2, Ø 4 cm, h = 14 cm, v = 30 mL). Yellow solid, mp 121 °C, yield 1.71 g (51 %). $C_{20}H_{18}N_2O_3$ (334.3). TLC (CH_2Cl_2/hexane = 8:2): R_f = 0.21. Purity (HPLC): 99.8 %, (t_R = 21.5 min). Specific rotation: $[\alpha]_D^{20}$ = +98 (c = 5.7, $CHCl_3$). ^1H NMR (600 MHz, DMSO-d_6): δ (ppm) = 2.95 (dd, J = 13.6/7.4 Hz, 1H, PhCH_2CH), 3.02 (dd, J = 13.5/3.2 Hz, 1H, PhCH_2CH), 4.22 (dd, J = 8.9/2.8 Hz, 1H, OCH_2CH), 4.31 (d, J = 16.8 Hz, 1H, ArCH_2CO), 4.37 (t, J = 8.6 Hz, 1H, OCH_2CH), 4.43 (d, J = 16.7 Hz, 1H, ArCH_2CO), 4.67 – 4.74 (m, 1H, PhCH$_2$C*H*), 6.30 (d, J = 1.1 Hz, 1H, 3-H_{indole}), 6.96 (ddd, J = 8.0/6.9/1.0 Hz, 1H, 5-H_{indole}), 7.04 (ddd, J = 8.2/7.0/1.2 Hz, 1H, 6-H_{indole}), 7.14 – 7.20 (m, 2H, 2-H_{benzyl}, 6-H_{benzyl}), 7.20 – 7.28 (m, 3H, 3-H_{benzyl}, 4-H_{benzyl}, 5-H_{benzyl}), 7.35 (d, J = 8.0 Hz, 1H, 7-H_{indole}), 7.47 (dd, J = 7.8/1.1 Hz, 1H, 4-H_{indole}), 11.00 (s, 1H, 1-H_{indole}). ^{13}C NMR (151 MHz, DMSO-d_6): δ (ppm) = 34.5 (1C, O*C*H$_2$CH), 36.5 (1C, Ph*C*H$_2$CH), 54.4 (1C, PhCH$_2$*C*H), 66.1 (1C, Ar*C*H$_2$CO), 100.9 (1C, C-3$_{indole}$), 110.9 (1C, C-7$_{indole}$), 118.7 (1C, C-5$_{indole}$), 119.4 (1C, C-4$_{indole}$), 120.6 (1C, C-6$_{indole}$), 126.9 (1C, C-4$_{benzyl}$), 128.0 (1C, C-3a$_{indole}$), 128.5 (2C, C-3$_{benzyl}$, C-5$_{benzyl}$), 129.5 (2C, C-2-$_{benzyl}$, C-6$_{benzyl}$), 131.6 (1C, C-2$_{indole}$), 135.5 (1C, C-1$_{benzyl}$), 136.2 (1C, C-7a$_{indole}$), 153.4 (1C, CO$_{oxazolidine}$), 169.2 (1C, ArCH$_2$*C*O). Exact mass (ESI): m/z = 335.1378 (calcd.

335.1390 for $C_{20}H_{19}N_2O_3$ [MH]$^+$). IR (neat): ṽ [cm^{-1}] = 2978 (w, C-$H_{aliph.}$), 1763, 1697 (s, C=O), 1219, 1184 (m, C-O), 752, 706 (m, C-$H_{arom.}$).

(*R*)-3-[2-(Indol-2-yl)acetyl]-4-benzyl-1,3-oxazolidin-2-one ((*R*)-4)

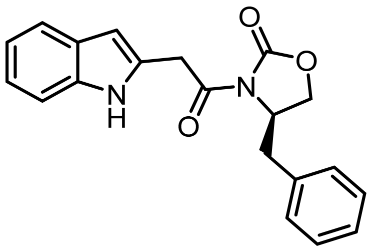

As described for the synthesis of (*S*)-**4**, acylated Meldrum's acid **15** (942 mg, 3.07 mmol), (*R*)-4-benzyl-1,3-oxazolidin-2-one (436 mg, 2.46 mmol) and Zn dust (10.0 g, 153 mmol) were transformed. Work-up and purification were performed as described above. Contrary to the synthesis of the enantiomer (*S*)-**4**, the reaction was carried out in dry THF and the β-ketoester obtained in the first step was purified by flash column chromatography (CH_2Cl_2/MeOH = 9:1, Ø 3 cm, h = 19 cm, v = 20 mL) prior to further reaction. Yellow solid, mp 121 °C, yield 338 mg (41 %). $C_{20}H_{18}N_2O_3$ (334.3). TLC (CH_2Cl_2/hexane = 8:2): R_f = 0.21. Purity (HPLC): 99.6 %, (t_R = 21.5 min). Specific rotation: $[\alpha]_D^{20}$ = -99 (c = 2.7, $CHCl_3$) Exact mass (ESI): m/z = 385.1379 (calcd. 335.1390 for $C_{20}H_{19}N_2O_3$ [MH]$^+$).

5. Procedures for the synthesis of tetracyclic CK2 PPI inhibitors

(3a*R*,4*S*,10*S*,10a*S*)-4-{[(*S*)-4-Benzyl-2-oxo-1,3-oxazolidin-3-yl]carbonyl}-10-(3,4,5-trimethoxyphenyl)-4,5,10,10a-tetrahydrofuro[3,4-*b*]carbazole-1,3(3a*H*)-dione ((+)-3a)[44] and
(3a*R*,4*R*,10*S*,10a*S*)-4-{[(*S*)-4-Benzyl-2-oxo-1,3-oxazolidin-3-yl]carbonyl}-10-(3,4,5-trimethoxyphenyl)-4,5,10,10a-tetrahydrofuro[3,4-*b*]carbazole-1,3(3a*H*)-dione ((+)-3c) and
(3a*S*,4*S*,10*R*,10a*R*)-4-{[(*S*)-4-Benzyl-2-oxo-1,3-oxazolidin-3-yl]carbonyl}-10-(3,4,5-trimethoxyphenyl)-4,5,10,10a-tetrahydrofuro[3,4-*b*]carbazole-1,3(3a*H*)-dione ((+)-3d)

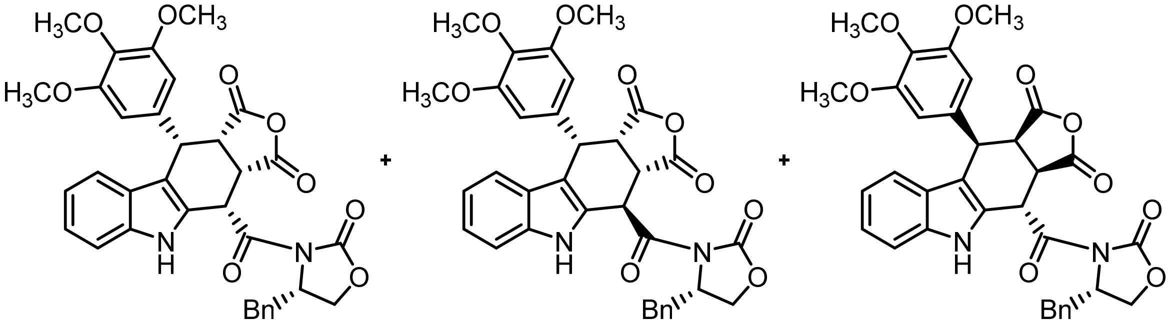

Under N_2, indole (*S*)-**4** (334 mg, 1.00 mmol), maleic anhydride (**6**, 292 mg, 2.98 mmol) and 3,4,5-trimethoxybenzaldehyde (**5**, 293 mg, 1.49 mmol) were dissolved in dry toluene (10 mL) in a pressure resistant Schlenk tube. Crushed $CuSO_4 \cdot 5\ H_2O$ (28.5 mg, 0.11 mmol) was added to the solution and the mixture was heated to reflux for 24 h (oil bath temperature 130 °C). After cooling to room temperature, the mixture was filtered and the filter was washed with CH_2Cl_2 (3 x 10 mL). The filtrate was concentrated in vacuo and the residue was purified by automatic flash column chromatography (cartridge: SNAP 100g, flow rate 50 mL/min, ethyl acetate/cyclohexane = 20:80 → 100:0). At first, a mixture of (+)-**3a** and (+)-**3d** then (+)-**3c** was eluted.

(+)-**3c** (second compound during fc): Colorless solid, mp 228 °C, yield 110 mg (18 %). $C_{34}H_{30}N_2O_9$ (610.6). TLC: Decomposition during TLC. Purity (HPLC): 92.5 %, (t_R = 22.9 min). Specific rotation: $[\alpha]_D^{20}$ = +94 (c = 1.3, $CHCl_3$). ^1H NMR (600 MHz, DMSO-*d*6): δ (ppm) = 3.01 (dd, J = 13.6/8.4 Hz, 1H, PhC*H*2CH), 3.06 (dd, J = 13.5/4.1 Hz, 1H, PhC*H*2CH), 3.61 (s, 3H, 4-OCH_3), 3.63 (s, 6H, 3-OCH_3, 5-OCH_3), 4.02 (t, J = 9.0 Hz, 1H, 10a-H), 4.27 (dd, J = 8.7/3.7 Hz, 1H, OC*H*2CH), 4.46 (t, J = 8.6 Hz, 1H, OC*H*2CH), 4.64 (dd, J = 9.3/2.6 Hz, 1H, 3a-H), 4.72 – 4.80 (m, 1H, PhCH2C*H*), 4.89 (d, J = 8.5 Hz, 1H, 10-H), 6.07 (d, J = 2.7 Hz, 1H, 4-H), 6.33 (s, 2H, 2-H_{TMP}, 6-H_{TMP}), 6.90 (ddd, J

= 8.0/6.9/1.0 Hz, 1H, 8-H), 7.04 – 7.09 (m, 3H, 9-H, 2-H_{benzyl}, 6-H_{benzyl}), 7.10 (ddd, J = 8.1/6.9/1.2 Hz, 1H, 7-H), 7.12 – 7.18 (m, 2H, 3-H_{benzyl}, 5-H_{benzyl}), 7.17 – 7.21 (m, 1H, 4-H_{benzyl}), 7.47 (dt, J = 8.2/1.0 Hz, 1H, 6-H), 10.82 (s, 1H, 5-H). ^{13}C NMR (151 MHz, DMSO-*d*$_6$): δ (ppm) = 36.1 (1C, C-4), 36.8 (1C, Ph*C*H_2CH), 37.9 (1C, C-10), 42.0 (1C, C-3a), 46.0 (1C, C-10a), 55.0 (1C, PhCH_2*C*H), 55.6 (2C, 3-OCH_3, 5-OCH_3), 60.0 (1C, 4-OCH_3), 66.8 (1C, O*C*H_2CH), 106.1 (2C, C-2_{TMP}, C-6_{TMP}), 110.5 (1C, C-9b), 111.9 (1C, C-6), 118.7 (1C, C-9), 119.0 (1C, C-8), 121.9 (1C, C-7), 125.3 (1C, C-9a), 126.8 (1C, C-4_{benzyl}), 128.4 (1C, C-4a), 128.6 (2C, C-3_{benzyl}, C-5_{benzyl}), 129.3 (2C, C-2_{benzyl}, C-6_{benzyl}), 135.1 (1C, C-1_{TMP}), 135.7 (1C, C-1_{benzyl}), 136.6 (1C, C-5a), 136.7 (1C, C-4_{TMP}), 152.5 (2C, C-3_{TMP}, C-5_{TMP}), 154.1 (1C, C=$O_{oxazolidine}$) 169.8 (1C, C-4-C=O), 170.6 (1C, C-1), 173.5 (1C, C-3). Exact mass (APCI): m/z = 611.2026 (calcd. 611.2024 for $C_{34}H_{31}N_3O_9$ [MH]$^+$). IR (neat): ṽ [cm^{-1}] = 2978, 2886 (w, C-$H_{aliph.}$), 1771, 1755, 1685 (s, C=O), 1127 (s, C-O), 745 (s, C-$H_{arom.}$).

For recrystallization, the previously obtained mixture of (+)-**3a** and (+)-**3d** was dissolved in a mixture of ethyl acetate and *tert*-butyl methyl ether under reflux. The solution was allowed to cool down to room temperature. The formed precipitate was filtered off and washed with cold *tert*-butyl methyl ether (2 x 10 mL) to give (+)-**3d**. The filtrate was concentrated in vacuo to give (+)-**3a**.

(+)-**3d** (precipitate obtained after recrystallization): Colorless solid, mp 229 °C, yield 15 mg (2 %). $C_{34}H_{30}N_2O_9$ (610.6). TLC: Decomposition during TLC. Purity (HPLC): 92.5 %, (t_R = 22.9 min). Specific rotation: $[\alpha]_D^{20}$ = +30 (c = 1.3, $CHCl_3$). ^{1}H NMR (600 MHz, $CDCl_3$): δ (ppm) = 2.84 (dd, J = 13.4/9.6 Hz, 1H, PhC*H_2*CH), 3.36 (dd, J = 13.4/3.5 Hz, 1H (PhC*H_2*CH), 3.74 (s, 6H, 3-OCH_3, 5-OCH_3), 3.88 (s, 3H, 4-OCH_3), 3.97 (dd, J = 8.9/1.8 Hz, 1H, 3a-H), 4.21 – 4.34 (m, 3H, 10a-H, OC*H_2*CH), 4.55 – 4.63 (m, 1H, PhCH_2C*H*), 4.69 (d, J = 9.9 Hz, 1H, 10-H), 6.02 (d, J = 1.8 Hz, 1H, 4-H), 6.39 (s, 2H, 2-H_{TMP}, 6-H_{TMP}), 6.66 (d, *J* = 8.1 Hz, 1H, 9-H), 6.87 (ddd, J = 8.1/7.0/1.0 Hz, 1H, 8-H), 7.14 (ddd, J = 8.2/7.0/1.2 Hz, 1H, 7-H), 7.19 – 7.23 (m, 2H, 2-H_{benzyl}, 6-H_{benzyl}), 7.28 – 7.42 (m, 4H, 6-H, 3-H_{benzyl}, 4-H_{benzyl}, 5-H_{benzyl}), 8.85 (s, 1H, 5-H). ^{13}C NMR (151 MHz, $CDCl_3$): δ (ppm) = 37.9 (1C, Ph*C*H_2CH), 38.3 (1C, C-4), 40.0 (1C, C-10), 44.6 (1C, C-3a), 47.2 (1C, C-10a), 56.1 (1C, PhCH_2*C*H), 56.3 (2C, 3-OCH_3, 5-OCH_3), 61.2 (1C, 4-OCH_3), 67.1 (O*C*H_2CH), 107.0 (2C, C-2_{TMP}, C-6_{TMP}), 111.5 (1C, C-9b), 111.5 (1C, C-6), 120.1 (1C, C-8), 120.7 (1C, C-9), 123.2 (1C, C-7), 125.9 (1C, C-9a), 127.1 (1C, C-4a), 127.9 (1C, C-4_{benzyl}), 129.4 (2C, C-3_{benzyl}, C-5_{benzyl}), 129.5 (2C, C-2_{benzyl}, C-

6$_{benzyl}$) , 133.6 (1C, C-1$_{TMP}$), 134.6 (1C, C-1$_{benzyl}$), 136.2 (1C, C-5a), 138.0 (1C, C-4$_{TMP}$), 153.3 (2C, C-3$_{TMP}$, C-5$_{TMP}$), 154.7 (1C, CO$_{oxazolidine}$), 169.0 (1C, C-4-*C*=O), 169.9 (1C, C-1), 171.5 (1C, C-3). Exact mass (APCI): m/z = 611.2034 (calcd. 611.2024 for $C_{34}H_{31}N_3O_9$ [MH]$^+$). IR (neat): ṽ [cm^{-1}] = 2978, 2886 (w, C-H$_{aliph.}$), 1786, 1755, 1697 (s, C=O), 1107 (s, C-O), 764 (s, C-H$_{arom.}$). CCDC number: 1951235.

(+)-**3a** (compound isolated from the filtrate after recrystallization): Colorless solid, mp 187 °C, yield 170 mg (28 %). $C_{34}H_{30}N_2O_9$ (610.6). TLC: Decomposition during TLC. Purity (HPLC): 94.0 %, (t_R = 22.8 min). Specific rotation: $[\alpha]_D^{20}$ = +48 (c = 0.8, $CHCl_3$). ^{1}H NMR (600 MHz, $CDCl_3$): δ (ppm) = 2.76 (dd, J = 13.8/11.0 Hz, 1H, PhC*H$_2$*CH), 3.65 (dd, J = 13.7/3.1 Hz, 1H, PhC*H$_2$*CH), 3.74 (s, 6H, 3-OCH_3, 5-OCH_3), 3.75 (s, 3H, 4-OCH_3), 3.89 (dd, J = 9.2/7.9 Hz, 1H, 10a-H), 4.20 – 4.29 (m, 2H, OC*H$_2$*CH), 4.56 (dd, J = 10.2/9.1 Hz, 1H, 3a-H), 4.69 – 4.74 (m, 1H,OCH_2C*H*), 4.86 (d, J = 7.9 Hz, 1H, 10-H), 5.39 (d, J = 10.1 Hz, 1H, 4-H), 6.53 (s, 2H, 2-H$_{TMP}$, 6-H$_{TMP}$), 7.03 (ddd, J = 7.9/7.0/0.9 Hz, 1H, 8-H), 7.16 – 7.22 (m, 3H, 7-H, 2-H$_{benzyl}$, 6-H$_{benzyl}$), 7.25 – 7.39 (m, 5H, 5-H, 8-H, 3-H$_{benzyl}$, 4-H$_{benzyl}$, 5-H$_{benzyl}$), 8.25 (s, 1H, 5-H). ^{13}C NMR (151 MHz, $CDCl_3$): δ (ppm) = 37.1 (1C, Ph*C*H$_2$CH), 38.1 (1C, C-4), 38.6 (1C, C-10), 40.4 (1C, C-3a), 47.3 (1C, C-10a), 56.2 (2C, 3-OCH_3, 5-OCH_3), 56.7 (1C, PhCH$_2$*C*H), 61.0 (1C, 4-OCH_3), 67.3 (1C, O*C*H$_2$CH), 106.5 (2C, C-2$_{TMP}$, C-6$_{TMP}$), 111.3 (1C, C-6), 112.8 (1C, C-9b), 118.8 (1C, C-8), 120.3 (1C, C-9), 123.5 (1C, C-7), 125.5 (1C, C-9a), 127.5 (1C, C-4$_{benzyl}$), 128.4 (1C, C-4a), 129.3 (2C, C-3$_{benzyl}$, C-5$_{benzyl}$), 129.4 (2C, C-2$_{benzyl}$, C-6$_{benzyl}$) 133.3 (1C, C-1$_{TMP}$), 135.8 (1C, C-1$_{benzyl}$), 137.2 (1C, C-5a), 137.8 (1C, C-4$_{TMP}$), 153.3 (2C, C-3$_{TMP}$, C-5$_{TMP}$) , 154.0 (1C, C=O$_{oxazolidine}$), 169.5 (1C, C-1), 169.9 (1C, C-4-*C*=O), 171.1 (1C, C-3). Exact mass (ESI): m/z = 611.1989 (calcd. 611.2024 for $C_{34}H_{31}N_3O_9$ [MH]$^+$). IR (neat): ṽ [cm^{-1}] = 2978, 2947 (w, C-H$_{aliph.}$), 1782, 1701 (s, C=O), 1123 (s, C-O), 741 (s, C-H$_{arom.}$).

(3a*S*,4*R*,10*R*,10a*R*)-4-{[(*R*)-4-Benzyl-2-oxo-1,3-oxazolidin-3-yl]carbonyl}-10-(3,4,5-trimethoxyphenyl)-4,5,10,10a-tetrahydrofuro[3,4-*b*]carbazole-1,3(3a*H*)-dione ((-)-3a)

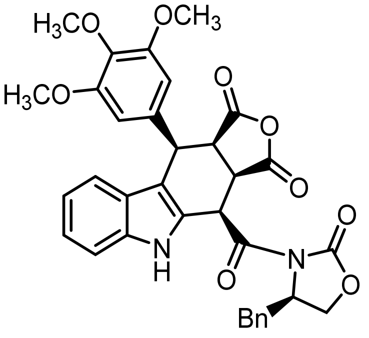

As described for the synthesis of (+)-**3a**, indole (*R*)-**4** (351 mg, 1.05 mmol), maleic anhydride (**6**, 314 mg, 3.21 mmol), 3,4,5-trimethoxybenzaldehyde (**5**, 311 mg, 1.59 mmol) and $CuSO_4 \cdot 5\ H_2O$ (28.4 mg, 0.11 mmol) were transformed. Work-up and purification were performed as described above. Colorless solid, mp 187 °C, yield 221 mg (36 %). $C_{34}H_{30}N_2O_9$ (610.6). TLC: Decomposition during TLC. Purity (HPLC): 94.6 %, (t_R = 22.5 min). Specific rotation: $[\alpha]_D^{20}$ = -48 (c = 1.5, $CHCl_3$).

(3a*R*,4*R*,10*R*,10a*R*)-4-{[(*R*)-4-Benzyl-2-oxo-1,3-oxazolidin-3-yl]carbonyl}-10-(3,4,5-trimethoxyphenyl)-4,5,10,10a-tetrahydropyrrolo[3,4-*b*]carbazole-1,3(2*H*,3a*H*)-dione ((-)-9a) and
(3a*S*,4*S*,10*S*,10a*S*)-4-{[(*R*)-4-Benzyl-2-oxo-1,3-oxazolidin-3-yl]carbonyl}-10-(3,4,5-trimethoxyphenyl)-4,5,10,10a-tetrahydropyrrolo[3,4-*b*]carbazole-1,3(2*H*,3a*H*)-dione ((+)-9b)

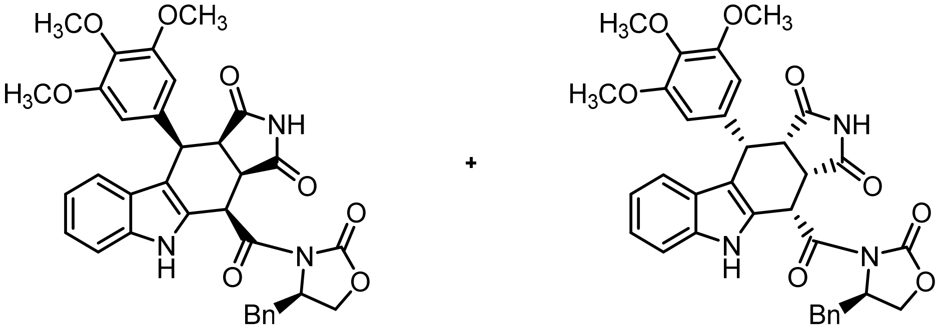

Under N_2, indole (*R*)-**4** (168 mg, 0.50 mmol), maleimide (**7**, 146 mg, 1.50 mmol) and 3,4,5-trimethoxybenzaldehyde (**5**, 147 mg, 0.75 mmol) were dissolved in dry toluene (10 mL) in a pressure resistant Schlenk tube. Crushed $CuSO_4 \cdot 5\ H_2O$ (12.6 mg, 0.05 mmol) was added to the solution and the mixture was heated to reflux for 22 h (oil bath temperature 130 °C). After cooling to room temperature, the mixture was filtered

and the filter was washed with CH_2Cl_2 (3 x 10 mL). The filtrate was concentrated in vacuo and the residue was purified by flash column chromatography (ethyl acetate/cyclohexane = 45:55, Ø 3 cm, h = 18 cm, v = 20 mL). The first obtained fraction was again purified by flash column chromatography (ethyl acetate/CH_2Cl_2 = 2:8, Ø 4 cm, h = 18 cm, v = 30 mL) to give (-)-**9a**. The second obtained fraction was also purified once more by flash column chromatography (ethyl acetate/CH_2Cl_2 = 3:7, Ø 4 cm, h = 19 cm, v = 30 mL) to give (+)-**9b**.

(-)-**9a** [R_f = 0.41 (ethyl acetate/CH_2Cl_2 = 2:8)]: Yellow solid, mp 216 °C, yield 107 mg (35 %). $C_{34}H_{31}N_3O_8$ (609.6). Purity (HPLC): 99.5 %, (t_R = 21.3 min). Specific rotation: $[\alpha]_D^{20}$ = -80 (c = 3.6, $CHCl_3$). ^1H NMR (600 MHz, DMSO-d_6): δ (ppm) = 2.85 (dd, J = 13.6/10.1 Hz, 1H, PhC*H₂*CH), 3.46 (dd, J = 13.6/2.9 Hz, 1H, PhC*H₂*CH), 3.55 (s, 3H, 4-OCH_3), 3.58 (t, J = 8.3 Hz, 1H, 10a-H), 3.67 (s, 6H, 3-OCH_3, 5-OCH_3), 4.31 (dd, J = 9.0/2.2 Hz, 1H, $CH_{2\,oxazolidine}$), 4.35 (dd, J = 11.6/8.6 Hz, 1H, 3a-H), 4.42 (t, J = 8.4 Hz, 1H, $CH_{2\,oxazolidine}$), 4.65 – 4.72 (m, 1H, $CH_{oxazolidine}$), 4.72 (d, J = 7.6 Hz, 1H, 10-H), 5.35 (d, J = 11.6 Hz, 1H, 4-H), 6.72 (s, 2H, 2-H_{TMP}, 6-H_{TMP}), 6.91 (ddd, J = 8.0/6.9/0.7 Hz, 1H, 8-H), 7.05 (ddd, J = 8.2/7.0/1.1 Hz, 1H, 7-H), 7.25 – 7.29 (m, 1H, 4-H_{benzyl}), 7.31 – 7.38 (m, 6H, 6-H, 9-H, 2-H_{benzyl}, 3-H_{benzyl}, 5-H_{benzyl}, 6-H_{benzyl}), 10.77 (s, 1H, 2-H), 10.81 (s, 1H, 5-H). ^{13}C NMR (151 MHz, DMSO-d_6): δ (ppm) = 36.1 (1C, C-4), 36.3 (1C, PhCH_2CH), 38.3 (1C, C-10), 40.2 (1C, C-3a), 47.3 (1C, C-10a), 55.7 (2C, 3-OCH_3, 5-OCH_3), 56.2 (1C, $CH_{oxazolidine}$), 59.8 (1C, 4-OCH_3), 66.7 (1C, $CH2_{oxazolidine}$), 106.8 (2C, C-2_{TMP}, C-6_{TMP}), 110.9 (1C, C-6), 111.7 (1C, C-9b), 118.1 (1C, C-9), 118.5 (1C, C-8), 121.6 (1C, C-7), 125.3 (1C, C-9a), 126.8 (1C, C-4_{benzyl}), 128.8 (2C, C-3_{benzyl}, C-5_{benzyl}), 129.3 (2C, C-2_{benzyl}, C-6_{benzyl}), 131.1 (1C, C-4a), 136.0 (1C, C-1_{TMP}), 136.3 (1C, C-4_{TMP}), 136.4 (1C, C-5a), 136.8 (C-1_{benzyl}), 152.1 (2C, C-3_{TMP}, C-5_{TMP}) 153.6 (1C, $CO_{oxazolidine}$), 171.5 (1C, C-4-*C*=O), 177.2 (1C, C-1), 178.6 (1C, C-3). Exact mass (ESI): m/z = 610.2210 (calcd. 610.2184 for $C_{34}H_{32}N_3O_8$ $[MH]^+$). IR (neat): ṽ [cm^{-1}] = 2978, 2889 (m, C-$H_{aliph.}$), 1775, 1701 (s, C=O), 1223, 1119 (s, C-O) 741 (s, C-$H_{arom.}$).

(+)-**9b** [R_f = 0.32 (ethyl acetate/CH_2Cl_2 = 2:8)]: Yellow solid, mp 225 °C, yield 70 mg (23 %). $C_{34}H_{31}N_3O_8$ (609.6). Purity (HPLC): 97.8 %, (t_R = 21.6 min). Specific rotation: $[\alpha]_D^{20}$ = +62 (c = 2.0, $CHCl_3$). ^1H NMR (600 MHz, DMSO-d_6): δ (ppm) = 2.99 (dd, J = 13.5/8.7 Hz, 1H, PhC*H₂*CH), 3.54 (dd, J = 8.9/8.0 Hz, 1H, 10a-H), 3.56 (s, 3H, 4-OCH_3), 3.66 (s, 6H, 3-OCH_3, 5-OCH_3), 4.23 (dd, J = 11.6/8.5 Hz, 1H, 3a-H), 4.30

(dd, J = 9.0/3.7 Hz, 1H, C$H_{2\,oxazolidine}$), 4.39 (t, J = 8.7 Hz, 1H, C$H_{2\,oxazolidine}$), 4.66 – 4.70 (m, 1H, C$H_{oxazolidine}$), 4.71 (d, J = 7.5 Hz, 1H, 10-H), 5.48 (d, J = 11.6 Hz, 1H, 4-H), 6.62 (s, 2H, 2-H_{TMP}, 6-H_{TMP}), 6.91 (ddd, J = 7.9/7.0/1.0 Hz, 1H, 8-H), 7.07 (ddd, J = 8.1/7.0/1.2 Hz, 1H, 7-H), 7.27 – 7.32 (m, 1H, 4-H_{benzyl}), 7.31 – 7.40 (m, 6H, 6-H, 9-H, 2-H_{benzyl}, 3-H_{benzyl}, 5-H_{benzyl}, 6-H_{benzyl}), 10.75 (s, 1H, 2-H), 11.02 (s, 1H, 5-H). Signal for the second PhCH_2CH proton is overlaid by the H_2O signal at 3.30 ppm. ^{13}C NMR (151 MHz, DMSO-*d*$_6$): δ (ppm) = 36.6 (1C, C-4), 37.8 (1C, PhCH2CH), 38.2 (1C, C-10), 47.5 (1C, C-10a), 55.7 (1C, *C*$H_{oxazoldine}$), 55.8 (2C, 3-OCH_3, 5-OCH_3), 59.8 (1C, 4-OCH_3), 66.6 (1C, *C*$H_{2\,oxazolidine}$), 106.6 (2C, C-2_{TMP}, C-6_{TMP}), 111.0 (1C, C-6), 111.9 (1C, C-9b), 118.1 (1C, C-9), 118.4 (1C, C-8), 121.6 (1C, C-7), 125.4 (1C, C-9a), 126.9 (1C, C-4_{benzyl}), 128.7 (2C, C-3_{benzyl}, C-5_{benzyl}), 129.4 (2C, C-2_{benzyl}, C-6_{benzyl}), 130.6 (1C, C-4a), 136.0 (1C, C-4_{TMP}), 136.2 (1C, C-1_{TMP}), 136.2 (C-1_{benzyl}), 136.6 (1C, C-5a), 152.1 (2C, C-3_{TMP}, C-5_{TMP}) 154.1 (1C, *C*$O_{oxazolidine}$), 171.6 (1C, C-4-*C*=O), 177.0 (1C, C-1), 178.8 (1C, C-3). Exact mass (ESI): m/z = 610.2151 (calcd. 610.2184 for $C_{34}H_{32}N_3O_8$ $[MH]^+$). IR (neat): ṽ [cm^{-1}] = 2978, 2889 (m, C-$H_{aliph.}$), 1770, 1713 (s, C=O), 1223, 1119 (s, C-O), 741 (s, C-$H_{arom.}$).

(3a*S*,4*S*,10*S*,10a*S*)-4-{[(*S*)-4-Benzyl-2-oxo-1,3-oxazolidin-3-yl]carbonyl}-10-(3,4,5-trimethoxyphenyl)-4,5,10,10a-tetrahydropyrrolo[3,4-*b*]carbazole-1,3(2*H*,3a*H*)-dione ((+)-9a) and (3a*R*,4*R*,10*R*,10a*R*)-4-{[(*S*)-4-Benzyl-2-oxo-1,3-oxazolidin-3-yl]carbony}-10-(3,4,5-trimethoxyphenyl)-4,5,10,10a-tetrahydropyrrolo[3,4-*b*]carbazole-1,3(2*H*,3a*H*)-dione ((-)-9b)

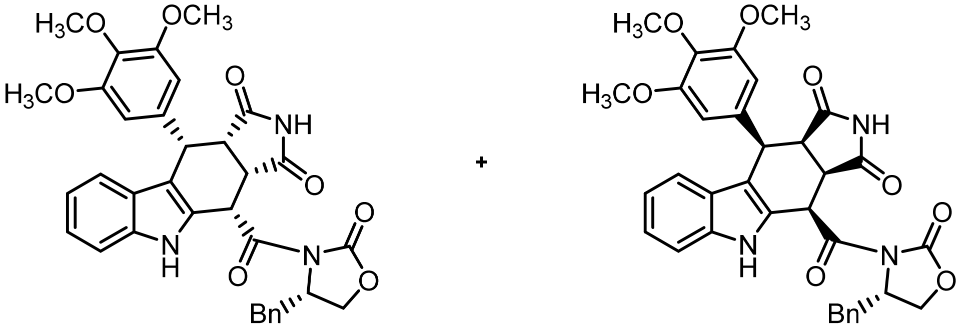

As described for the synthesis of (-)-**9a** and (+)-**9b**, indole (*S*)-**4** (335 mg, 1.0 mmol), maleimide (**7**, 290 mg, 2.99 mmol), 3,4,5-trimethoxybenzaldehyde (**5**, 295 mg, 1.50 mmol) and $CuSO_4 \cdot 5\ H_2O$ (25.6 mg, 0.10 mmol) were transformed. Work-up and purification were performed as described above.

(+)-**9a** [R_f = 0.50 (ethyl acetate/CH_2Cl_2 = 25:75)]: Yellow solid, mp 225 °C, yield 230 mg (38 %). $C_{34}H_{31}N_3O_8$ (609.6). Purity (HPLC): 94.6 %, (t_R = 21.3 min). Specific rotation: $[\alpha]_D^{20}$= +78 (c = 2.0, $CHCl_3$). Exact mass (APCI): m/z = 610.2210 (calcd. 610.2184 for $C_{34}H_{32}N_3O_8$ $[MH]^+$).

(-)-**9b** [R_f = 0.37 (ethyl acetate/CH_2Cl_2 = 25:75)]: Yellow solid, mp 230 °C, yield 160 mg (26 %). $C_{34}H_{31}N_3O_8$ (609.6). Purity (HPLC): 97.5 %, (t_R = 21.5 min). Specific rotation: $[\alpha]_D^{20}$= -59 (c = 1.7, $CHCl_3$). Exact mass (ESI): m/z = 610.2182 (calcd. 610.2184 for $C_{34}H_{32}N_3O_8$ $[MH]^+$).

(3a*S*,4*R*,10*S*,10a*S*)-4-{[(*S*)-4-Benzyl-2-oxo-1,3-oxazolidin-3-yl]carbonyl}-10-(3,4,5-trimethoxyphenyl)-4,5,10,10a-tetrahydropyrrolo[3,4-*b*]carbazole-1,3(2*H*,3a*H*)-dione ((+)-9c)

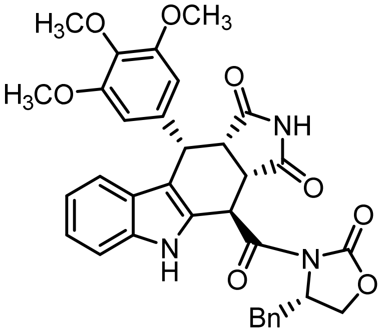

Under N_2, phenylalaninol amide (+)-**14c** (202 mg, 0.35 mmol) was dissolved in dry DMF (10 mL) at room temperature. CDI (111mg, 0.71 mmol) was added to the solution and the mixture was stirred for 16 h at 60 °C. After completion of the transformation, ethyl acetate (50 mL) was added and the solution was washed with HCl (0.5 M in water, 3 x 30 mL) and saturated NaCl solution (1 x 30 mL). The organic layer was dried (Na_2SO_4), filtered and concentrated in vacuo. The residue was purified by flash column chromatography (ethyl acetate/CH_2Cl_2 = 4:6 → 8:2, Ø 3 cm, h = 20 cm, v = 20 mL).

Colorless solid, mp 248 °C, yield 42 mg (20 %). $C_{34}H_{31}N_3O_8$ (609.6). TLC (ethyl acetate/CH_2Cl_2) = 4:6: R_f = 0.35. Purity (HPLC): 99.0 %, (t_R = 21.2 min). Enantiomeric excess (chiral HPLC): 97.1 % (t_R = 22.8 min). Specific rotation: $[\alpha]_D^{20}$ = +135 (c = 1.1, THF). ^1H NMR (600 MHz, DMSO-d_6): δ (ppm) = 3.09 (dd, J = 13.5/9.1 Hz, 1H, PhCH_2CH), 3.18 (dd, J = 13.5/3.7 Hz, 1H, PhCH_2CH), 3.59 (s, 3H, 4-OCH_3), 3.62 (t, J = 8.7 Hz, 1H, 10a-H), 3.64 (s, 6H, 3-OCH_3, 5-OCH_3), 4.12 (dd, J = 9.1/4.1 Hz, 1H, 3a-H), 4.28 (dd, J = 8.7/3.7 Hz, 1H, OCH_2CH), 4.47 (t, J = 8.6 Hz, 1H, OCH_2CH), 4.78 (d, J = 8.3 Hz, 1H, 10-H), 4.76 – 4.83 (m, 1H, $PhCH_2$C*H*), 5.98 (d,

J = 4.2 Hz, 1H, 4-H), 6.34 (s, 2H, 2-H_{TMP}, 6-H_{TMP}), 6.90 (ddd, J = 7.9/7.0/0.9 Hz, 1H, 8-H), 7.08 (ddd, J = 8.0/7.0/1.1 Hz, 1H, 7-H), 7.13 – 7.15 (m, 2H, 2-H_{benzyl}, 6-H_{benzyl}), 7.17 (d, J = 8.0 Hz, 1H, 9-H), 7.19 – 7.24 (m, 3H, 3-H_{benzyl}, 4-H_{benzyl}, 5-H_{benzyl}), 7.44 (dt, J = 8.1/0.9 Hz, 1H, 6-H), 10.75 (s, 1H, 5-H), 10.98 (s, 1H, 2-H). ^{13}C NMR (151 MHz, DMSO-*d6*): δ (ppm) = 36.4 (1C, C-4), 36.9 (1C, Ph*C*H_2CH), 38.3 (1C, C-10), 43.1 (1C, C-3a), 46.6 (1C, C-10a), 55.3 (1C, PhCH_2*C*H), 55.5 (2C, 3-OCH_3, 5-OCH_3), 59.9 (1C, 4-OCH_3), 66.7 (1C, O*C*H_2CH), 106.1 (2C, C-2_{TMP}, C-6_{TMP}), 111.7 (1C, C-6), 111.9 (1C, C-9b), 118.4 (1C, C-9), 118.8 (1C, C-8), 121.5 (1C, C-7), 125.4 (1C, C-9a), 126.8 (C-4_{benzyl}), 128.6 (2C, C-3_{benzyl}, C-5_{benzyl}), 129.2 (1C, C-4a), 129.3 (2C, C-2_{benzyl}, C-6_{benzyl}), 135.9 (1C, C-1_{benzyl}), 136.1 (1C, C-4_{TMP}), 136.2 (1C, C-1_{TMP}), 136.5 (1C, C-5a), 152.2 (2C, C-3_{TMP}, C-5_{TMP}), 153.9 (1C, $CO_{oxazolidne}$), 170.6 (1C, C-4-*C*=O), 177.9 (1C, C-1), 179.5 (1C, C-3). Exact mass (ESI): m/z = 610.2166 (calcd. 610.2184 for $C_{34}H_{32}N_3O_8$ [MH]$^+$). IR (neat): ṽ [cm^{-1}] = 1767, 1713, 1686 (s, C=O), 1123 (s, C-O), 764, 748 (m, C-$H_{arom.}$).

(3a*R*,4*S*,10*R*,10a*R*)-4-{[(*R*)-4-Benzyl-2-oxo-1,3-oxazolidin-3-yl]carbonyl}-10-(3,4,5-trimethoxyphenyl)-4,5,10,10a-tetrahydropyrrolo[3,4-*b*]carbazole-1,3(2*H*,3a*H*)-dione ((-)-9c, WMS-66-41)

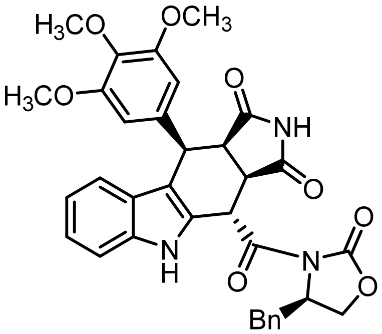

As described for the synthesis of (+)-**9c**, phenylalaninol amide (-)-**14c** (175 mg, 0.30 mmol) and CDI (97.7 mg, 0.60 mmol) were transformed. Work-up and purification was performed as described above. After the first fc purification, further purification by flash column chromatography (acetone/CH_2Cl_2 = 2:8, Ø 2 cm, h = 20 cm, v = 10 mL) was required. Colorless solid, mp 245 °C, yield 32 mg (18 %). $C_{34}H_{31}N_3O_8$ (609.6). TLC (ethyl acetate/CH_2Cl_2) = 4:6): R_f = 0.35. Purity (HPLC): 97.5 %, (t_R = 21.1 min). Enantiomeric excess (chiral HPLC): 99.8 % (t_R = 61.9 min). Specific rotation: $[\alpha]_D^{20}$ = - 41 (c = 1.0, THF). Exact mass (ESI): m/z = 610.2156 (calcd. 610.2184 for $C_{34}H_{32}N_3O_8$ [MH]$^+$).

(3a*R*,4*S*,10*R*,10a*R*)-4-{[(*S*)-4-Benzyl-2-oxo-1,3-oxazolidin-3-yl]carbonyl}-10-(3,4,5-trimethoxyphenyl)-4,5,10,10a-tetrahydropyrrolo[3,4-*b*]carbazole-1,3(2*H*,3a*H*)-dione ((-)-9d, WMS-66-36)

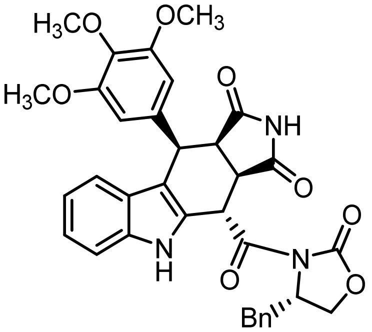

Under N_2, phenylalaninol amide (-)-**14d** (297 mg, 0.36 mmol) was dissolved in dry DMF (5 mL) at room temperature. CDI (114mg, 0.71 mmol) was added to the solution and the mixture was stirred for 16 h at 60 °C. After completion of the transformation, ethyl acetate (30 mL) was added and the solution was washed with HCl (0.5 M in water, 3 x 25 mL) and saturated NaCl solution (1 x 25 mL). The organic layer was dried (Na_2SO_4), filtered and concentrated in vacuo. The residue was purified by flash column chromatography (ethyl acetate/CH_2Cl_2 = 4:6, Ø 3 cm, h = 23 cm, v = 20 mL). Colorless solid, mp 190 °C, yield 92 mg (42 %). $C_{34}H_{31}N_3O_8$ (609.6). TLC (ethyl acetate/CH_2Cl_2 = 4:6): R_f = 0.55. Purity (HPLC): 95.9 %, (t_R = 20.9 min). Enantiomeric excess (chiral HPLC): 92.1 % (t_R = 27.6 min). Specific rotation: $[\alpha]_D^{20}$ = - 31 (c = 1.3, THF). ^{1}H NMR (600 MHz, DMSO-*d6*): δ (ppm) = 3.02 – 3.17 (m, 2H, PhC*H2*CH), 3.56 (s, 3H, 4-OCH_3), 3.61 – 3.66 (m, 1H, 10a-H), 3.64 (s, 6H, 3-OCH_3, 5-OCH_3), 4.28 – 4.34 (m, 2H, 3a-H, OC*H2*CH), 4.57 (t, J = 8.6 Hz, 1H, OC*H2*CH), 4.88 (d, J = 7.8 Hz, 1H, 10-H), 4.95 (ddt, J = 9.1/6.3/3.2 Hz, 1H, $PhCH_2C$*H*), 5.97 (d, J = 6.9 Hz, 1H, 4-H), 6.37 (s, 2H, 2-H_{TMP}, 6-H_{TMP}), 6.96 (ddd, J = 8.0/7.1/1.0 Hz, 1H, 8-H), 7.08 (ddd, J = 8.2/7.1/1.2 Hz, 1H, 7-H), 7.24 – 7.37 (m, 4H, 6-H, 3-H_{benzyl}, 4-H_{benzyl}, 5-H_{benzyl}), 7.39 – 7.43 (m, 2H, 2-H_{benzyl}, 6-H_{benzyl}), 7.47 (d, J = 7.9 Hz, 1H, 9-H), 11.03 (s, 1H, 5-H), 11.08 (s, 1H, 2-H). ^{13}C NMR (151 MHz, DMSO-*d6*): δ (ppm) = 36.0 (1C, Ph*C*H_2CH), 37.4 (1C, C-4), 37.9 (1C, C-10), 43.8 (1C, C-3a), 46.8 (1C, C-10a), 54.8 (1C, $PhCH_2$*C*H), 55.5 (2C, 3-OCH_3, 5-OCH_3), 59.9 (1C, 4-OCH_3), 66.0 (1C, O*C*H_2CH), 105.4 (2C, C-2_{TMP}, C-6_{TMP}), 111.2 (1C, C-6), 112.5 (1C, C-9b), 117.9 (1C, C-9), 119.0 (1C, C-8), 121.5 (1C, C-7), 125.2 (1C, C-9a), 127.0 (C-4_{benzyl}), 128.5 (2C, C-3_{benzyl}, C-5_{benzyl}), 129.9 (2C, C-2_{benzyl}, C-6_{benzyl}), 130.1 (1C, C-4a), 135.2 (1C, C-1_{benzyl}), 136.0 (1C, C-4_{TMP}), 136.0 (1C, C-5a), 136.2 (1C, C-1_{TMP}), 152.3 (2C, C-3_{TMP}, C-5_{TMP}), 153.5 (1C, $CO_{oxazolidne}$), 171.0 (1C, C-4-*C*=O), 178.0 (1C, C-1), 180.1 (1C, C-3). Exact mass (ESI): m/z = 610.2180 (calcd.

610.2184 for $C_{34}H_{32}N_3O_8$ [MH]$^+$). IR (neat): ṽ [cm^{-1}] = 2938 (m, C-H$_{aliph.}$), 1775, 1713 (s, C=O), 1215, 1119 (s, C-O), 745 (s, C-H$_{arom.}$).

(3a*S*,4*R*,10*S*,10a*S*)-4-{[(*R*)-4-Benzyl-2-oxo-1,3-oxazolidin-3-yl]carbonyl}-10-(3,4,5-trimethoxyphenyl)-4,5,10,10a-tetrahydropyrrolo[3,4-*b*]carbazole-1,3(2*H*,3a*H*)-dione ((+)-9d, WMS-66-40)

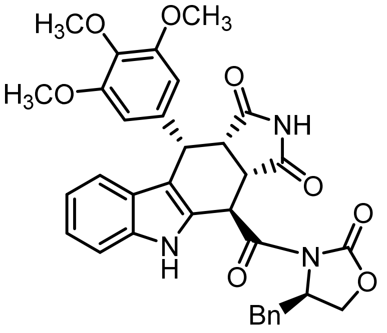

As described for the synthesis of (-)-**9d**, phenylalaninol amide (+)-**14d** (150 mg, 0.26 mmol) and CDI (88.2 mg, 0.54 mmol) were transformed. Work-up and purification was performed as described above. Colorless solid, mp 186 °C, yield 94 mg (59 %). $C_{34}H_{31}N_3O_8$ (609.6). Purity (HPLC): 98.6 %, (t_R = 20.9 min). Enantiomeric excess (chiral HPLC): 99.6 % (t_R = 15.5 min). R_f = 0.55. Specific rotation: $[\alpha]_D^{20}$ = +26 (c = 1.4, THF). Exact mass (ESI): m/z = 610.2208 (calcd. 610.2184 for $C_{34}H_{32}N_3O_8$ [MH]$^+$).

(3a*R*,4*R*,10*R*,10a*R*)-4-{[(*R*)-4-Benzyl-2-oxo-1,3-oxazolidin-3-yl]carbonyl}-2-methyl-10-(3,4,5-trimethoxyphenyl)-4,5,10,10a-tetrahydropyrrolo[3,4-*b*]carbazole-1,3(2*H*,3a*H*)-dione ((-)-10a) and (3a*S*,4*S*,10*S*,10a*S*)-4-{[(*R*)-4-Benzyl-2-oxo-1,3-oxazolidin-3-yl]carbonyl}-2-methyl-10-(3,4,5-trimethoxyphenyl)-4,5,10,10a-tetrahydropyrrolo[3,4-*b*]carbazole-1,3(2*H*,3a*H*)-dione ((+)-10b)

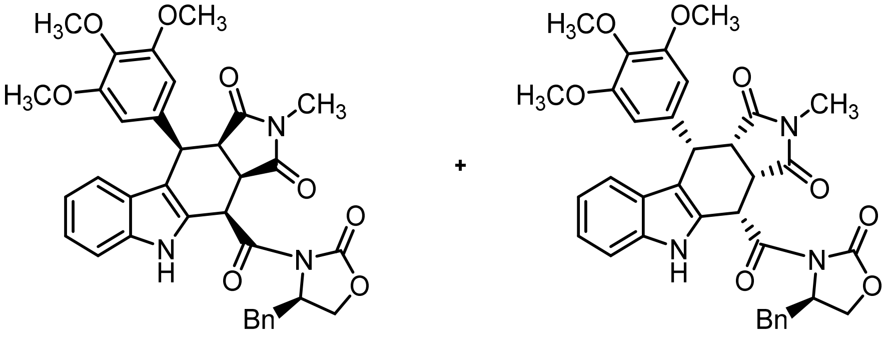

Under N_2, indole (*R*)-**4** (167 mg, 0.50 mmol), *N*-methylmaleimide (**8**, 166 mg, 1.49 mmol) and 3,4,5-trimethoxybenzaldehyde (**5**, 146 mg, 0.75 mmol) were dissolved in

dry toluene (15 mL) in a pressure resistant Schlenk tube. Crushed $CuSO_4 \cdot 5\ H_2O$ (12.9 mg, 0.05 mmol) was added to the solution and the mixture was heated to reflux for 16 h (oil bath temperature 130 °C). After cooling to room temperature, the mixture was filtered and the filter was washed with CH_2Cl_2 (3 x 10 mL). The filtrate was concentrated in vacuo and the residue was purified by flash column chromatography (ethyl acetate/cyclohexane = 3:7 → 1:1, Ø 3 cm, h = 20 cm, v = 20 mL). After repeated purification by flash column chromatography (ethyl acetate/CH_2Cl_2 = 10:90 → 15:85, Ø 4 cm, h = 20 cm, v = 30 mL), the isomers (-)-**10a** and (+)-**10b** were isolated.

(-)-**10a** [R_f = 0.22 (ethyl acetate/CH_2Cl_2 = 1:9)]: Yellow solid, mp 238 °C, yield 82 mg (26%). $C_{35}H_{33}N_3O_8$ (623.7). Purity (HPLC): 94.1 %, (t_R = 22.5 min). Specific rotation: $[\alpha]_D^{20}$ = -87 (c = 1.8, $CHCl_3$). ^1H NMR (600 MHz, DMSO-d_6): δ (ppm) = 2.22 (s, 3H, NHCH_3), 2.86 (dd, J = 13.6/10.3 Hz, 1H, PhCH_2CH), 3.51 (dd, J = 13.6/2.9 Hz, 1H, PhCH_2CH), 3.52 (s, 3H, 4-OCH_3), 3.65 (t, J = 7.8 Hz, 1H, 10a-H), 3.67 (s, 6H, 3-OCH_3, 5-OCH_3), 4.32 (dd, J = 9.0/2.2 Hz, 1H, C$H_{2\,oxazolidine}$), 4.36 (dd, J = 11.5/7.8 Hz, 1H, 3a-H), 4.43 (t, J = 8.5 Hz, 1H, C$H_{2\,oxazolidine}$), 4.69 (tt, J = 7.7/2.6 Hz, 1H, C$H_{oxazolidine}$), 4.72 (d, J = 7.6 Hz, 1H, 10-H), 5.38 (d, J = 11.5 Hz, 1H, 4-H), 6.63 (s, 2H, 2-H_{TMP}, 6-H_{TMP}), 6.91 (ddd, J = 8.0/7.0/0.9 Hz, 1H, 8-H), 7.06 (ddd, J = 8.1/7.0/1.2 Hz, 1H, 7-H), 7.25 – 7.31 (m, 1H, 4-H_{benzyl}), 7.31 – 7.39 (m, 6H, 6-H, 9-H, 2-H_{benzyl}, 3-H_{benzyl}, 5-H_{benzyl}, 6-H_{benzyl}), 10.81 (s, 1H, 5-H). ^{13}C NMR (151 MHz, DMSO-d_6): δ (ppm) = 23.3 (1C, NHCH$_3$), 36.4 (1C, C-4), 36.5 (1C, PhCH$_2$CH), 38.3 (1C, C-10), 39.1 (1C, C-3a), 46.7 (1C, C-10a), 55.8 (2C, 3-OCH_3, 5-OCH_3), 56.2 (1C, CH$_{oxazolidine}$), 59.8 (1C, 4-OCH_3), 66.8 (1C, CH$_{2\,oxazolidine}$), 106.6 (2C, C-2_{TMP}, C-6_{TMP}), 110.9 (1C, C-6), 111.1 (1C, C-9b), 118.1 (1C, C-9), 118.5 (1C, C-8), 121.6 (1C, C-7), 125.3 (1C, C-9a), 126.8 (1C, C-4_{benzyl}), 128.8 (2C, C-3_{benzyl}, C-5_{benzyl}), 129.3 (2C, C-2_{benzyl}, C-6_{benzyl}), 131.1 (1C, C-4a), 136.0 (1C, C-1_{TMP}), 136.1 (1C, C-4_{TMP}), 136.4 (1C, C-5a), 136.7 (C-1_{benzyl}), 152.1 (2C, C-3_{TMP}, C-5_{TMP}) 153.6 (1C, CO$_{oxazolidine}$), 171.4 (1C, C-4-C=O), 175.9 (1C, C-1), 177.1 (1C, C-3). Exact mass (APCI): m/z = 624.2302 (calcd. 624.2340 for $C_{35}H_{34}N_3O_8$ $[MH]^+$). IR (neat): ṽ [cm^{-1}] = 2978, 2889 (m, C-$H_{aliph.}$), 1771, 1701 (s, C=O), 1119 (s, C-O).

(+)-**10b** [R_f = 0.17 (ethyl acetate/CH_2Cl_2 = 1:9)]: Yellow solid, mp 216 °C, yield 75 mg (24%). $C_{35}H_{33}N_3O_8$ (623.7). Purity (HPLC): 96.2 %, (t_R = 22.7 min). Specific rotation: $[\alpha]_D^{20}$ = +82 (c = 2.0, $CHCl_3$). ^1H NMR (400 MHz, DMSO-d_6): δ (ppm) = 2.19 (s, 3H, NHCH_3), 3.00 (dd, J = 13.5/8.8 Hz, 1H, PhCH_2CH), 3.37 (dd, J = 13.5/3.2 Hz, 1H, PhCH_2CH), 3.53 (s, 3H, 4-OCH_3), 3.63 (t, J = 7.7 Hz, 1H, 10a-H), 3.66 (s, 6H,

3-OCH_3, 5-OCH_3), 4.24 (dd, J = 11.5/8.0 Hz, 1H, 3a-H), 4.31 (dd, J = 9.0/3.8 Hz, 1H, C*H2*$_{oxazolidine}$), 4.42 (t, J = 8.7 Hz, 1H, C*H2*$_{oxazolidine}$), 4.72 (d, J = 7.3 Hz, 1H, 10-H), 4.70 – 4.77 (m, 1H, C*H*$_{oxazolidine}$), 5.51 (d, J = 11.5 Hz, 1H, 4-H), 6.54 (s, 2H, 2-H_{TMP}, 6-H_{TMP}), 6.92 (ddd, J = 8.0/7.0/1.0 Hz, 1H, 8-H), 7.08 (ddd, J = 8.2/7.0/1.2 Hz, 1H, 7-H), 7.25 – 7.43 (m, 7H, 6-H, 9-H, 2-H_{benzyl}, 3-H_{benzyl}, 4-H_{benzyl}, 5-H_{benzyl}, 6-H_{benzyl}), 11.04 (s, 1H, 5-H). ^{13}C NMR (151 MHz, DMSO-*d6*): δ (ppm) = 23.3 (1C, NH*CH3)*, 36.8 (1C, C-4), 37.8 (1C, PhCH2CH), 38.2 (1C, C-10), 38.4 (1C, C-3a), 46.8 (1C, C-10a), 55.6 (1C, *C*H$_{oxazoldine}$), 55.9 (2C, 3-OCH_3, 5-OCH_3), 59.8 (1C, 4-OCH_3), 66.7 (1C, *C*H2$_{oxazolidine}$), 106.5 (2C, C-2$_{TMP}$, C-6$_{TMP}$), 111.0 (1C, C-6), 111.3 (1C, C-9b), 118.1 (1C, C-9), 118.4 (1C, C-8), 121.6 (1C, C-7), 125.3 (1C, C-9a), 126.8 (1C, C-4$_{benzyl}$), 128.8 (2C, C-3$_{benzyl}$, C-5$_{benzyl}$), 129.3 (2C, C-2$_{benzyl}$, C-6$_{benzyl}$), 131.0 (1C, C-4a), 135.9 (1C, C-4$_{TMP}$), 136.1 (1C, C-1$_{TMP}$), 136.1 (1C, C-5a), 136.6 (C-1$_{benzyl}$), 152.1 (2C, C-3$_{TMP}$, C-5$_{TMP}$) 154.0 (1C, *C*O$_{oxazolidine}$), 171.5 (1C, C-4-*C*=O), 175.7 (1C, C-1), 177.3 (1C, C-3). Exact mass (APCI): m/z = 624.2301 (calcd. 624.2340 for $C_{35}H_{34}N_3O_8$ $[MH]^+$). IR (neat): ṽ [cm^{-1}] = 2978, 2889 (m, C-$H_{aliph.}$), 1767, 1698 (s, C=O), 1119 (s, C-O), 741 (s, C-$H_{arom.}$). CCDC number: 1951236.

(3a*S*,4*S*,10*S*,10a*S*)-4-{[(*S*)-4-Benzyl-2-oxo-1,3-oxazolidin-3-yl]carbonyl}-2-methyl-10-(3,4,5-trimethoxyphenyl)-4,5,10,10a-tetrahydropyrrolo[3,4-*b*]carbazole-1,3(2*H*,3a*H*)-dione ((+)-10a) and (3a*R*,4*R*,10*R*,10a*R*)-4-{[(*S*)-4-Benzyl-2-oxo-1,3-oxazolidin-3-yl]carbonyl}-2-methyl-10-(3,4,5-trimethoxyphenyl)-4,5,10,10a-tetrahydropyrrolo[3,4-*b*]carbazole-1,3(2*H*,3a*H*)-dione ((-)-10b)

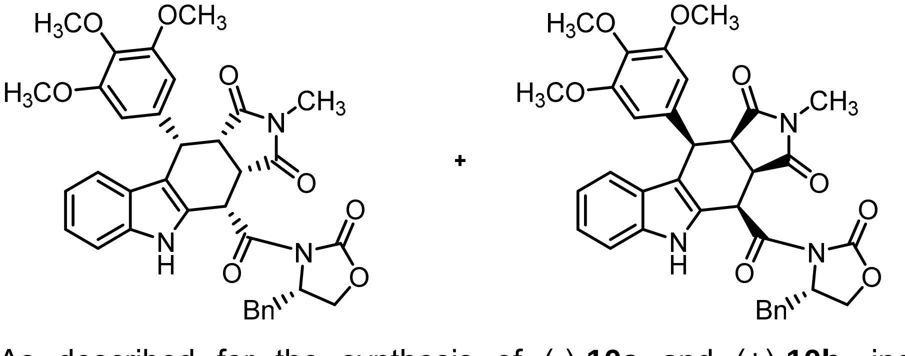

As described for the synthesis of (-)-**10a** and (+)-**10b**, indole (*S*)-**4** (333 mg, 1.00 mmol), *N*-methylmaleimide (**8**, 334 mg, 3.00 mmol), 3,4,5-trimethoxybenzaldehyde (**5**, 296 mg, 1.51 mmol) and $CuSO_4 \cdot 5\ H_2O$ (25.2 mg, 0.10 mmol) were transformed. Work-up and purification were performed as described above.

(+)-**10a** [R_f =0.57 (ethyl acetate/CH_2Cl_2 = 15:85)]: Yellow solid, mp 244 °C, yield 165 mg (26 %). $C_{35}H_{33}N_3O_8$ (623.7). Purity (HPLC): 99.1 %, (t_R = 22.4 min). Specific rotation: $[\alpha]_D^{20}$ = +89 (c = 1.8, $CHCl_3$). Exact mass (ESI): m/z = 624.2347 (calcd. 624.2340 for $C_{35}H_{34}N_3O_8$ $[MH]^+$).

(-)-**10b** [R_f = 0.45 (ethyl acetate/CH_2Cl_2)]: Yellow solid, mp 209 °C, yield 231 mg (37 %). $C_{35}H_{33}N_3O_8$ (623.7). Purity (HPLC): 95.4 %, (t_R = 22.7 min). Specific rotation: $[\alpha]_D^{20}$ = -78 (c = 2.0, $CHCl_3$). Exact mass (ESI): m/z = 624.2407 (calcd. 624.2340 for $C_{35}H_{34}N_3O_8$ $[MH]^+$).

(3a*RS*,4*SR*,10*RS*,10a*RS*)-1,3-Dioxo-10-(3,4,5-trimethoxyphenyl)-1,2,3,3a,4,5,10,10a-octahydropyrrolo[3,4-*b*]carbazole-4-carboxylic acid ((±)-12)

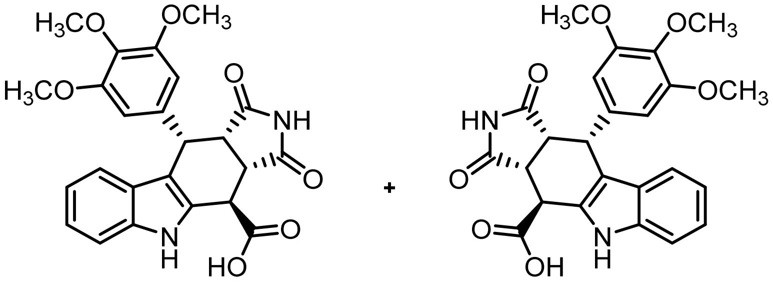

A solution of NaOH (2.60 g, 6.50 mmol) in water (50 mL) was added to a solution of ester (±)-**11c** (5.06g, 10.56 mmol) in THF (50 mL). The mixture was stirred for 30 min at room temperature. Ethyl acetate (100 mL) was added and the phases were separated. The aqueous layer was washed. with ethyl acetate (3 x 50 mL) to remove non-acidic impurities. HCl solution (2 M in water, 30 mL) was added to the aqueous layer and the resulting suspension was extracted with ethyl acetate (3 x 50 mL). The combined organic layers of the last extraction step were dried (Na_2SO_4), filtered and concentrated in vacuo to obtain the product without further purification. Yellow solid, mp 162 °C, yield 4.58 g (96 %). $C_{24}H_{22}N_2O_7$ (450.4). TLC (MeOH/CH_2Cl_2 = 15:85 + 0.1% acetic acid): R_f = 0.33. Purity (HPLC): 94.7 %, (t_R = 16.5 min). ^{1}H NMR (600 MHz, DMSO-*d6*): δ (ppm) = 3.54 (t, J = 8.4 Hz, 1H, 10a-H), 3.56 (s, 3H, 4-OCH_3), 3.62 (s, 6H, 3-OCH_3, 5-OCH_3), 4.09 (dd, J = 9.0/3.6 Hz, 1H, 3a-H), 4.41 (d, J = 3.6 Hz, 1H, 4-H), 4.72 (d, J = 8.2 Hz, 1H, 10-H), 6.22 (s, 2H, 2-H_{TMP}, 6-H_{TMP}), 6.87 (ddd, J = 7.8/6.8/0.9 Hz, 1H, 8-H), 7.02 (ddd, J = 8.2/7.0/1.2 Hz, 1H, 7-H), 7.21 (d, J = 7.9 Hz, 1H, 9-H), 7.39 (d, J = 8.1 Hz, 1H, 6-H), 10.89 (s, 1H, 2-H), 10.92 (s, 1H, 5-H), 13.40 (s, 1H, COO*H*). ^{13}C NMR (151 MHz, DMSO-*d6*): δ (ppm) = 37.0 (1C, C-4), 38.1 (1C, C-10), 42.3 (1C, C-3a), 45.5 (1C, C-10a), 55.6 (2C, 3-OCH_3, 5-OCH_3), 59.9 (1C, 4-

OCH_3), 106.0 (2C, C-2$_{TMP}$, C-6$_{TMP}$), 111.1 (1C, C-9b), 111.6 (1C, C-6), 118.2 (1C, C-9), 118.6 (1C, C-8), 121.4 (1C, C-7), 125.4 (1C, C-9a), 128.9 (1C, C-4a), 136.1 (1C, C-4$_{TMP}$), 136.5 (1C, C-1$_{TMP}$), 136.6 (1C, C-5a), 152.1 (2C, C-3$_{TMP}$, C-5$_{TMP}$), 172.4 (1C, *C*OOH), 177.8 (1C, C-1), 179.9 (1C, C-3). Exact mass (ESI): m/z = 451.1477 (calcd. 451.1500 for $C_{24}H_{23}N_2O_7$ $[MH]^+$). IR (neat): ṽ [cm^{-1}] = 2939, 2835 (m, C-H$_{aliph.}$), 1709 (s, C=O), 1119 (m, C-O), 745 (m, C-H$_{arom.}$).

(3a*R*,4*S*,10*R*,10a*R*)-*N*-[(*S*)-1-Hydroxy-3-phenylpropan-2-yl]-1,3-dioxo-10-(3,4,5-trimethoxyphenyl)-1,2,3,3a,4,5,10,10a-octahydropyrrolo[3,4-*b*]carbazole-4-carboxamide ((-)-14d) and
(3a*S*,4*R*,10*S*,10a*S*)-*N*-[(*S*)-1-Hydroxy-3-phenylpropan-2-yl]-1,3-dioxo-10-(3,4,5-trimethoxyphenyl)-1,2,3,3a,4,5,10,10a-octahydropyrrolo[3,4-*b*]carbazole-4-carboxamide ((+)-14c) and

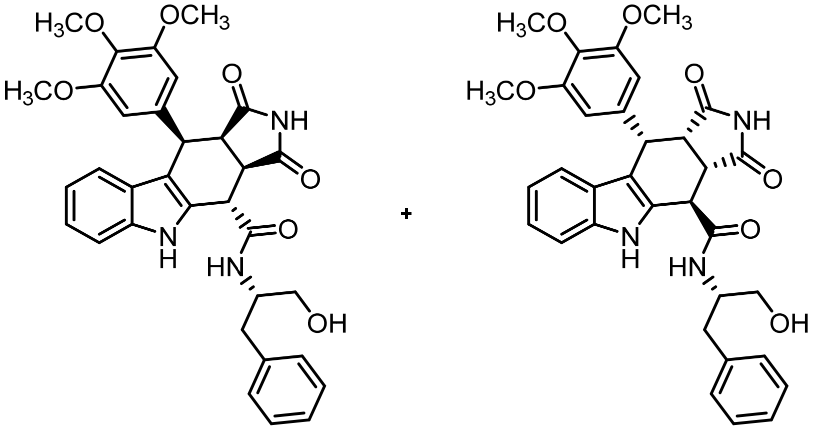

Under N_2, oxalyl chloride (469 mg, 3.61 mmol) and catalytic amounts of DMF were added to a solution of carboxylic acid (±)-**12c** (1.35 g, 3.01 mmol) in dry CH_2Cl_2 (20 mL) at room temperature. The mixture was stirred for 3 h at rt. The resulting suspension was concentrated in vacuo and the residue was dissolved in dry DMF (5 mL). This solution of the acid chloride was added dropwise to a solution of (*S*)-2-amino-3-phenylpropan-1-ol (599 mg, 3.96 mmol) and DIPEA (1.92 g, 14.9 mmol) in dry CH_2Cl_2 (20 mL). The mixture was stirred for 1 h at room temperature. After completion of the transformation, the resulting mixture was washed with HCl solution (0.5 M in water, 3 x 40 mL) and saturated NaCl solution (3 x 40 mL). The organic layer was dried (Na_2SO_4), filtered and concentrated in vacuo. The residue was purified by flash column chromatography (ethyl acetate/CH_2Cl_2 = 7:3, Ø 6 cm, h = 15 cm, v = 65 mL). At first (-)-**14d**, then (+)-**14c** was eluted.

(-)-**14d** [R_f = 0.38 (ethyl acetate/CH_2Cl_2 = 7:3)]: Yellow solid, mp 121 °C, yield 452 mg (26 %). $C_{33}H_{33}N_3O_7$ (583.6). Purity (HPLC): 95.1 %, (t_R = 18.2 min). Specific rotation: $[\alpha]_D^{20}$ = -163 (c = 1.8, THF). ^{1}H NMR (600 MHz, DMSO-d_6): δ (ppm) = 2.80 – 2.88 (m, 2H, PhC*H₂*CH), 3.46 (dd, J = 9.6/7.8 Hz, 1H, 10a-H), 3.51 (ddd, J = 10.7/7.1/5.1 Hz, 1H, CH_2OH), 3.56 (s, 3H, 4-OCH_3), 3.64 – 3.66 (m, 1H, CH_2OH), 3.67 (s, 6H, 3-OCH_3, 5-OCH_3), 3.95 (dd, J = 9.6/5.0 Hz, 1H, 3a-H), 4.15 – 4.22 (m, 1H, PhCH_2C*H*), 4.44 (d, J = 5.0 Hz, 1H, 4-H), 4.74 (d, J = 7.8 Hz, 1H, 10-H), 5.75 (t, J = 4.8 Hz, 1H, CH_2O*H*), 6.31 (s, 2H, 2-H_{TMP}, 6-H_{TMP}), 6.92 (ddd, J = 8.0/6.9/1.0 Hz, 1H, 8-H), 7.04 (ddd, J = 8.1/7.0/1.1 Hz, 1H, 7-H), 7.18 – 7.24 (m, 1H, 4-H_{phenyl}), 7.25 – 7.33 (m, 5H, 6-H, 2-H_{phenyl}, 3-H_{phenyl}, 5-H_{phenyl}, 6-H_{phenyl}), 7.40 (d, J = 7.9 Hz, 1H, 9-H), 8.69 (d, J = 8.6 Hz, 1H, NH_{amide}), 10.77 (s, 1H, 5-H), 10.89 (s, 1H, 2-H). ^{13}C NMR (151 MHz, DMSO-d_6): δ (ppm) = 37.1 (1C, Ph*C*H_2CH), 38.1 (1C, C-10), 38.8 (1C, C-4), 41.8 (1C, C-3a), 46.3 (1C, C10-a), 52.8 (1C, PhCH_2CH), 55.8 (2C, 3-OCH_3, 5-OCH_3), 59.9 (1C, 4-OCH_3), 61.8 (1C, *C*H_2OH), 105.8 (2C, C-2$_{TMP}$, C-6$_{TMP}$), 111.0 (1C, C-6), 111.8 (1C, C-9b), 117.9 (1C, C-9), 118.8 (1C, C-8), 121.3 (1C, C-7), 125.6 (1C, C-9a), 126.2 (1C, C-4$_{phenyl}$), 128.2 (2C, C-3$_{phenyl}$, C-5$_{phenyl}$), 129.2 (2C, C-2$_{phenyl}$, C-6$_{phenyl}$), 131.3 (1C, C-4a), 135.9 (1C, C-5a), 136.2 (1C, C-4$_{TMP}$), 137.0 (1C, C-1$_{TMP}$), 138.5 (1C, C-1$_{phenyl}$), 152.3 (2C, C-3$_{TMP}$, C-5$_{TMP}$), 169.7 (1C, CO_{amide}), 178.1 (1C, C-1), 180.5 (1C, C-3). Exact mass (ESI): m/z = 584.2387 (calcd. 584.2391 for $C_{33}H_{34}N_3O_7$ $[MH]^+$). IR (neat): ṽ [cm^{-1}] = 3278 (m, OH), 2978, 2940 (w, C-$H_{aliph.}$), 1713 (s, C=O), 1176, 1123 (s, C-O), 745 (s, C-$H_{arom.}$).

(+)-**14c** [R_f = 0.19 (ethyl acetate/CH_2Cl_2 = 7:3)]: Yellow solid, mp 103 °C, yield 490 mg (28 %). $C_{33}H_{33}N_3O_7$ (583.6). Purity (HPLC): 95.2 %, (t_R = 18.2 min). Specific rotation: $[\alpha]_D^{20}$ = +93 (c = 2.1, THF). ^{1}H NMR (400 MHz, DMSO-d_6): δ (ppm) = 2.88 (dd, J = 13.6/8.4 Hz, 1H, PhCH_2CH), 2.94 (dd, J = 13.7/6.2 Hz, 1H, PhCH_2CH), 3.34 – 3.63 (m, 2H, 10a-H, CH_2OH), 3.53 – 3.61 (m, 1H, CH_2OH), 3.57 (s, 3H, 4-OCH_3), 3.65 (s, 6H, 3-OCH_3, 5-OCH_3), 3.84 (dd, J = 9.1/3.8 Hz, 1H, 3a-H), 3.89 – 3.99 (m, 1H, PhCH_2C*H*), 4.40 (d, J = 3.8 Hz, 1H, 4-H), 4.67 (d, J = 8.2 Hz, 1H, 10-H), 5.05 (t, J = 5.2 Hz, 1H, CH_2O*H*), 6.25 (s, 2H, 2-H_{TMP}, 6-H_{TMP}), 6.87 (ddd, J = 8.0/7.0/1.0 Hz, 1H, 8-H), 7.03 (ddd, J = 8.2/7.0/1.2 Hz, 1H, 7-H), 7.18 (d, J = 8.0 Hz, 1H, 9-H), 7.20 – 7.26 (m, 5H, 2-H_{phenyl}, 3-H_{phenyl}, 4-H_{phenyl}, 5-H_{phenyl}, 6-H_{phenyl}), 7.28 (dd, J = 8.2/1.0 Hz, 1H, 6-H), 8.38 (d, J = 7.9 Hz, 1H, NH_{amide}), 9.89 (s, 1H, 5-H), 10.87 (s, 1H, 2-H). ^{13}C NMR (101 MHz, DMSO-d_6): δ (ppm) = 36.2 (1C, Ph*C*H_2CH), 38.2 (1C, C-10), 38.2 (1C, C-4), 42.8 (1C, C-3a), 46.1 (1C, C10-a), 53.8 (1C, PhCH_2*C*H), 55.7

(2C, 3-OCH3, 5-OCH3), 59.9 (1C, 4-OCH3), 62.0 (1C, *C*H2OH), 106.1 (2C, C-2TMP, C-6TMP), 111.2 (1C, C-9b), 111.2 (1C, C-6), 118.2 (1C, C-9), 118.7 (1C, C-8), 121.3 (1C, C-7), 125.6 (1C, C-9a), 126.1 (1C, C-4phenyl), 128.2 (2C, C-3phenyl, C-5phenyl), 129.1 (2C, C-2phenyl, C-6phenyl), 130.8 (1C, C-4a), 135.9 (1C, C-5a), 136.2 (1C, C-4TMP), 136.6 (1C, C-1TMP), 139.2 (1C, C-1phenyl), 152.1 (2C, C-3TMP, C-5TMP), 169.9 (1C, COamide), 178.0 (1C, C-1), 180.0 (1C, C-3). Exact mass (ESI): m/z = 584.2403 (calcd. 584.2391 for $C_{33}H_{34}N_3O_7$ $[MH]^+$). IR (neat): ṽ [cm^{-1}] = 3294 (m, OH), 2978, 2886 (m, C-Haliph.), 1709 (s, C=O), 1153, 1123 (s, C-O), 745 (s, C-Harom.).

(3a*S*,4*R*,10*S*,10a*S*)-*N*-[(*R*)-1-Hydroxy-3-phenylpropan-2-yl]-1,3-dioxo-10-(3,4,5-trimethoxyphenyl)-1,2,3,3a,4,5,10,10a-octahydropyrrolo[3,4-*b*]carbazole-4-carboxamide ((+)-14d) and

(3a*R*,4*S*,10*R*,10a*R*)-*N*-[(*R*)-1-Hydroxy-3-phenylpropan-2-yl]-1,3-dioxo-10-(3,4,5-trimethoxyphenyl)-1,2,3,3a,4,5,10,10a-octahydropyrrolo[3,4-*b*]carbazole-4-carboxamide ((-)-14c)

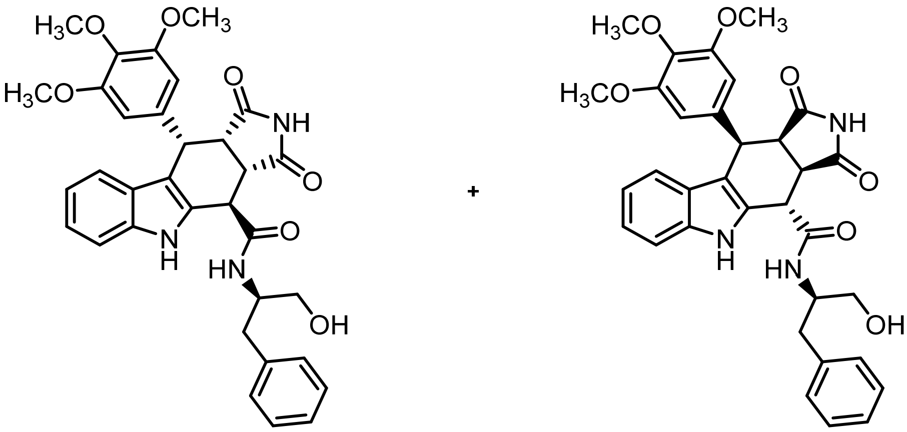

As described for (-)-**14d** and (+)-**14c**, carboxylic acid (±)-**12c** (1.25 g, 2.77 mmol), oxalyl chloride (444 mg, 3.50 mmol), catalytic amount of DMF, (*R*)-2-amino-3-phenylpropan-1-ol (547 mg, 3.60 mmol) and DIPEA (1.85 g, 14.3 mmol) were transformed. Work-up and purification was performed as described above.

(+)-**14d** [R_f = 0.38 (ethyl acetate/CH_2Cl_2 = 7:3)]: Yellow solid, mp 121 °C, yield 331 mg (20 %). $C_{33}H_{33}N_3O_7$ (583.6). Purity (HPLC): 95.1 %, (t_R = 18.1 min). Specific rotation: $[\alpha]_D^{20}$ = +153 (c = 2.4, THF). Exact mass (ESI): m/z = 584.2387 (calcd. 584.2391 for $C_{33}H_{34}N_3O_7$ $[MH]^+$). CCDC number: 1951237.

(-)-**14c** [R_f = 0.19 (ethyl acetate/CH_2Cl_2 = 7:3)]: Yellow solid, mp 103 °C, yield 399 mg (25 %). $C_{33}H_{33}N_3O_7$ (583.6). Purity (HPLC): 98.0 %, (t_R = 18.1 min). Specific rotation:

$[\alpha]_D^{20}$ = -94 (c = 2.5, THF). Exact mass (ESI): m/z = 584.2374 (calcd. 584.2391 for $C_{33}H_{34}N_3O_7$ $[MH]^+$).

6. X-Ray crystallography

X-Ray diffraction: Data sets for compounds (+)-**3d**, (+)-**10b** and (+)-**14d** were collected with a D8 Venture Dual Source 100 CMOS diffractometer. Programs used: data collection: APEX3 V2016.1-0 (Bruker AXS Inc., **2016**); cell refinement: SAINT V8.37A (Bruker AXS Inc., **2015**); data reduction: SAINT V8.37A (Bruker AXS Inc., **2015**); absorption correction, SADABS V2014/7 (Bruker AXS Inc., **2014**);[3] structure solution *SHELXT*-2015;[4] structure refinement *SHELXL*-2015[5] and graphics, *XP*.[6] *R*-values are given for observed reflections, and wR^2 values are given for all reflections.

Exceptions and special features: For compound (+)-**3d** one phenyl group and one methoxy group and for compound (+)-**14d** three methoxy groups, two parts of the tetracyclic ester unit, one hydroxymethyl group and two 1,4-dioxane molecules were found disordered over two positions in the asymmetric unit. Several restraints (SADI, SAME, ISOR and SIMU) were used in order to improve refinement stability. Additionally, for compounds (+)-**3d** and (+)-**10b** one badly disordered pentane molecule was found in the asymmetrical unit and could not be satisfactorily refined. The program SQUEEZE[7] was therefore used to remove mathematically the effect of the solvent. The quoted formula and derived parameters are not included the squeezed solvent molecule.

X-ray crystal structure analysis of (+)-3d: A colorless plate-like specimen of $C_{34}H_{30}N_2O_9$, approximate dimensions 0.044 mm x 0.229 mm x 0.401 mm, was used for the X-ray crystallographic analysis. The X-ray intensity data were measured. A total of 1201 frames were collected. The total exposure time was 19.15 hours. The frames were integrated with the Bruker SAINT software package using a wide-frame algorithm. The integration of the data using an orthorhombic unit cell yielded a total of 29734 reflections to a maximum θ angle of 66.59° (0.84 Å resolution), of which 5592 were independent (average redundancy 5.317, completeness = 99.6%, R_{int} = 9.51%, R_{sig} = 6.93%) and 4473 (79.99%) were greater than 2σ(F^2). The final cell constants of a = 6.9473(2) Å, b = 12.8930(3) Å, c = 35.5401(9) Å, volume = 3183.38(14) Å^3, are based upon the refinement of the XYZ-centroids of 9916 reflections above 20 σ(I) with 4.972° < 2θ < 136.2°. Data were corrected for absorption effects using the multi-scan method (SADABS). The ratio of minimum to maximum apparent transmission was 0.813. The calculated minimum and maximum transmission coefficients (based on

crystal size) are 0.7470 and 0.9670. The structure was solved and refined using the Bruker SHELXTL Software Package, using the space group $P2_12_12_1$, with Z = 4 for the formula unit, $C_{34}H_{30}N_2O_9$. The final anisotropic full-matrix least-squares refinement on F^2 with 451 variables converged at R1 = 5.64%, for the observed data and wR2 = 12.79% for all data. The goodness-of-fit was 1.039. The largest peak in the final difference electron density synthesis was 0.267 $e^-/Å^3$ and the largest hole was -0.208 $e^-/Å^3$ with an RMS deviation of 0.050 $e^-/Å^3$. On the basis of the final model, the calculated density was 1.274 g/cm^3 and F(000), 1280 e^-. The hydrogen at N5 atom was refined freely. Flack parameter was refined to 0.07(18). CCDC number: 1951235.

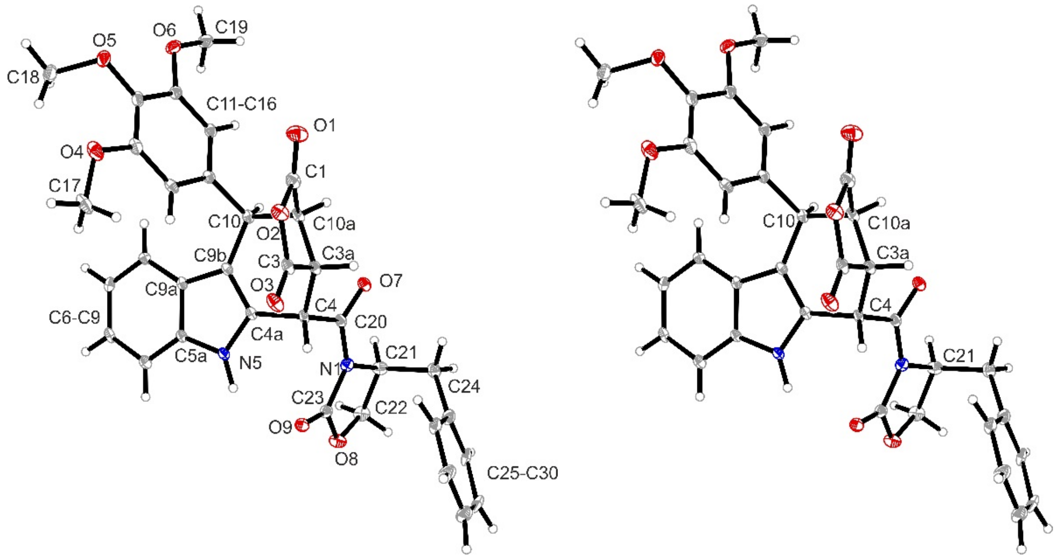

Figure S1: Crystal structure of compound (+)-**3d**. Thermal ellipsoids are set at 20 % probability.

X-ray crystal structure analysis of (+)-10b: A colorless needle-like specimen of $C_{35}H_{33}N_3O_8$, approximate dimensions 0.101 mm x 0.116 mm x 0.394 mm, was used for the X-ray crystallographic analysis. The X-ray intensity data were measured. The integration of the data using a hexagonal unit cell yielded a total of 69694 reflections to a maximum θ angle of 68.45° (0.83 Å resolution), of which 7810 were independent (average redundancy 8.924, completeness = 99.8%, R_{int} = 10.59%, R_{sig} = 5.15%) and 6923 (88.64%) were greater than 2σ(F^2). The final cell constants of a = 25.2233(6) Å, b = 25.2233(6) Å, c = 11.5676(4) Å, volume = 6373.5(4) Å^3, are based upon the refinement of the XYZ-centroids of 66 reflections above 20 σ(I) with 4.060° < 2θ < 41.13°. Data were corrected for absorption effects using the multi-scan method

(SADABS). The calculated minimum and maximum transmission coefficients (based on crystal size) are 0.8050 and 0.9440. The structure was solved and refined using the Bruker SHELXTL Software Package, using the space group *P*6₅, with Z = 6 for the formula unit, $C_{35}H_{33}N_3O_8$. The final anisotropic full-matrix least-squares refinement on F^2 with 422 variables converged at R1 = 4.96%, for the observed data and wR2 = 10.97% for all data. The goodness-of-fit was 1.035. The largest peak in the final difference electron density synthesis was 0.226 $e^-/Å^3$ and the largest hole was -0.201 $e^-/Å^3$ with an RMS deviation of 0.039 $e^-/Å^3$. On the basis of the final model, the calculated density was 0.975 g/cm^3 and F(000), 1968 e^-. The hydrogen at N5 atom was refined freely, but with fixed U-value und DFIX restraints. Flack parameter was refined to 0.04(9). CCDC number: 1951236.

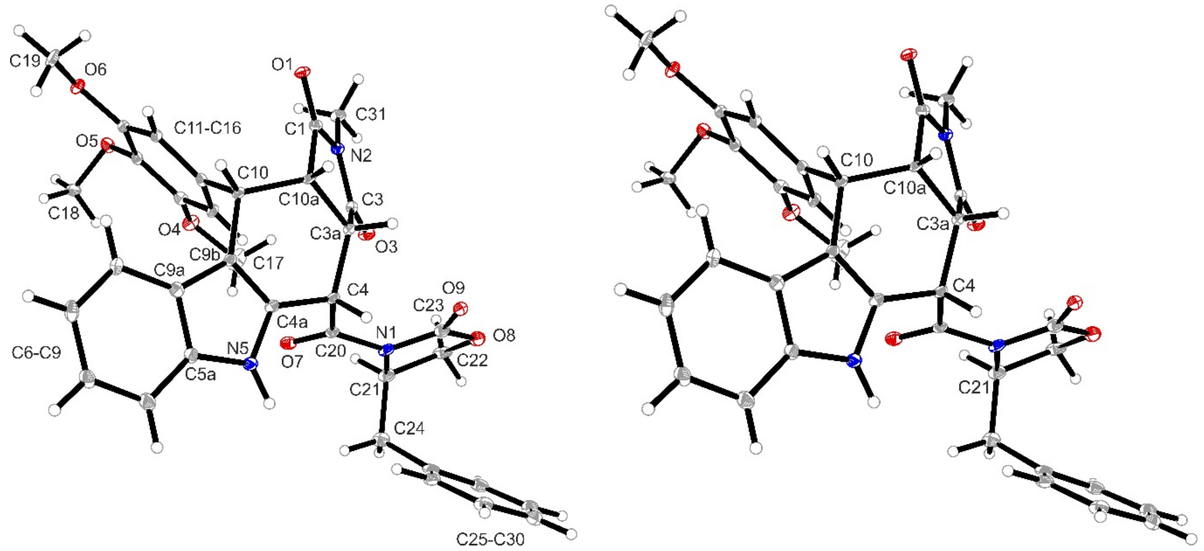

Figure S2: Crystal structure of compound (+)-**10b**. Thermal ellipsoids are set at 20 % probability.

X-ray crystal structure analysis of (+)-14d: A colorless prism-like specimen of $C_{41}H_{49}N_3O_{11}$, approximate dimensions 0.111 mm x 0.170 mm x 0.203 mm, was used for the X-ray crystallographic analysis. The X-ray intensity data were measured. A total of 1010 frames were collected. The total exposure time was 14.99 hours. The frames were integrated with the Bruker SAINT software package using a wide-frame algorithm. The integration of the data using a monoclinic unit cell yielded a total of 27137 reflections to a maximum θ angle of 68.29° (0.83 Å resolution), of which 7122 were independent (average redundancy 3.810, completeness = 99.5%, R_{int} = 5.33%, R_{sig} = 4.43%) and 6127 (86.03%) were greater than $2\sigma(F^2)$. The final cell constants of a = 13.7527(4) Å, b = 9.6664(3) Å, c = 16.0175(4) Å, β = 112.8830(10)°, volume =

1961.77(10) Å^3, are based upon the refinement of the XYZ-centroids of 9903 reflections above 20 σ(I) with 5.989° < 2θ < 136.2°. Data were corrected for absorption effects using the multi-scan method (SADABS). The ratio of minimum to maximum apparent transmission was 0.931. The calculated minimum and maximum transmission coefficients (based on crystal size) are 0.8590 and 0.9190. The structure was solved and refined using the Bruker SHELXTL Software Package, using the space group *P*2$_1$, with Z = 2 for the formula unit, $C_{41}H_{49}N_3O_{11}$. The final anisotropic full-matrix least-squares refinement on F^2 with 792 variables converged at R1 = 5.84%, for the observed data and wR2 = 16.29% for all data. The goodness-of-fit was 1.037. The largest peak in the final difference electron density synthesis was 0.466 e$^-$/Å^3 and the largest hole was -0.196 e$^-$/Å^3 with an RMS deviation of 0.045 e$^-$/Å^3. On the basis of the final model, the calculated density was 1.286 g/cm^3 and F(000), 808 e$^-$. The hydrogen at N1 and N5 atoms were refined freely, but with fixed U-value und DFIX restraints. Flack parameter was refined to 0.02(9). CCDC number: 1951237.

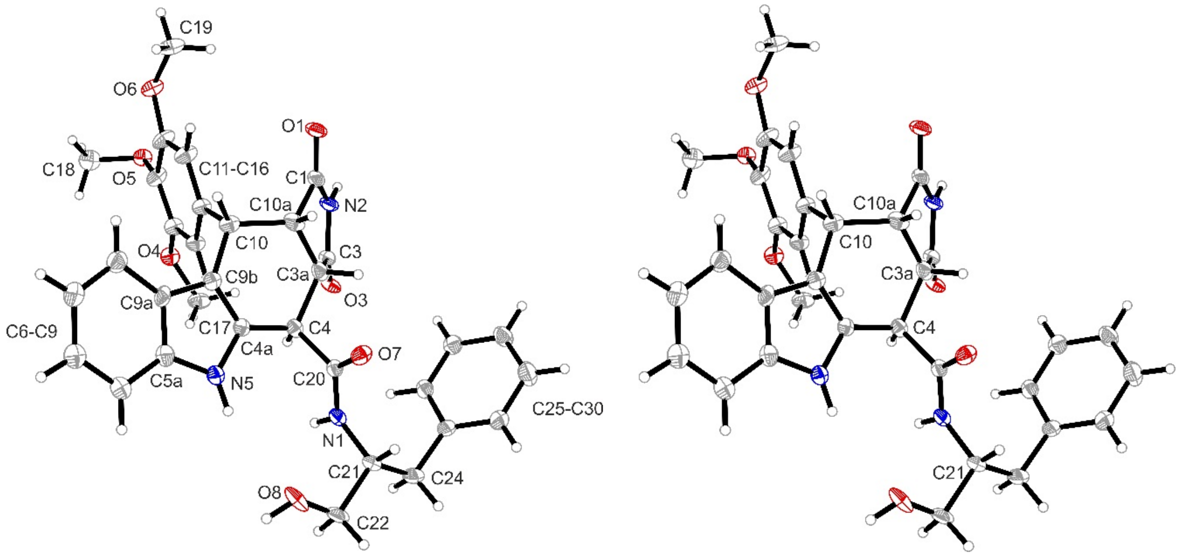

Figure S3: Crystal structure of compound (+)-**14d**. Thermal ellipsoids are set at 20 % probability.

7. Inhibition of the CK2α/CK2β interaction analyzed by MST

Table S4: Dissociation constants K_D and K_D' for the $CK2\alpha^{1-335}/CK2\beta^{1-193}$ interaction in the absence and presence of potential inhibitors (I), respectively, obtained by MST.[a]

[I] = 0	K_D = 12 ± 1 nM (*n* = 4)			K_D = 20 ± 9 nM (*n* = 3)		
compound	[µM]	*n*	K_D' [µM]	[µM]	*n*	K_D' [µM]
(+)-**3a** (W16)	100	3	61 ± 17*			n. d.
(-)-**3a**	50	4	174 ± 51*	20	3	17 ± 11 (n. s.)
(+)-**3c**			prec.			n. d.
(+)-**3d**	100	3	38.1 ± 4.2**			n. d.
(+)-**9a**	50	5	8 ± 2 (n. s.)	10	2	19 ± 12 (n. s.)
(+)-**9a**				20	2	26 ± 17 (n. s.)
(-)-**9a**	100	3	332 ± 52***			n. d.
(+)-**9b**	100	4	248 ± 30***	20	3	46 ± 21 (n. s.)
(-)-**9b**	100	3	261 ± 15****	20	4	9 ± 3 (n. s.)
(+)-**9c**			n. d.			prec.
(-)-**9c**			n. d.			prec.
(+)-**9d**			n. d.	20	3	25 ± 13 (n. s.)
(-)-**9d**			n. d.	20	4	16 ± 3 (n. s.)
(+)-**10a**	100	4	524 ± 132**	20	3	5 ± 3 (n. s.)
(-)-**10a**	100	3	171 ± 47*			prec.
(+)-**10b**	100	4	186 ± 36**	20	5	72 ± 29 (n. s.)
(-)-**10b**	100	3	328 ± 63**	20	3	62 ± 22 (n. s.)
(±)-**11**	100	2	59 ± 25*	20	2	7 ± 1 (n. s.)
(±)-**12**	100	2	758 ± 338*	20	3	58 ± 21 (n. s.)
(+)-**14c**			n. d.	20	3	20 ± 3 (n. s.)
(-)-**14c**			n. d.	20	3	23 ± 3 (n. s.)
(+)-**14d**			n. d.	20	3	26 ± 8 (n. s.)
(-)-**14d**			n. d.	20	3	9 ± 3 (n. s.)

[a] Shown are mean ± SEM values of K_D and K_D' for *n* separate experiments. Values of K_D for different preparations of fluorescently labeled $CK2\beta^{1-193}$ were not significantly different (unpaired Student's t-test, P > 0.05). Values of K_D (column head) and K_D' were compared by an unpaired Student's t-test. Differences are stated as follows: not significant (n. s.), P > 0.05; *, P ≤ 0.05; **, P ≤ 0.01; ***, P ≤ 0.001; ****, P ≤ 0.0001. prec. = precipitation; n. d. = not determined.

8. References

[1] D. Royer, Y.-S. Wong, S. Plé, A. Chiaroni, K. Diker, J. Lévy, J. Diastereodivergence and appendage diversity in the multicomponent synthesis of aryl-pyrrolo-tetrahydrocarbazoles, *Tetrahedron* **2008**, *64*, 9607-9618. https://doi.org/10.1016/j.tet.2008.07.045.

[2] P. A. Wender, A. W. White, Methodology for indole synthesis, *Tetrahedron Lett.* **1981,** *22*, 1475–1478.

[3] APEX3 (**2016**), *SAINT* (**2015**) and *SADABS* (**2015**), Bruker AXS Inc., Madison, Wisconsin, USA.

[49 G. M. Sheldrick, *SHELXT – Integrated space-group and crystal-structure determination*, *Acta Cryst.* **2015**, *A71*, 3-8.

[5] G. M. Sheldrick, *Crystal structure refinement with SHELXL*, *Acta Cryst.*, **2015**, *C71 (1)*, 3-8.

[6] *XP – Interactive molecular graphics, Version 5.1,* Bruker AXS Inc., Madison, Wisconsin, USA, **1998**.

[7] A. L. Spek, Acta Cryst. **2015**, C71, 9-18.)

9. ^{1}H and ^{13}C NMR spectra

^{1}H NMR spectrum of (+)-**3a** in $CDCl_3$.

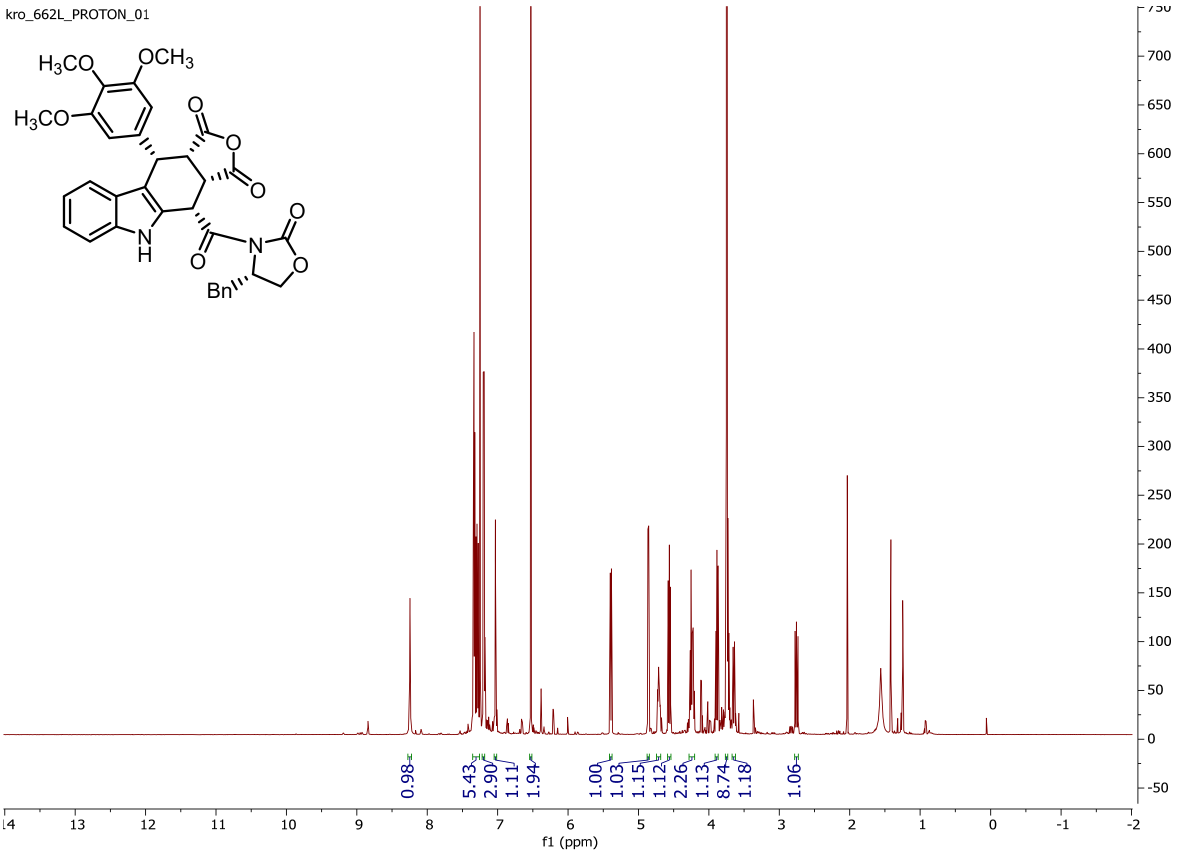

^{13}C NMR spectrum of (+)-**3a** in $CDCl_3$.

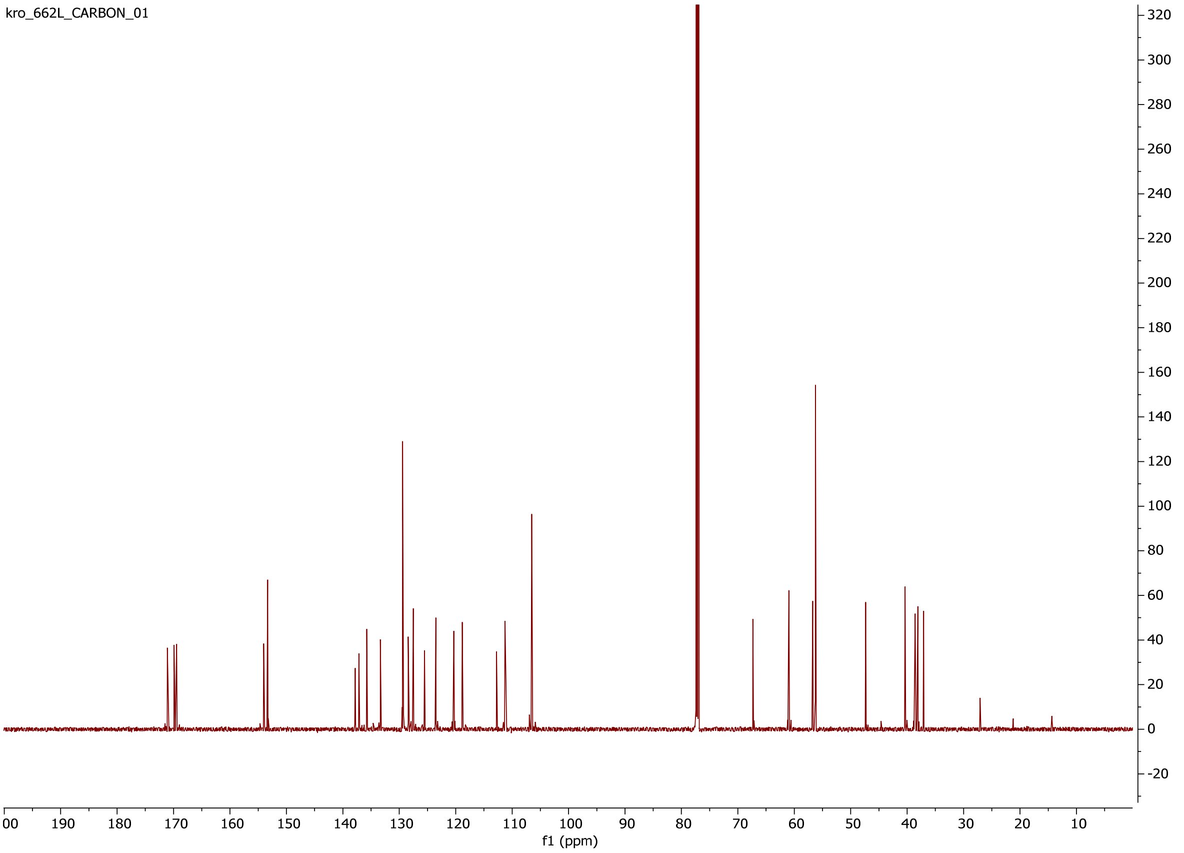

^{1}H NMR spectrum of (+)-**3c** in DMSO-d_6.

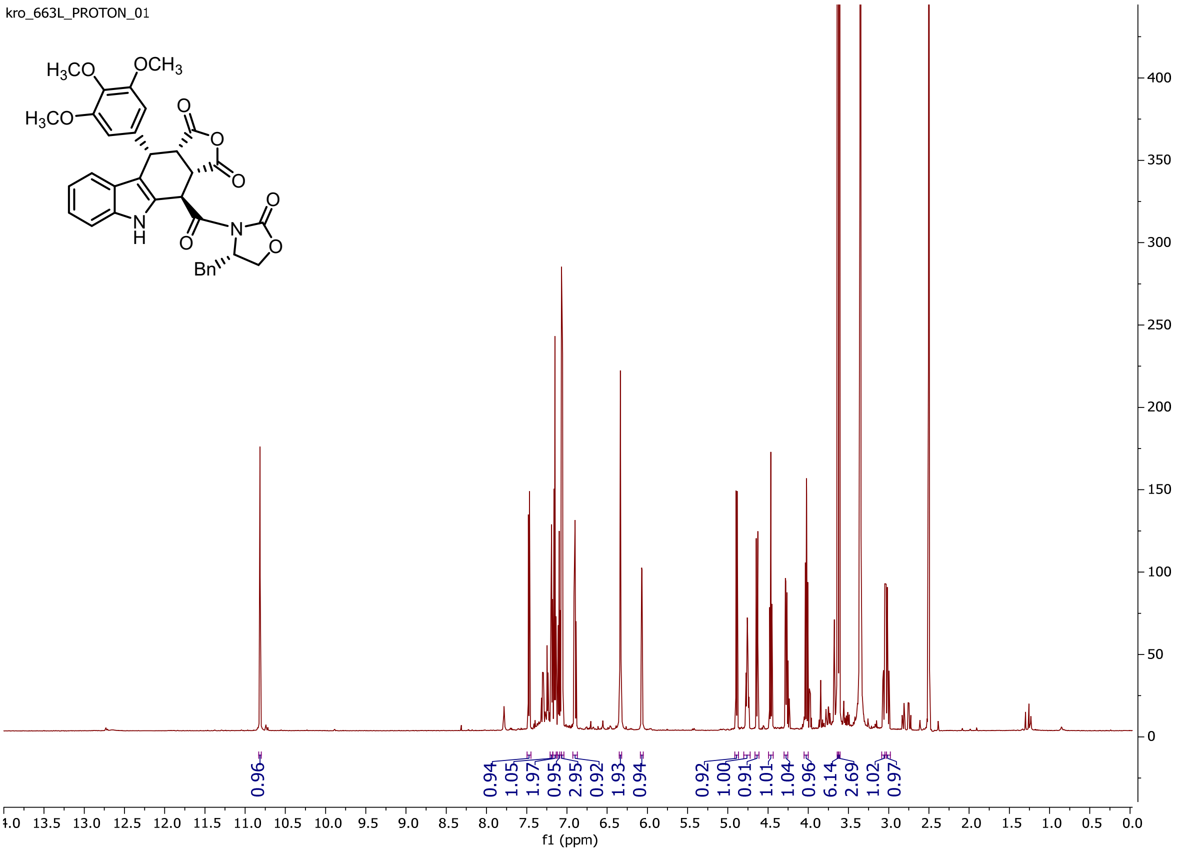

^{13}C NMR spectrum of (+)-**3c** in DMSO-d_6.

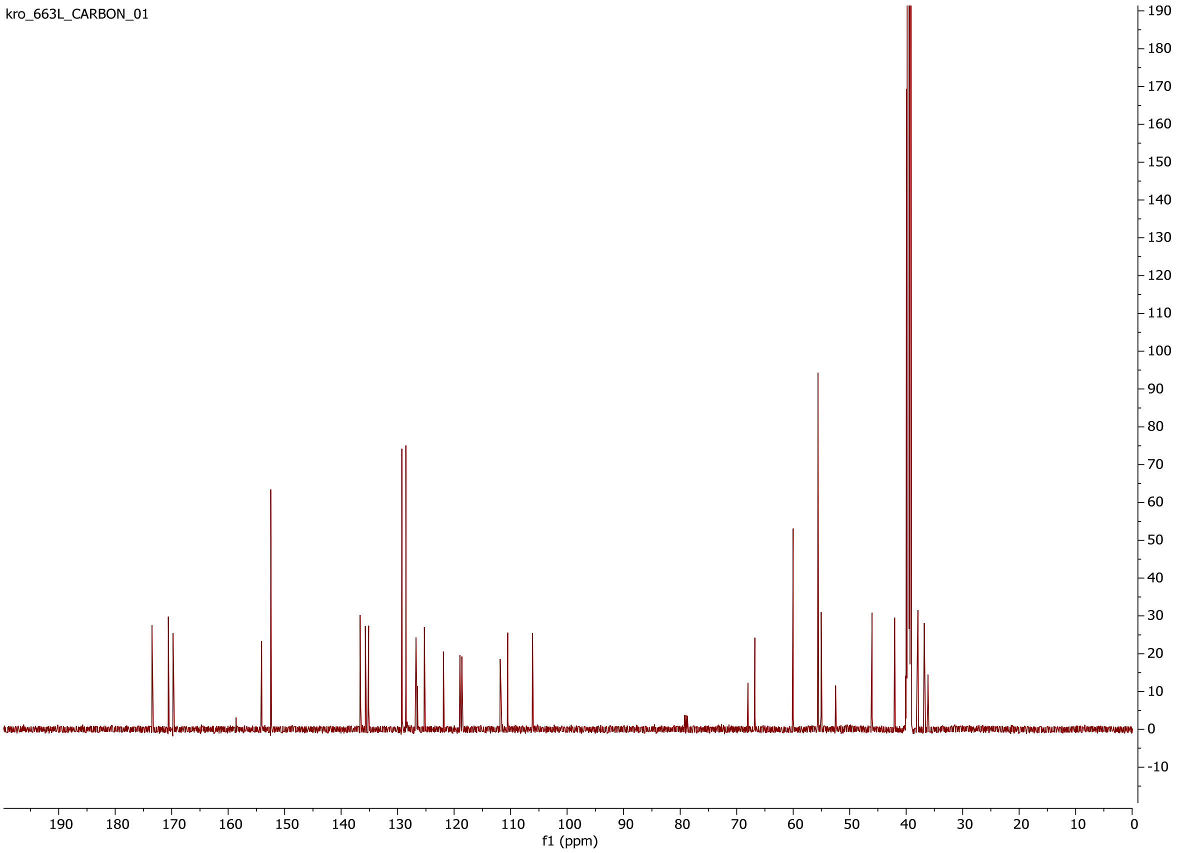

^{1}H NMR spectrum of (+)-**3d** in $CDCl_3$.

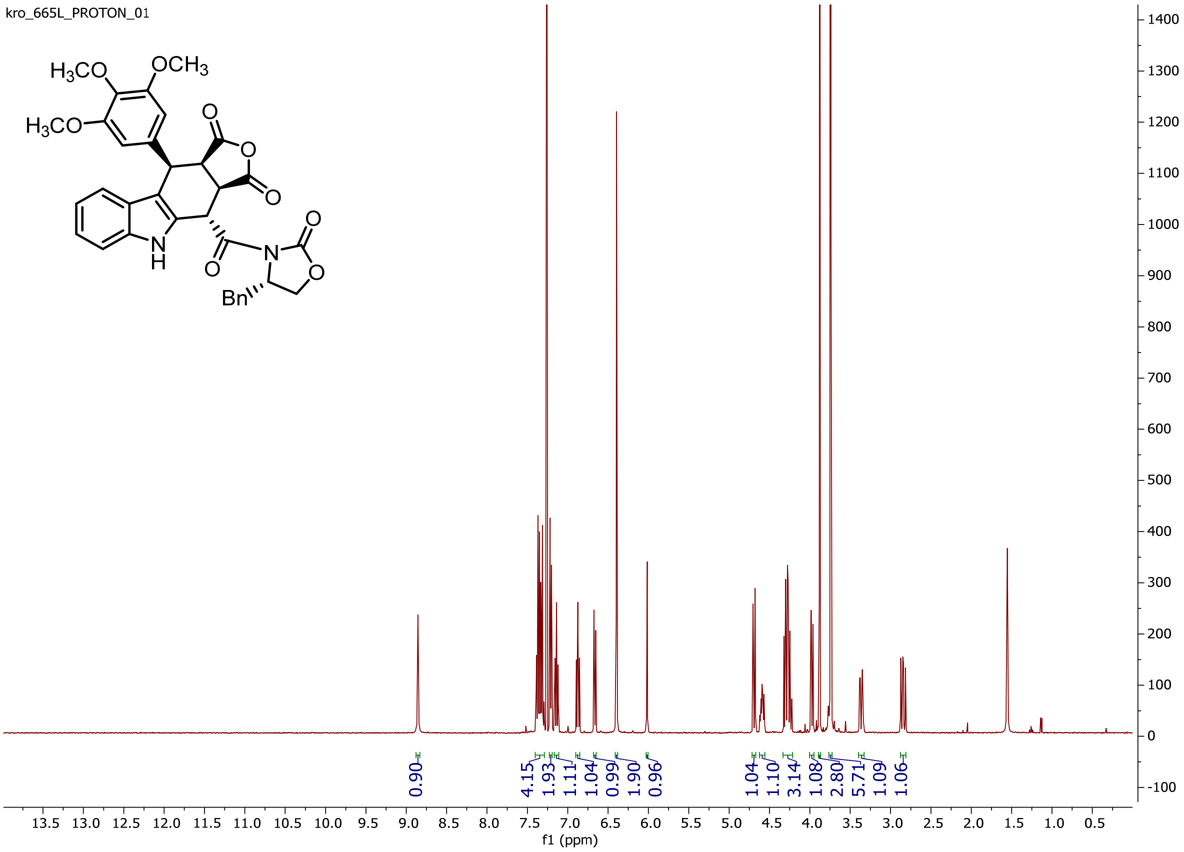

^{13}C NMR spectrum of (+)-**3d** in $CDCl_3$.

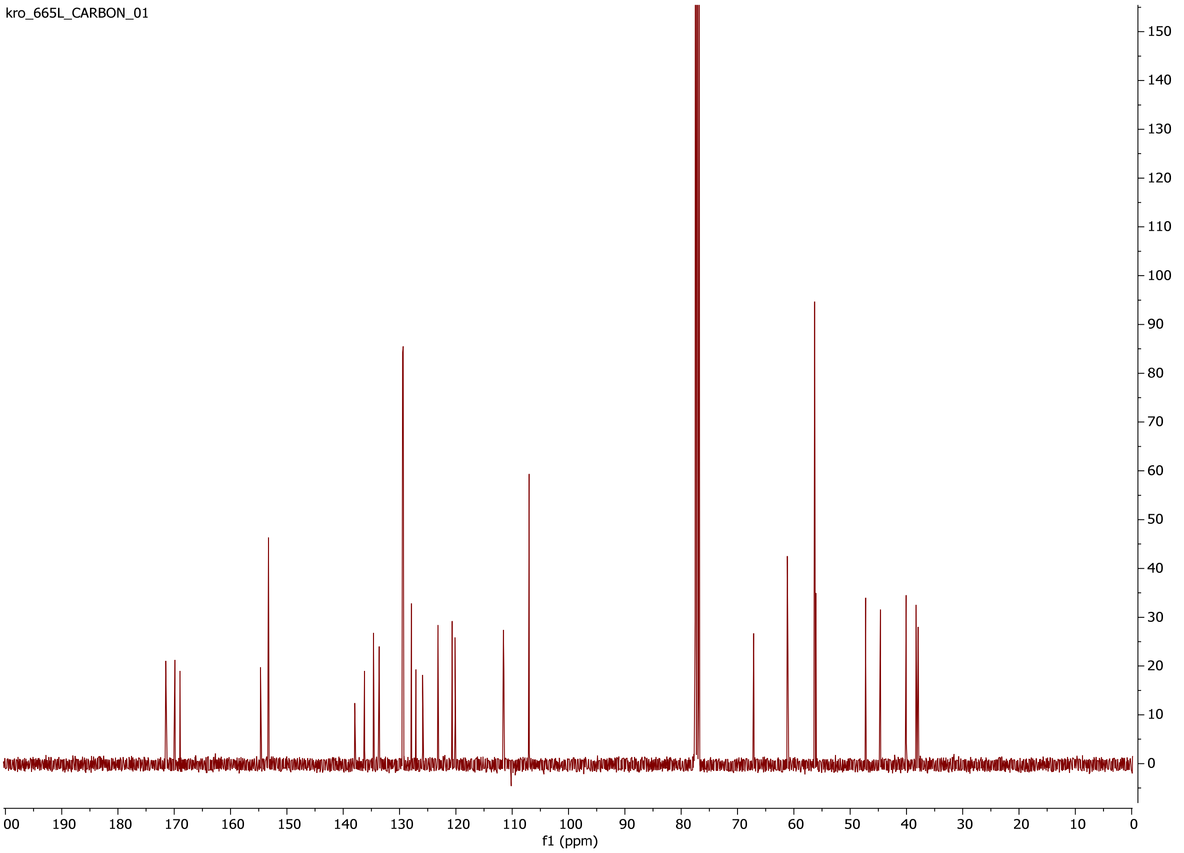

^{1}H NMR spectrum of (-)-**3a** in $CDCl_3$.

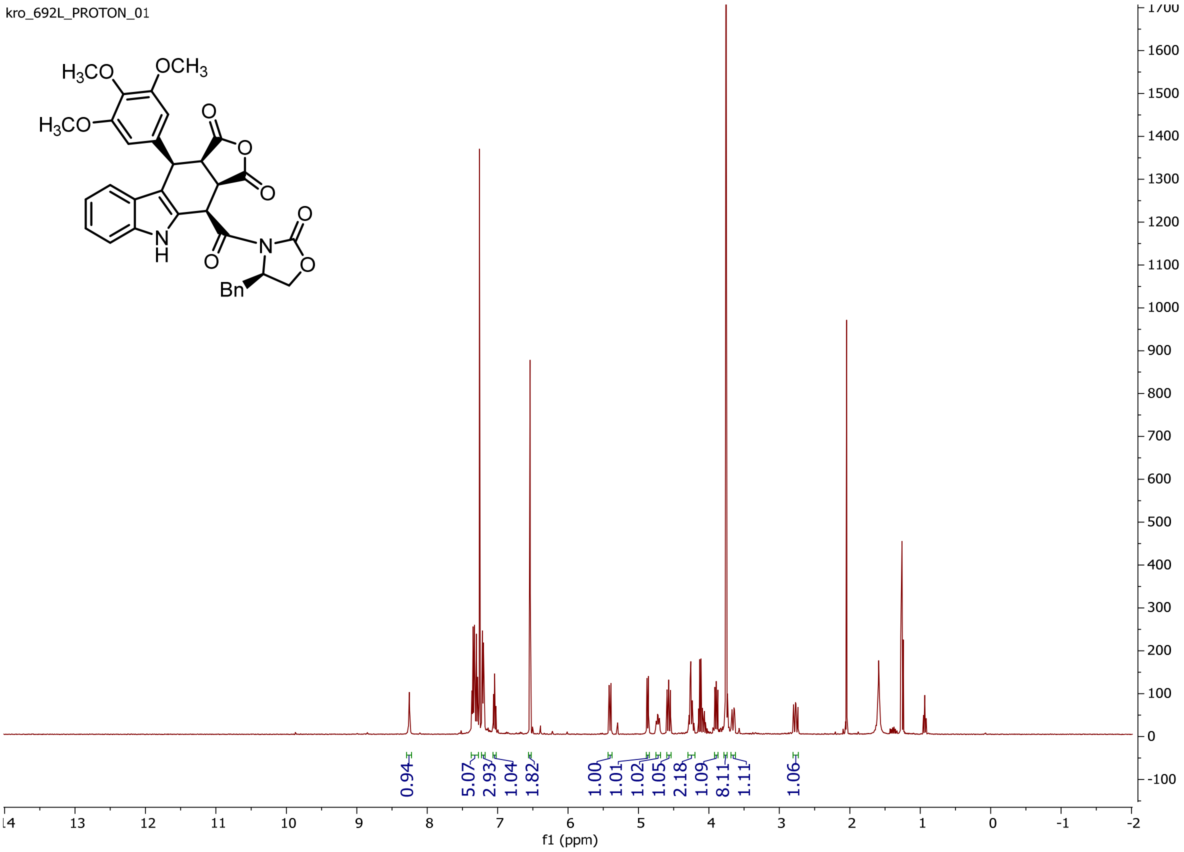

^{13}C NMR spectrum of (-)-**3a** in $CDCl_3$.

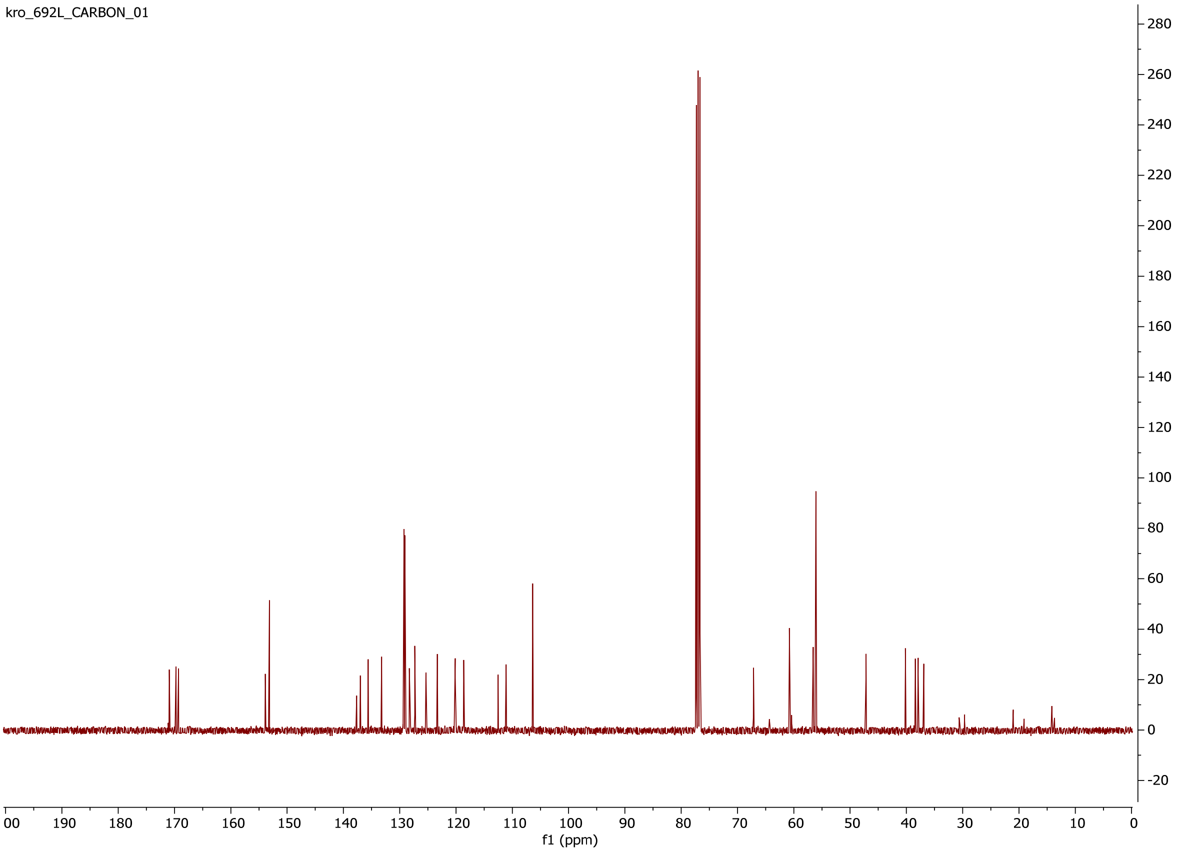

^{1}H NMR spectrum of (-)-**9a** in DMSO-d_6.

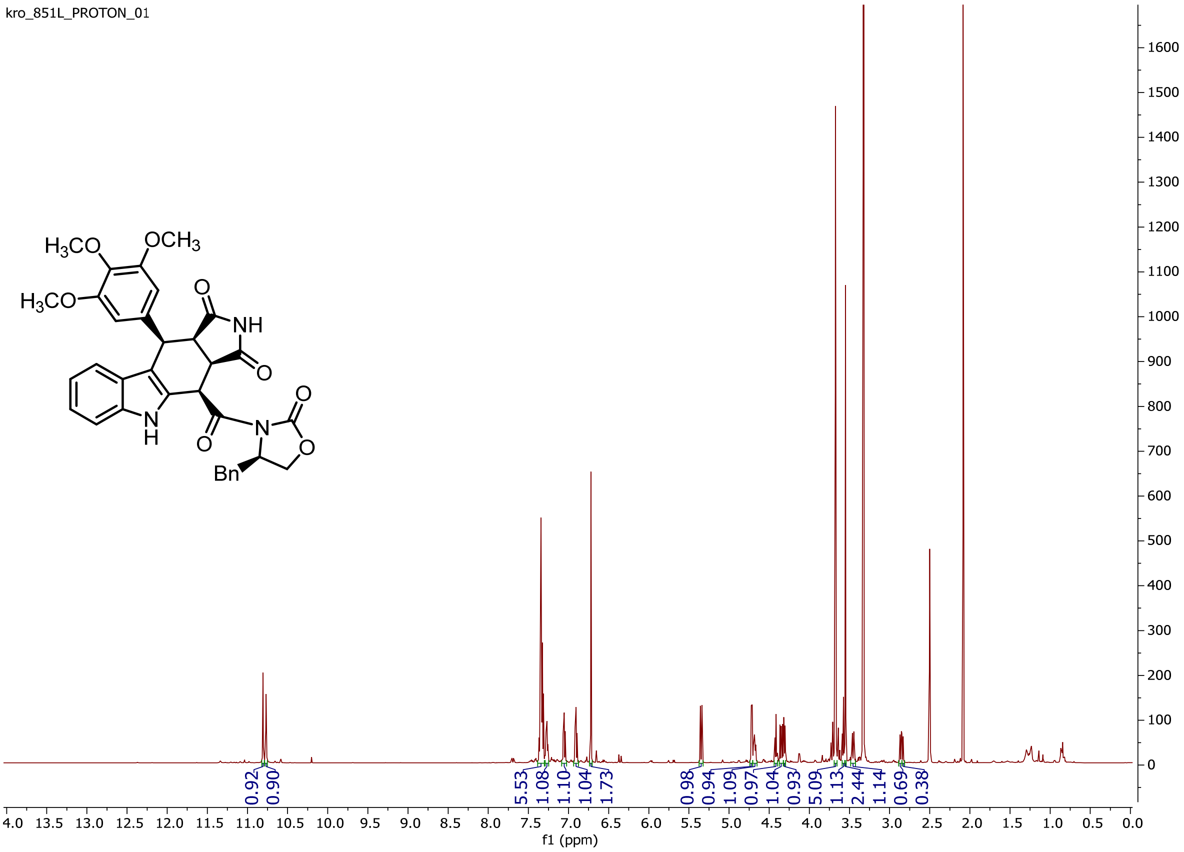

^{13}C NMR spectrum of (-)-**9a** in DMSO-d_6.

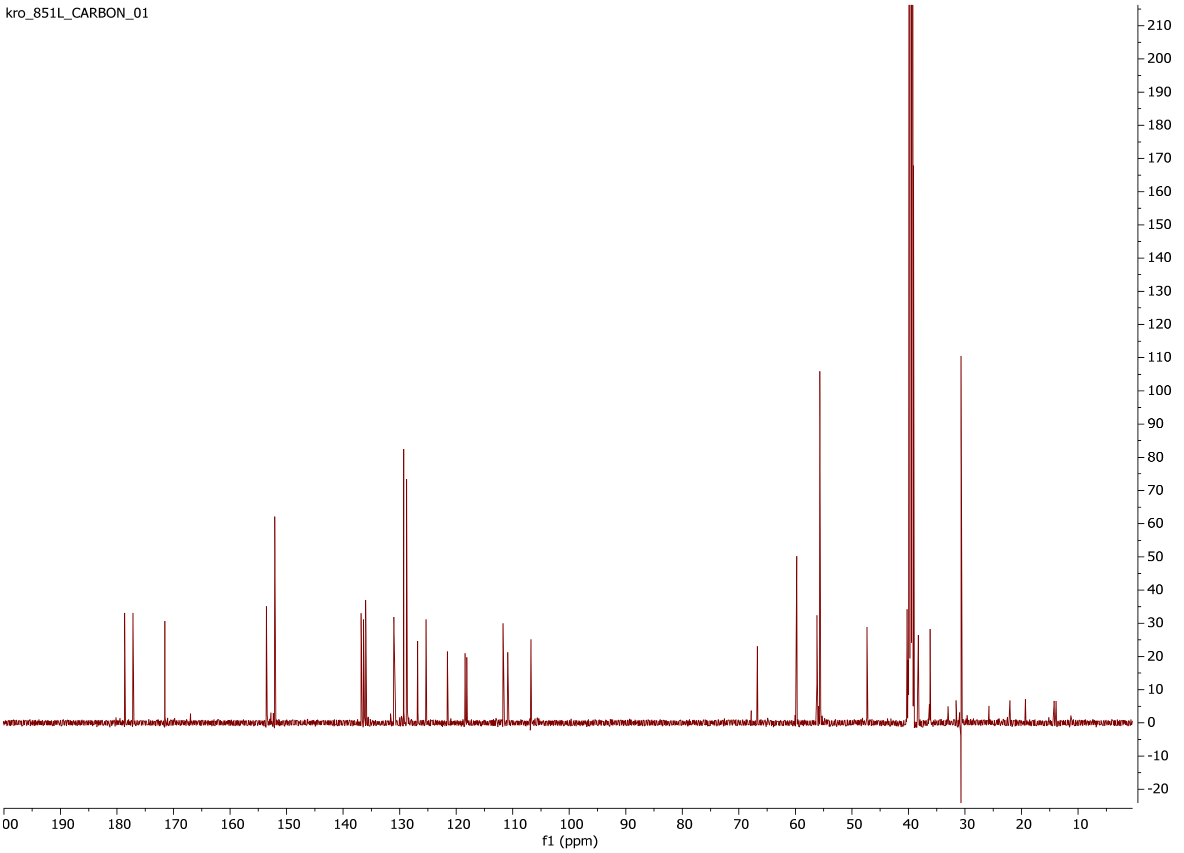

^{1}H NMR spectrum of (-)-**9b** in DMSO-d_6.

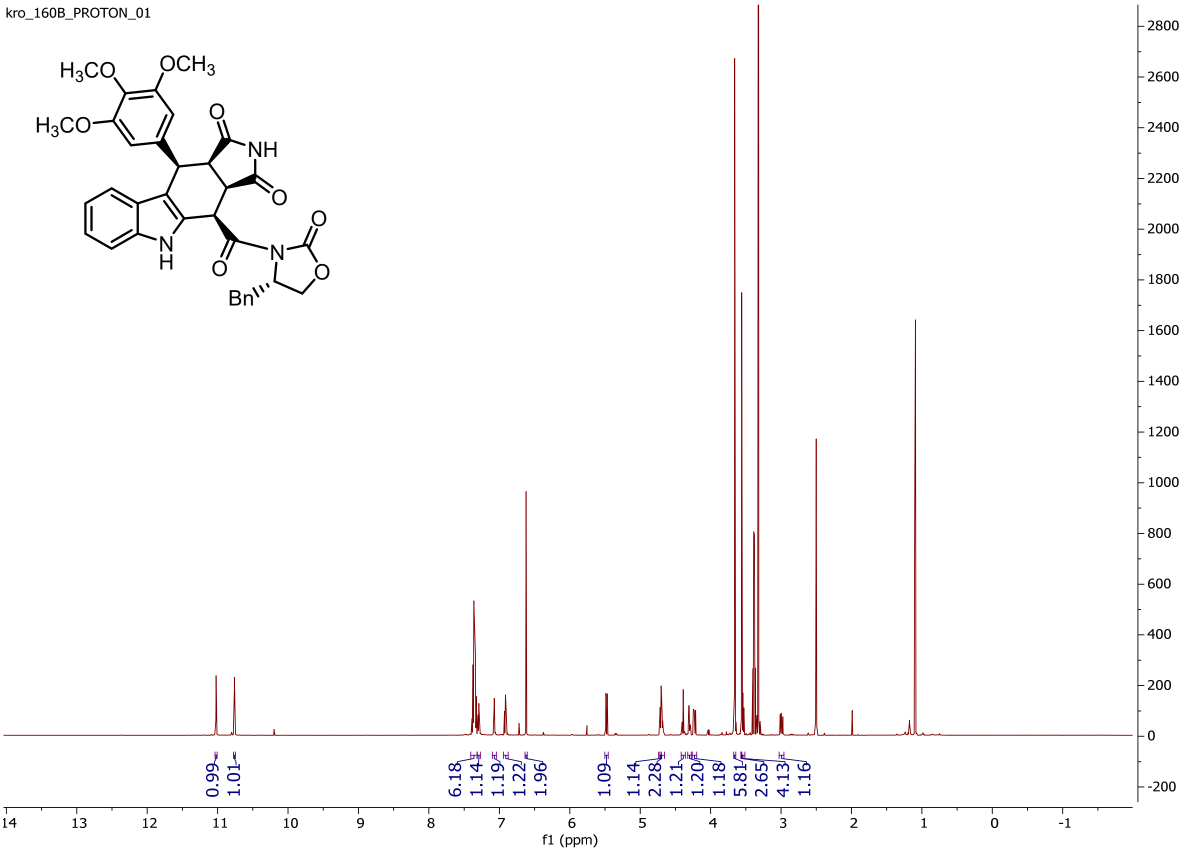

^{13}C NMR spectrum of (-)-**9b** in DMSO-d_6.

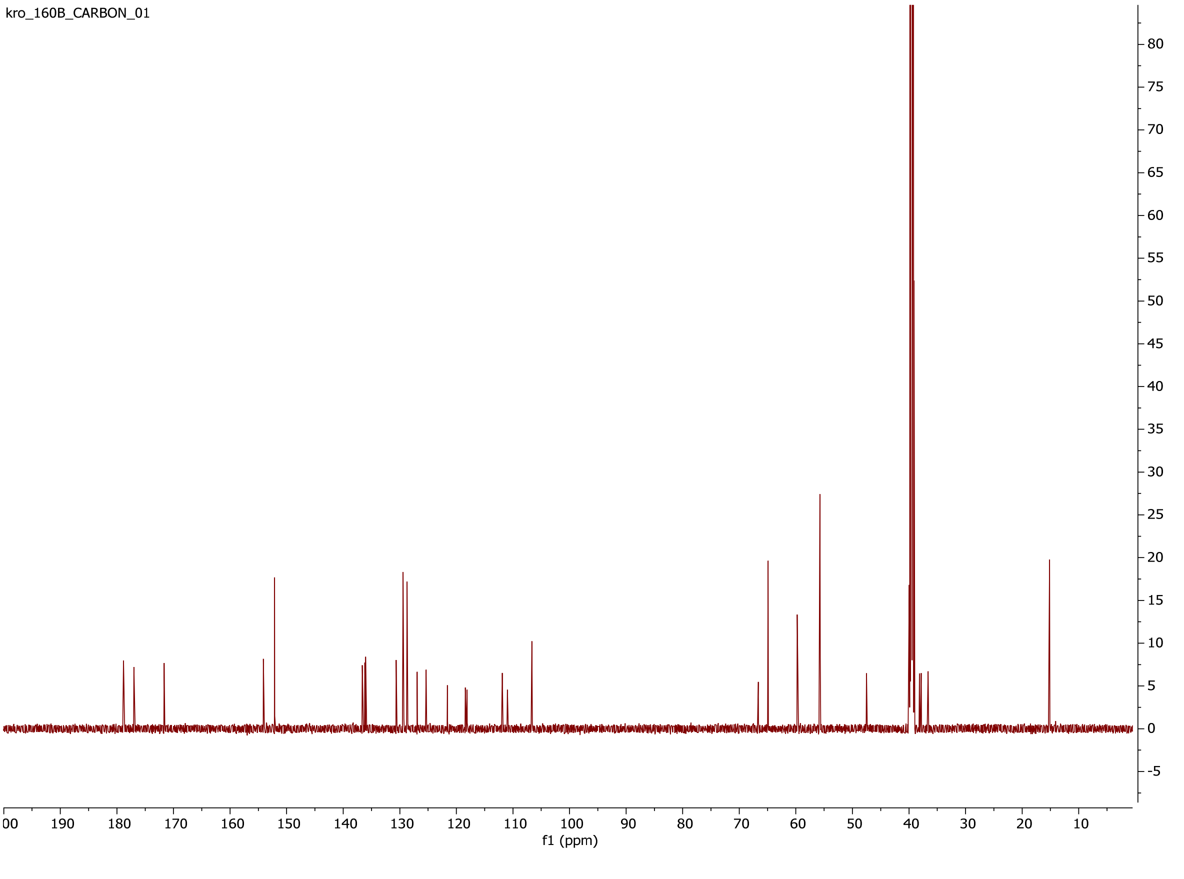

^{1}H NMR spectrum of (-)-**9c** in DMSO-d_6.

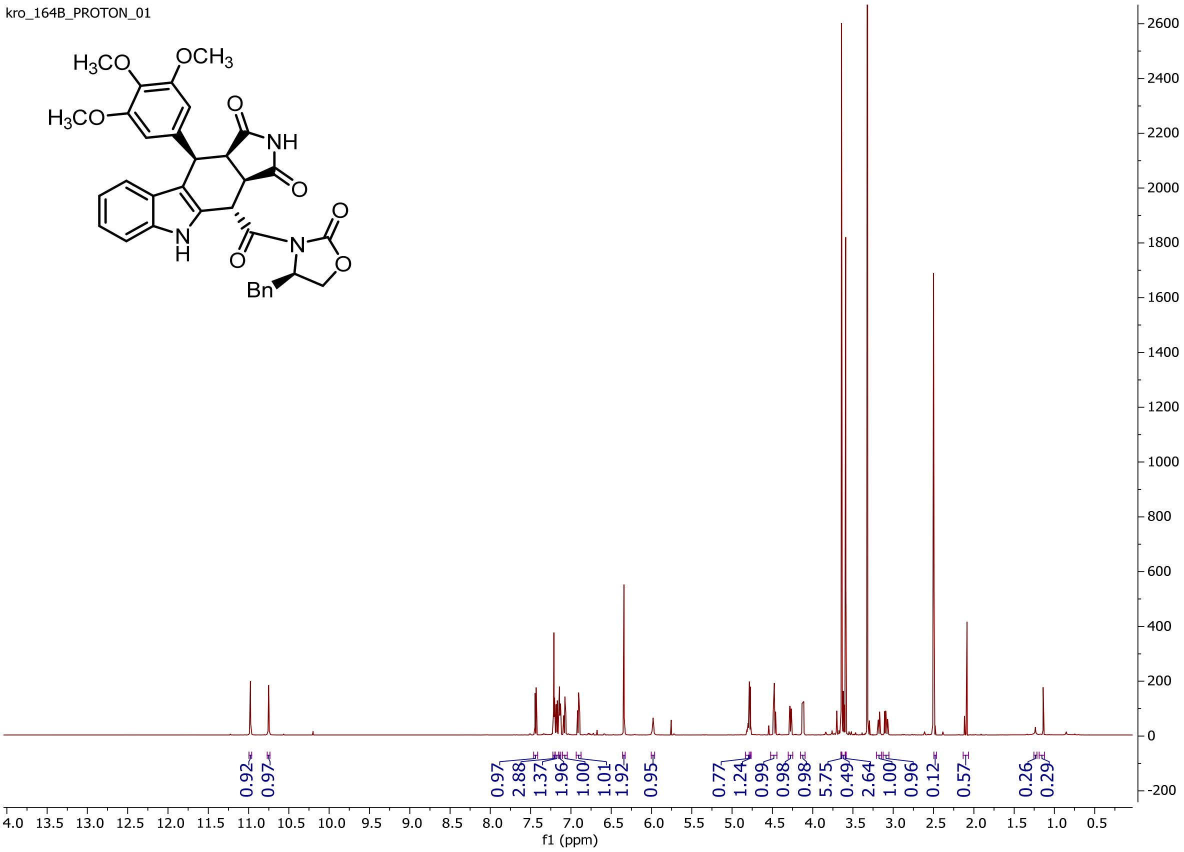

^{13}C NMR spectrum of (-)-**9c** in DMSO-d_6.

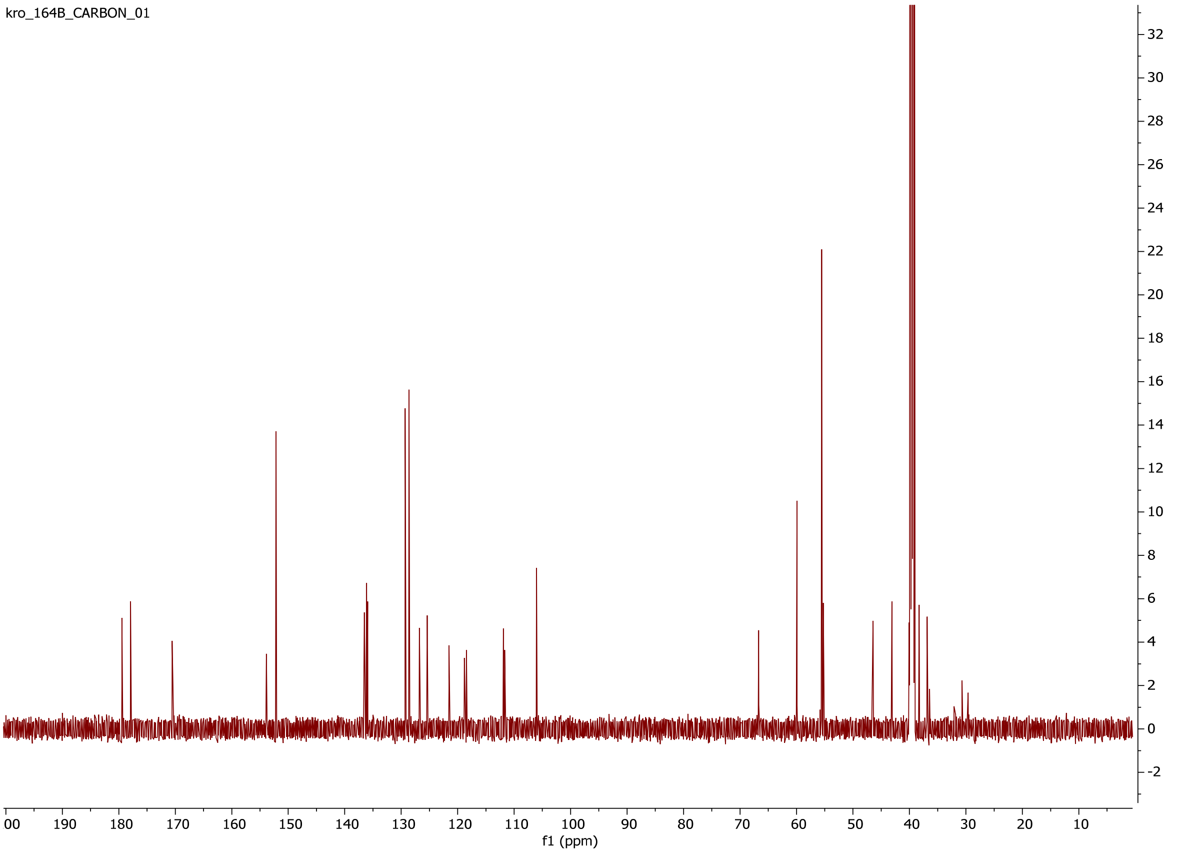

^{1}H NMR spectrum of (-)-**9d** in DMSO-d_6.

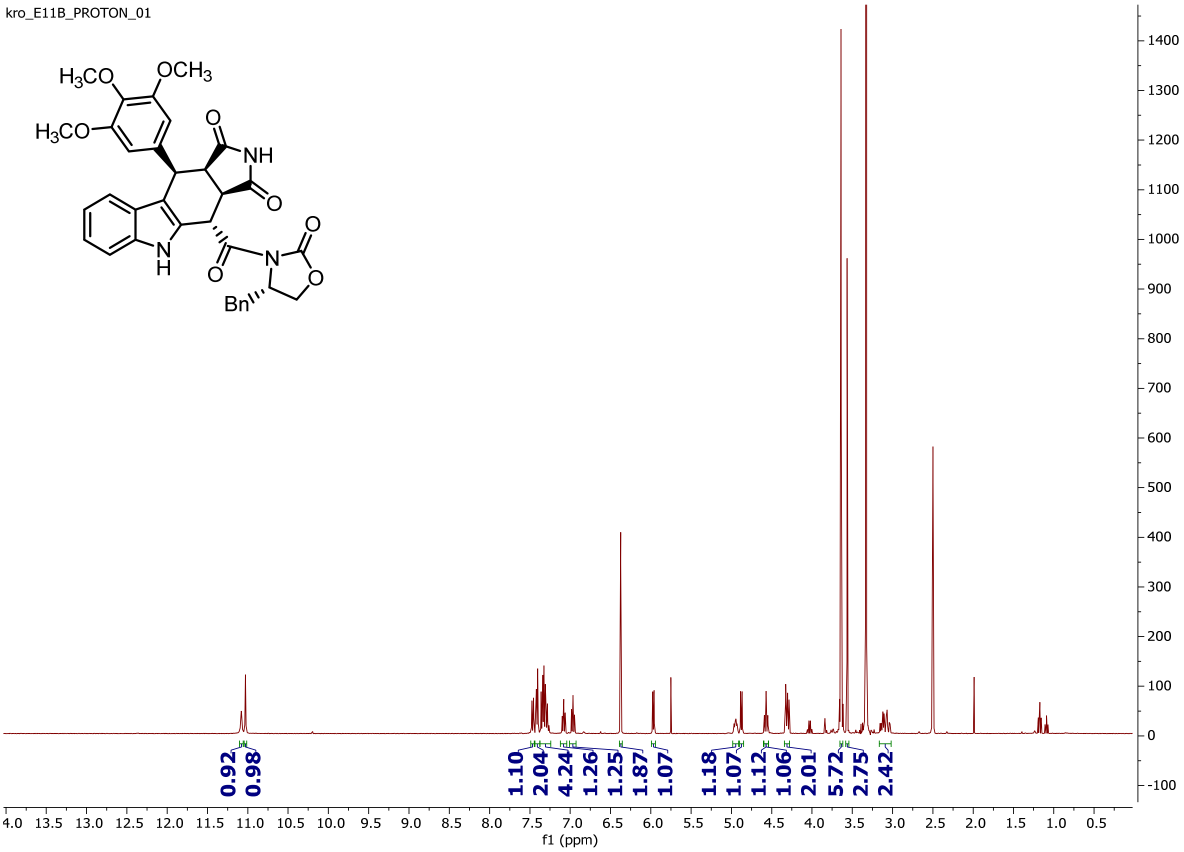

^{13}C NMR spectrum of (-)-**9d** in DMSO-d_6.

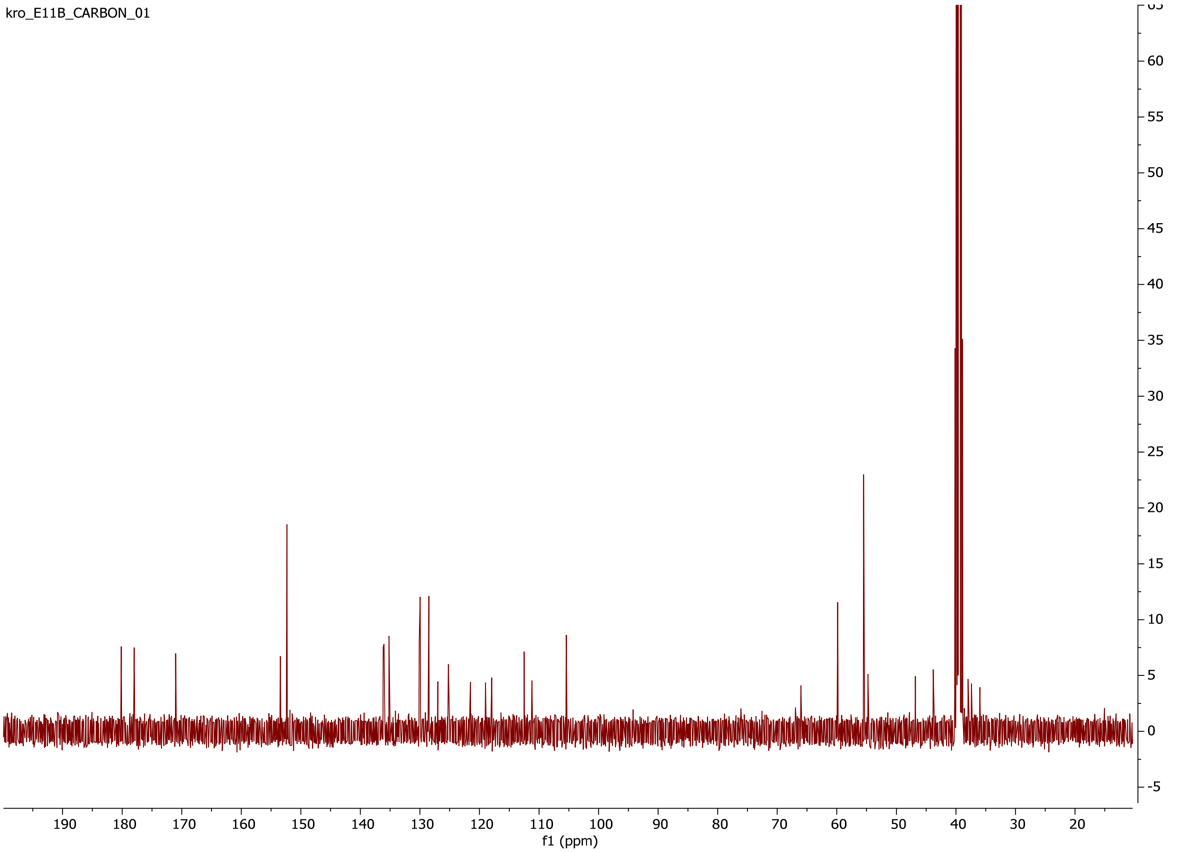

^{1}H NMR spectrum of (+)-**9a** in DMSO-d_6.

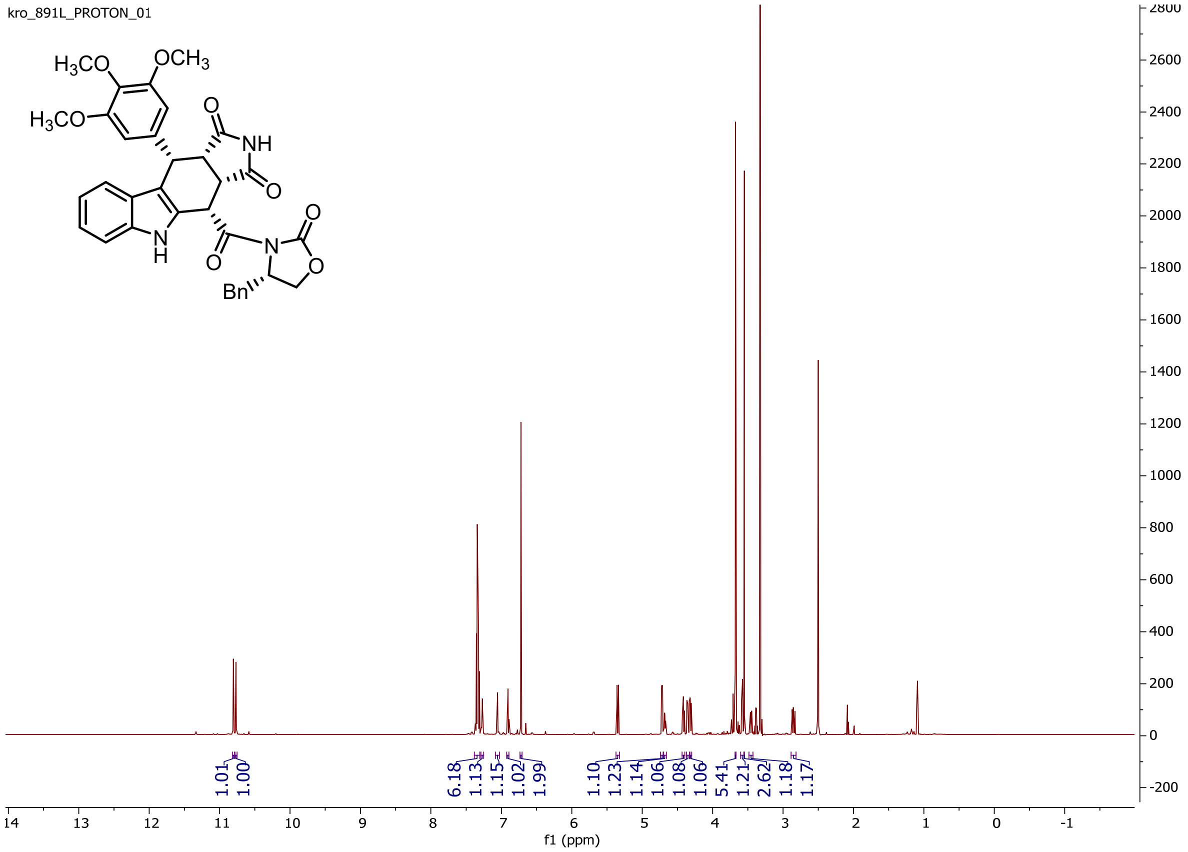

^{13}C NMR spectrum of (+)-**9a** in DMSO-d_6.

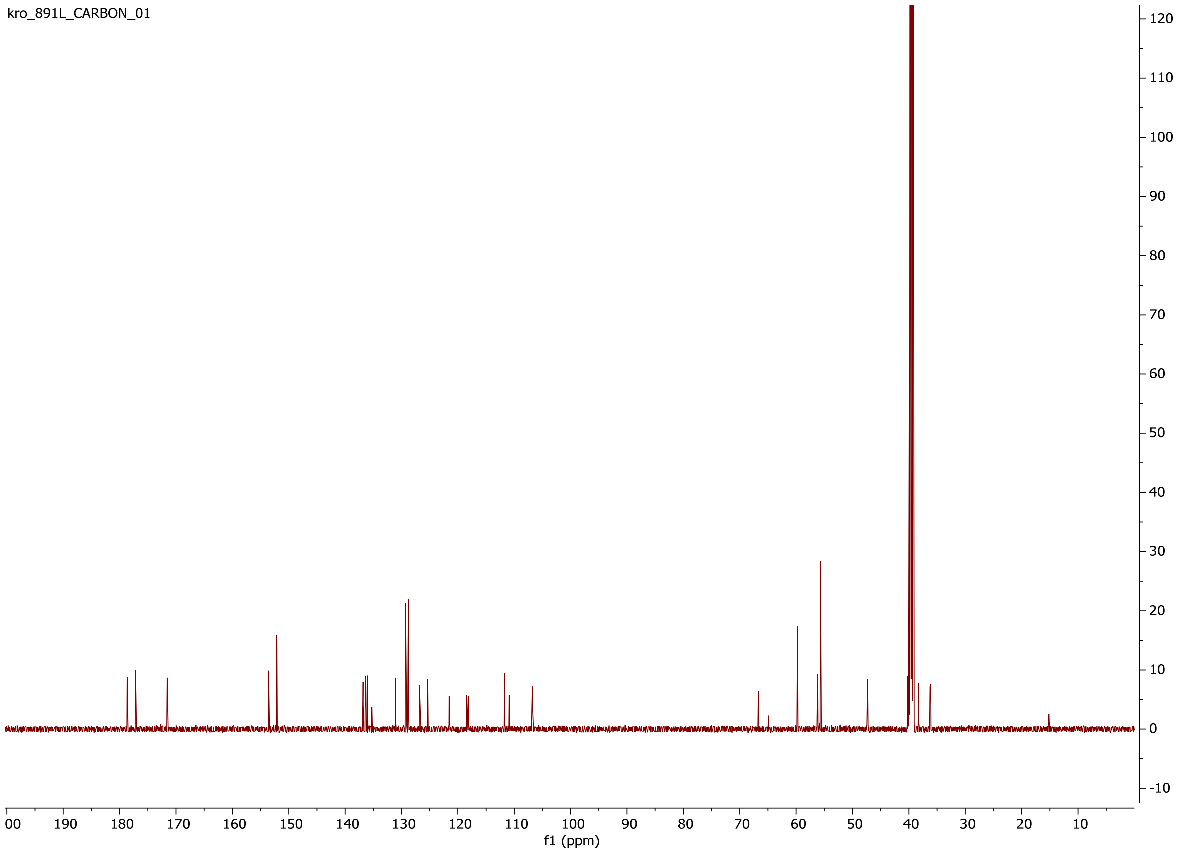

^{j1}H NMR spectrum of (+)-**9b** in DMSO-d_6.

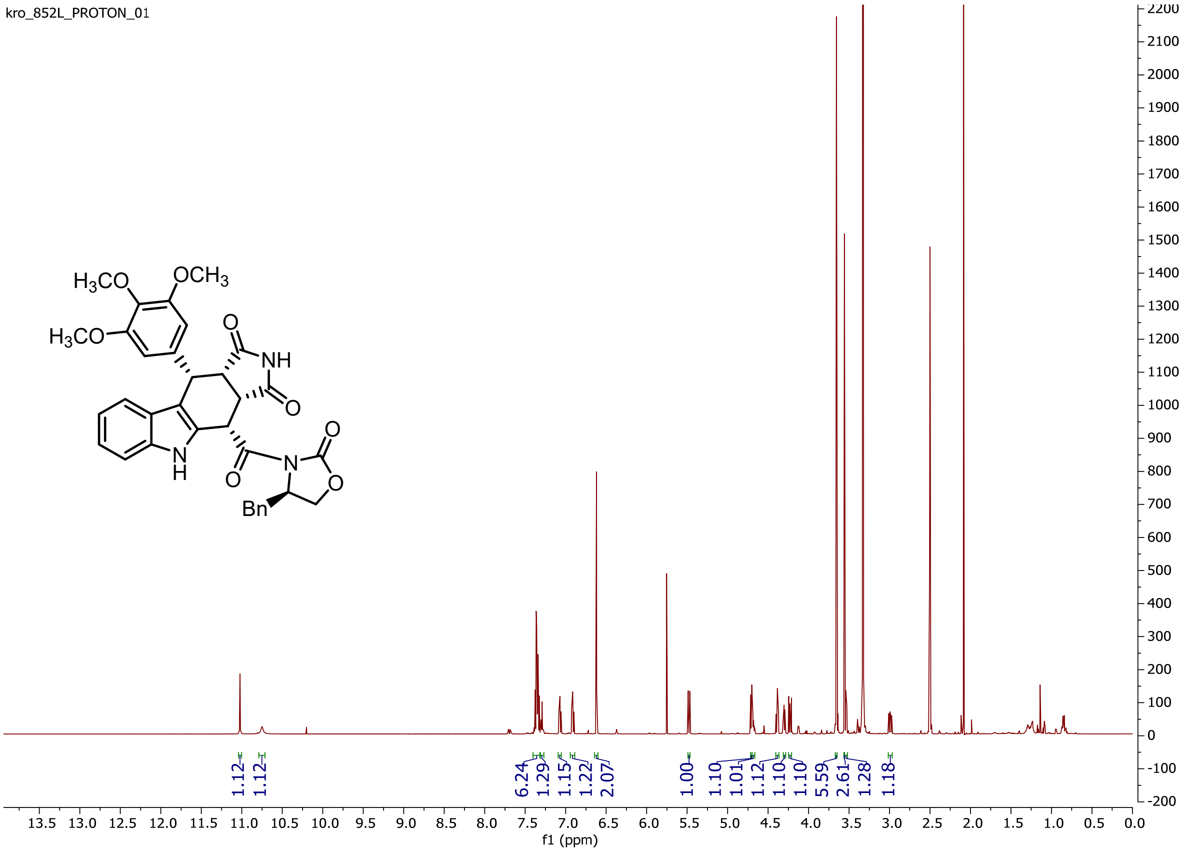

^{13}C NMR spectrum of (+)-**9b** in DMSO-d_6.

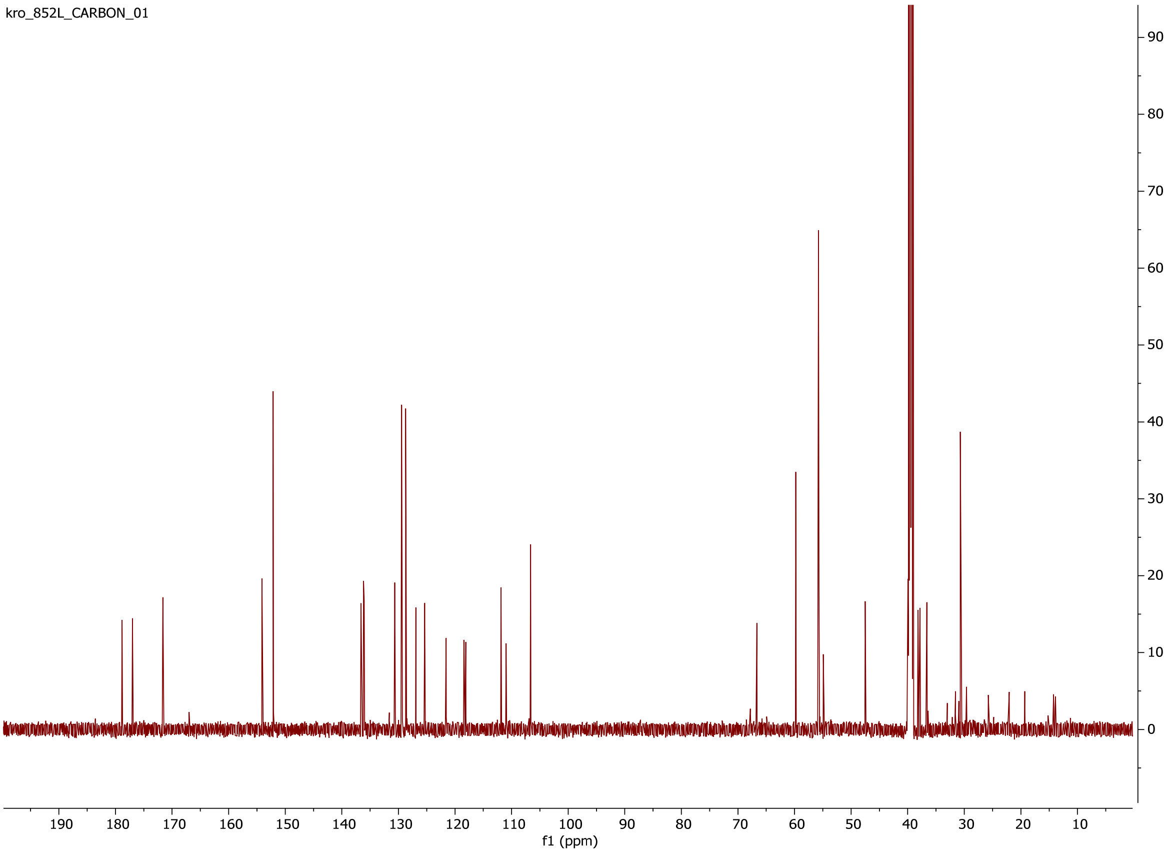

^{1}H NMR spectrum of (+)-**9c** in DMSO-d_6.

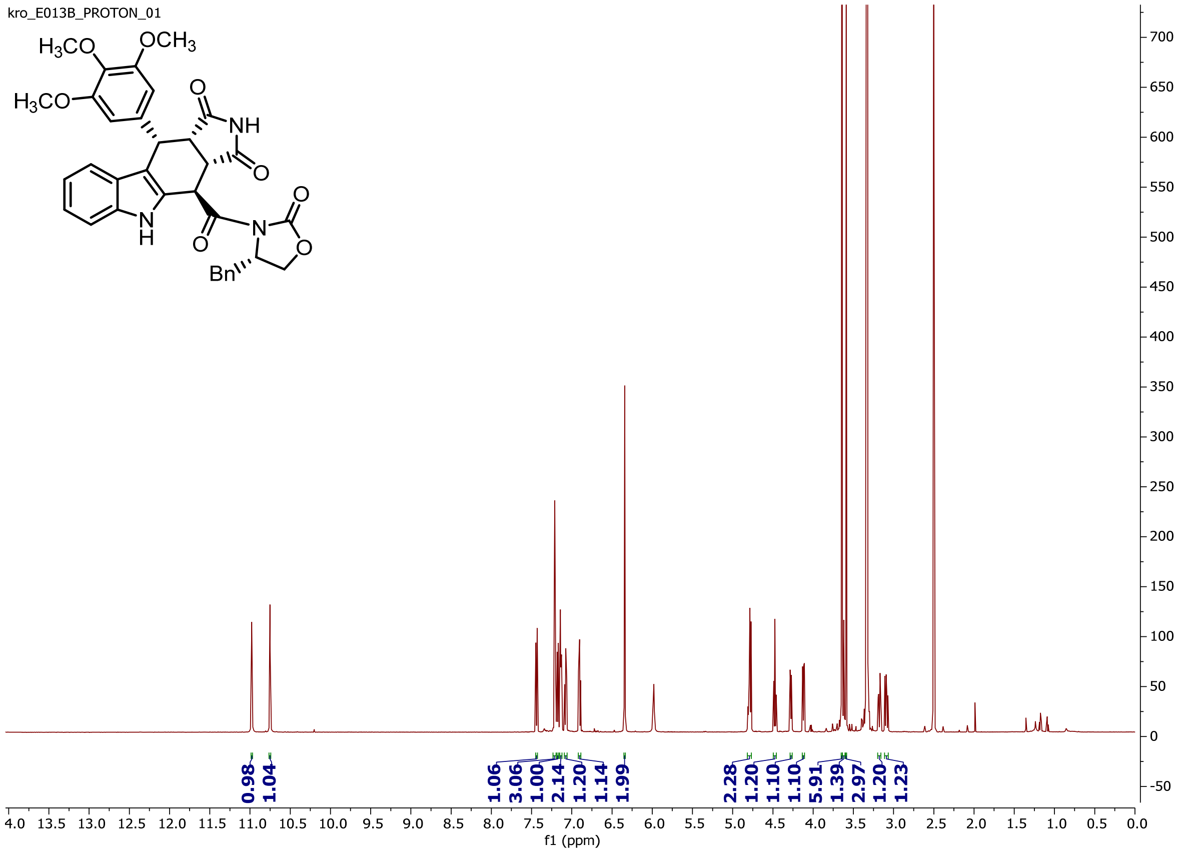

^{13}C NMR spectrum of (+)-**9c** in DMSO-d_6.

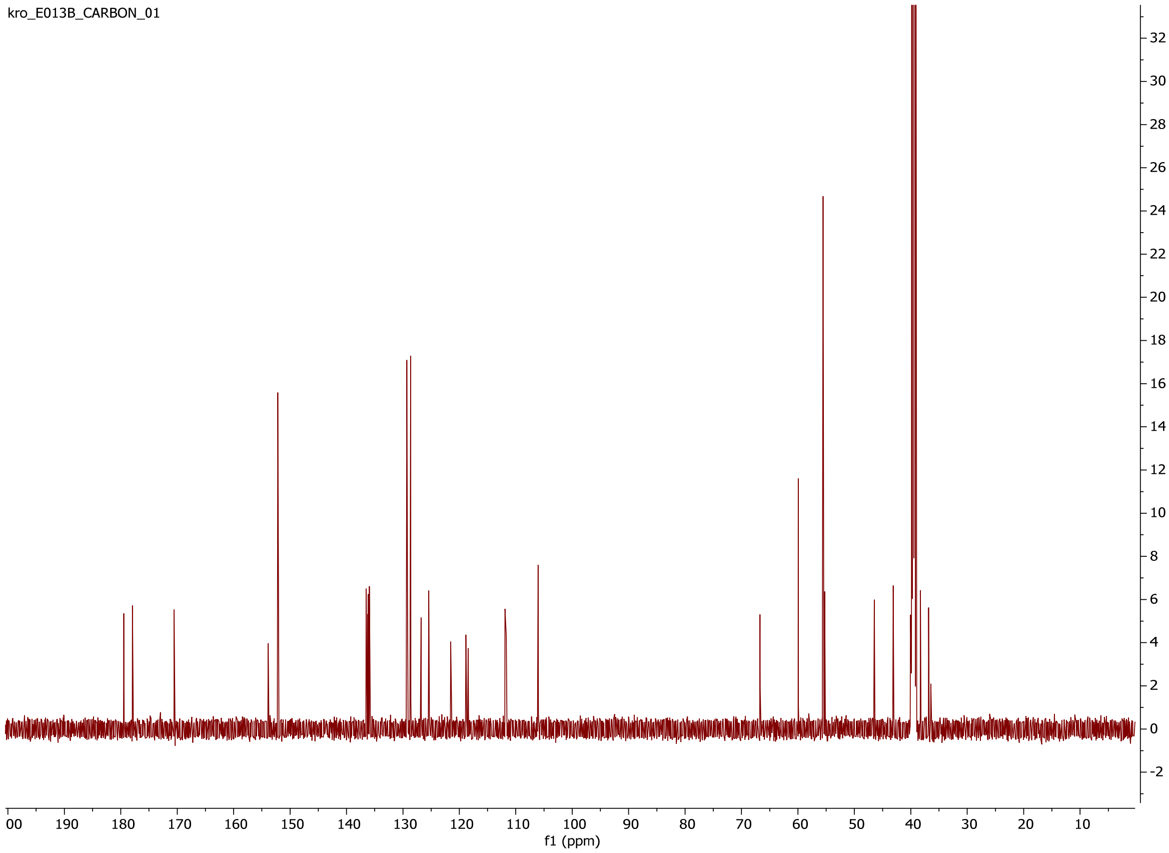

^1H NMR spectrum of (+)-**9d** in DMSO-d_6.

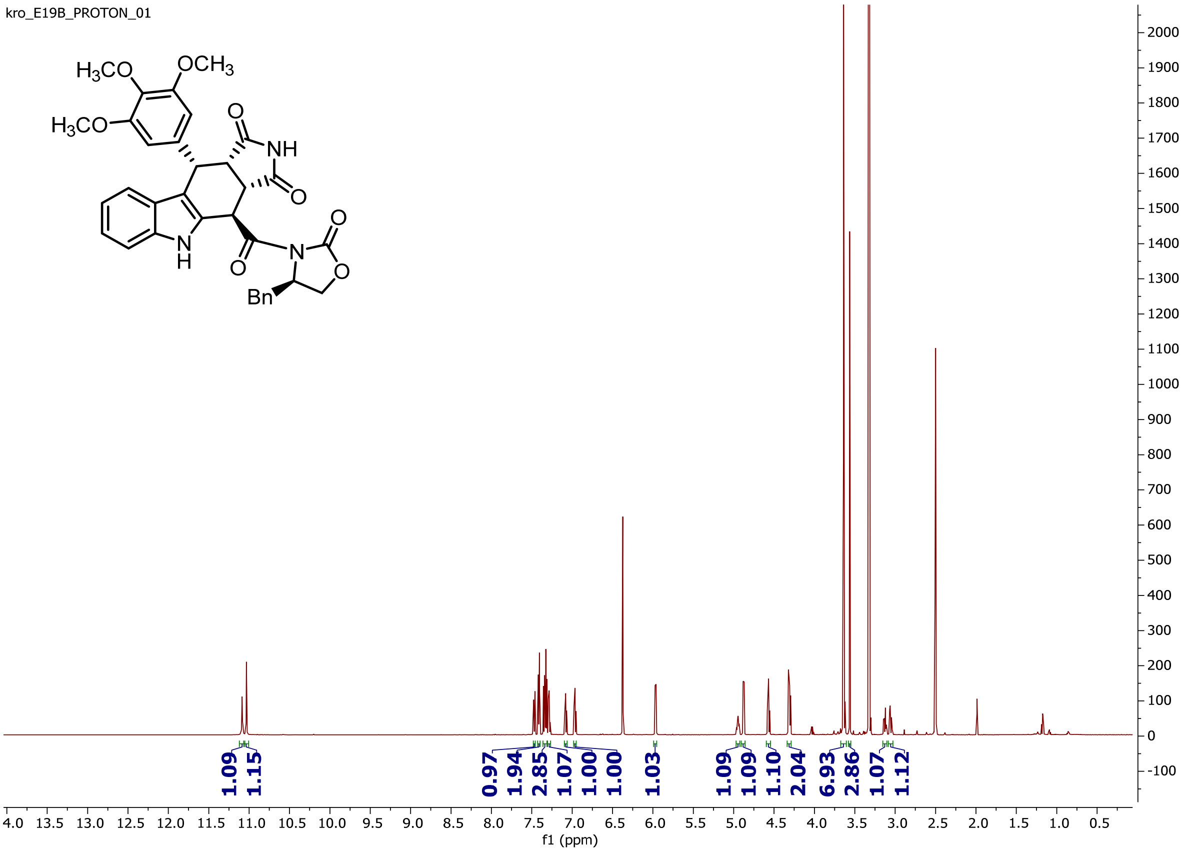

^{13}C NMR spectrum of (+)-**9d** in DMSO-d_6.

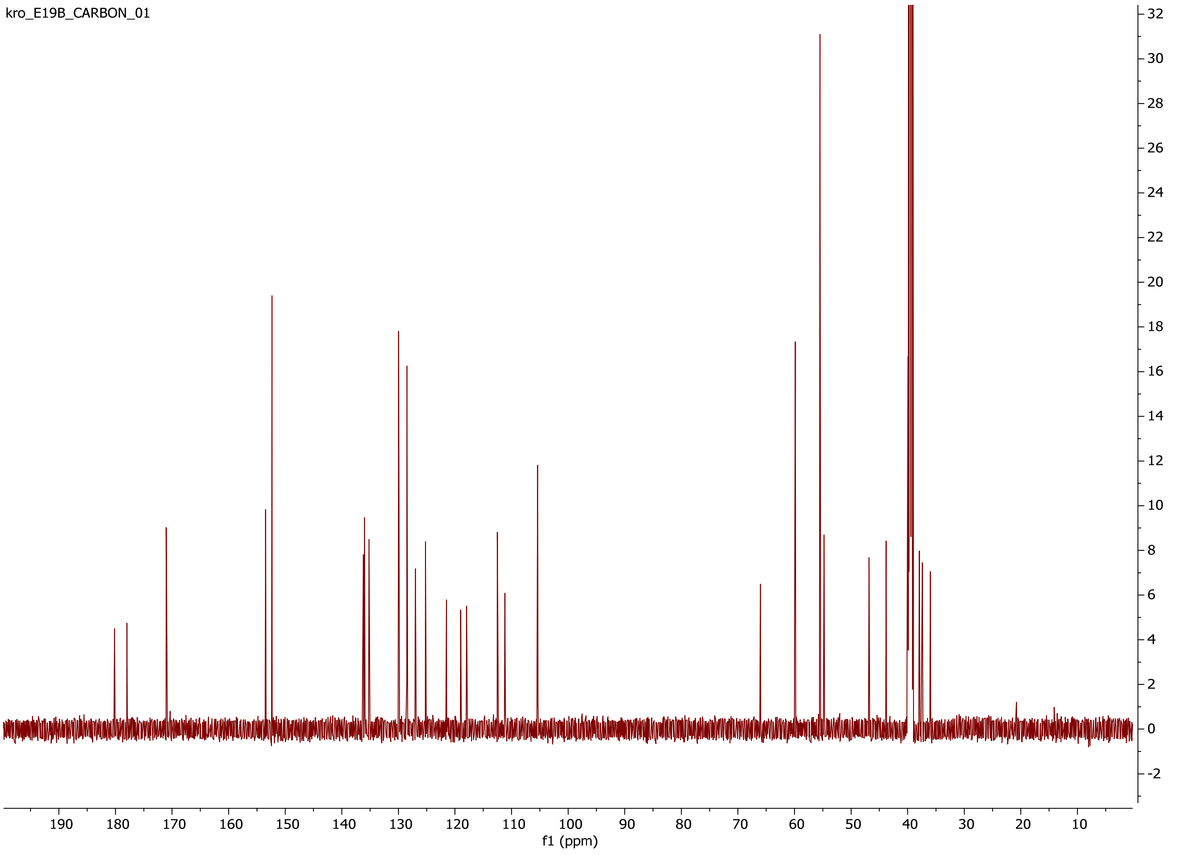

^{1}H NMR spectrum of (-)-**10a** in DMSO-d_6.

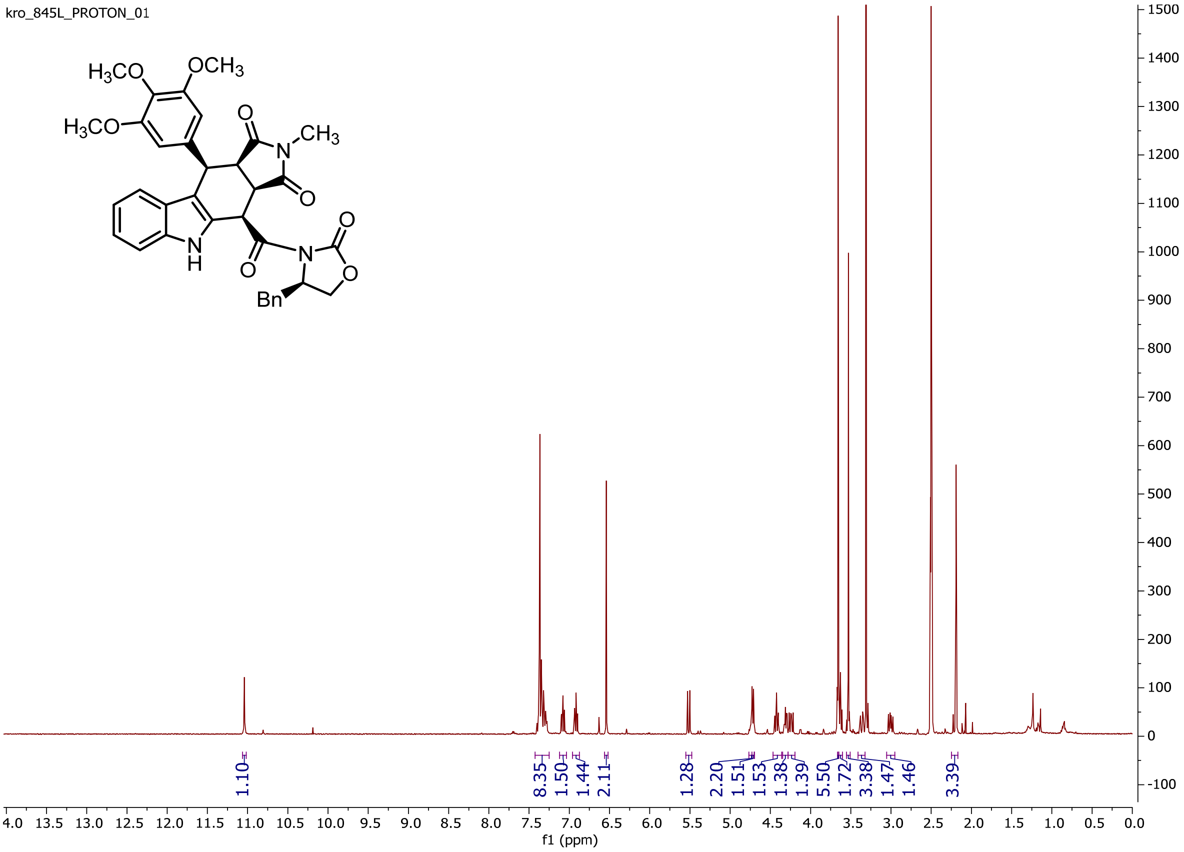

^{13}C NMR spectrum of (-)-**10a** in DMSO-d_6.

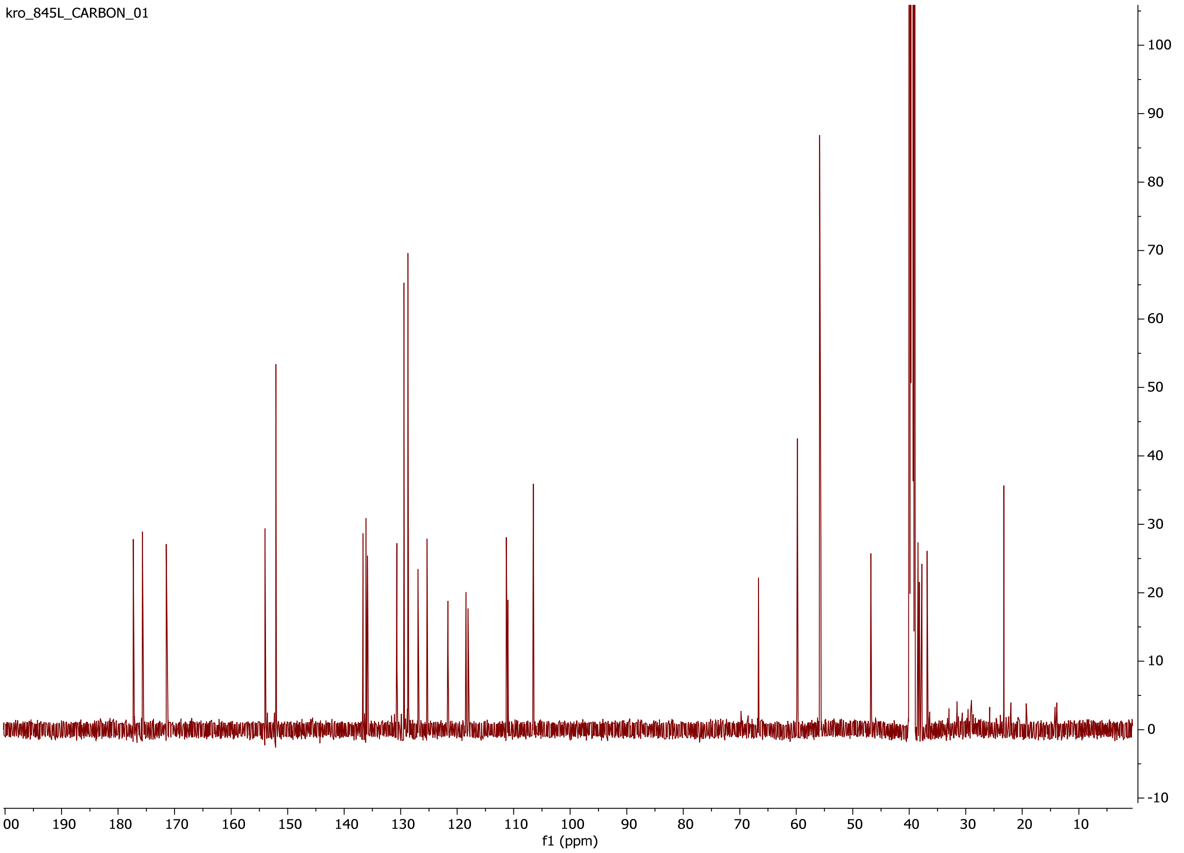

^{1}H NMR spectrum of (-)-**10b** in DMSO-d_6.

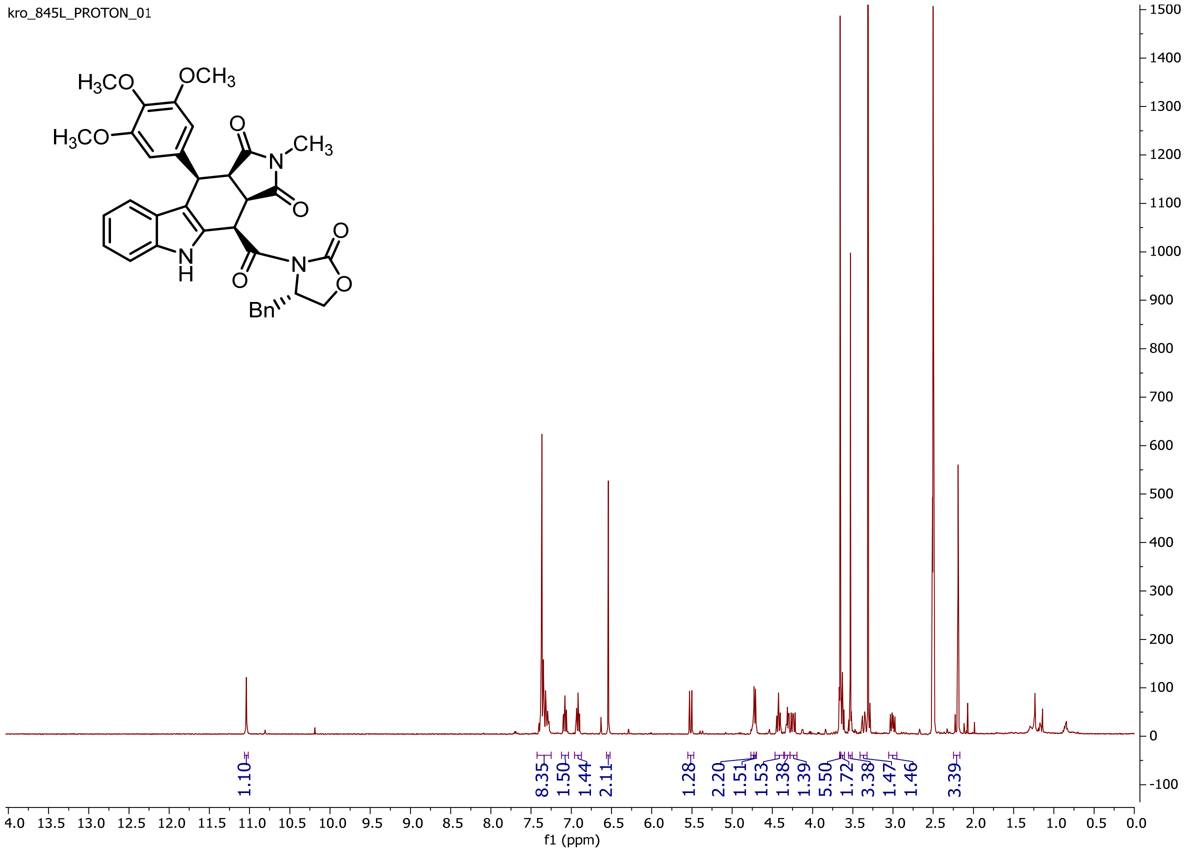

^{13}C NMR spectrum of (-)-**10b** in DMSO-d_6.

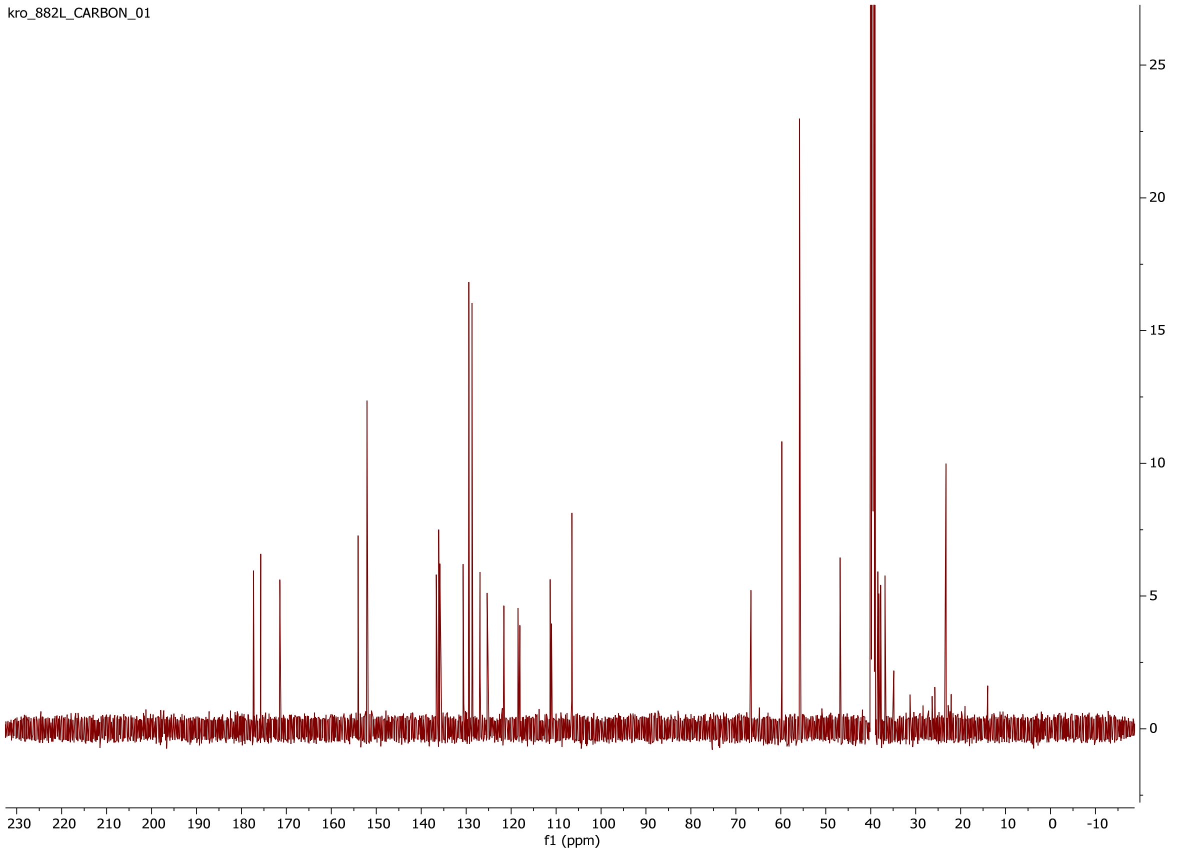

^{1}H NMR spectrum of (+)-**10a** in DMSO-*d*$_6$.

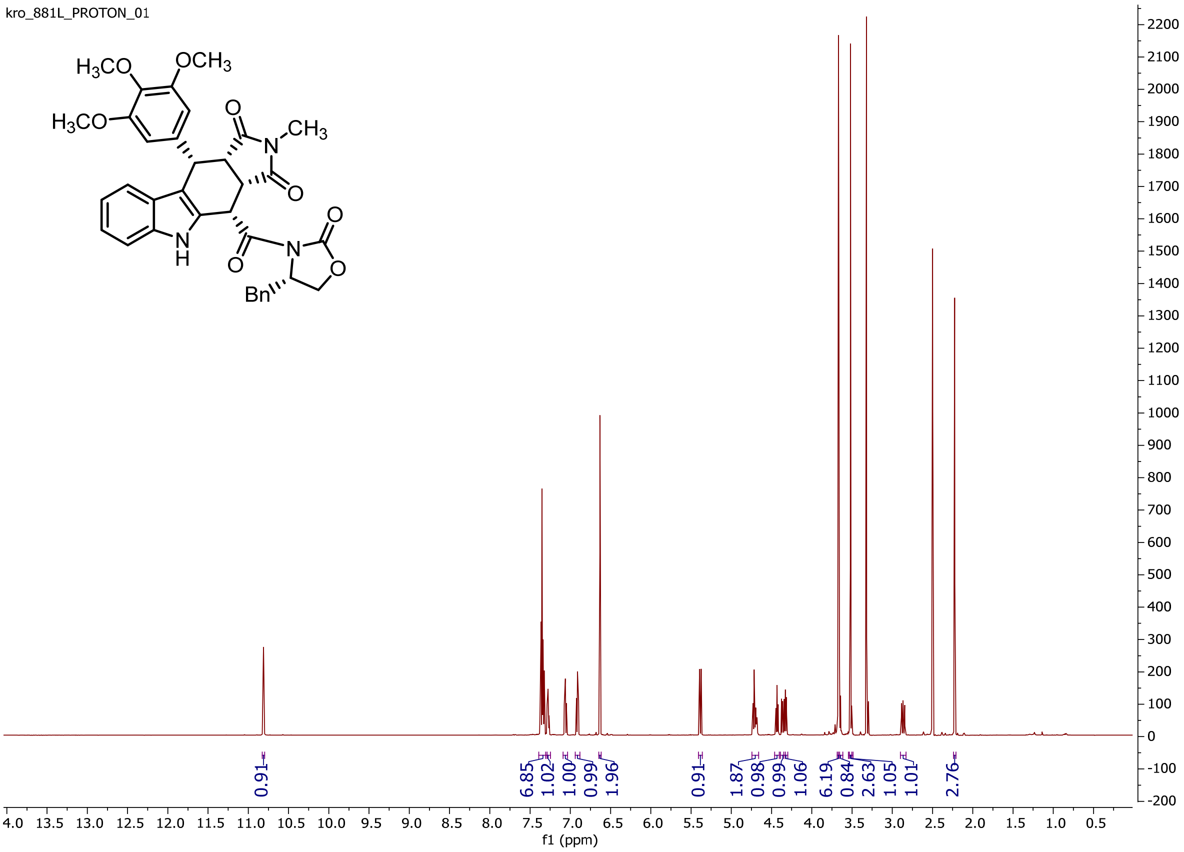

^{13}C NMR spectrum of (+)-**10a** in DMSO-*d*$_6$.

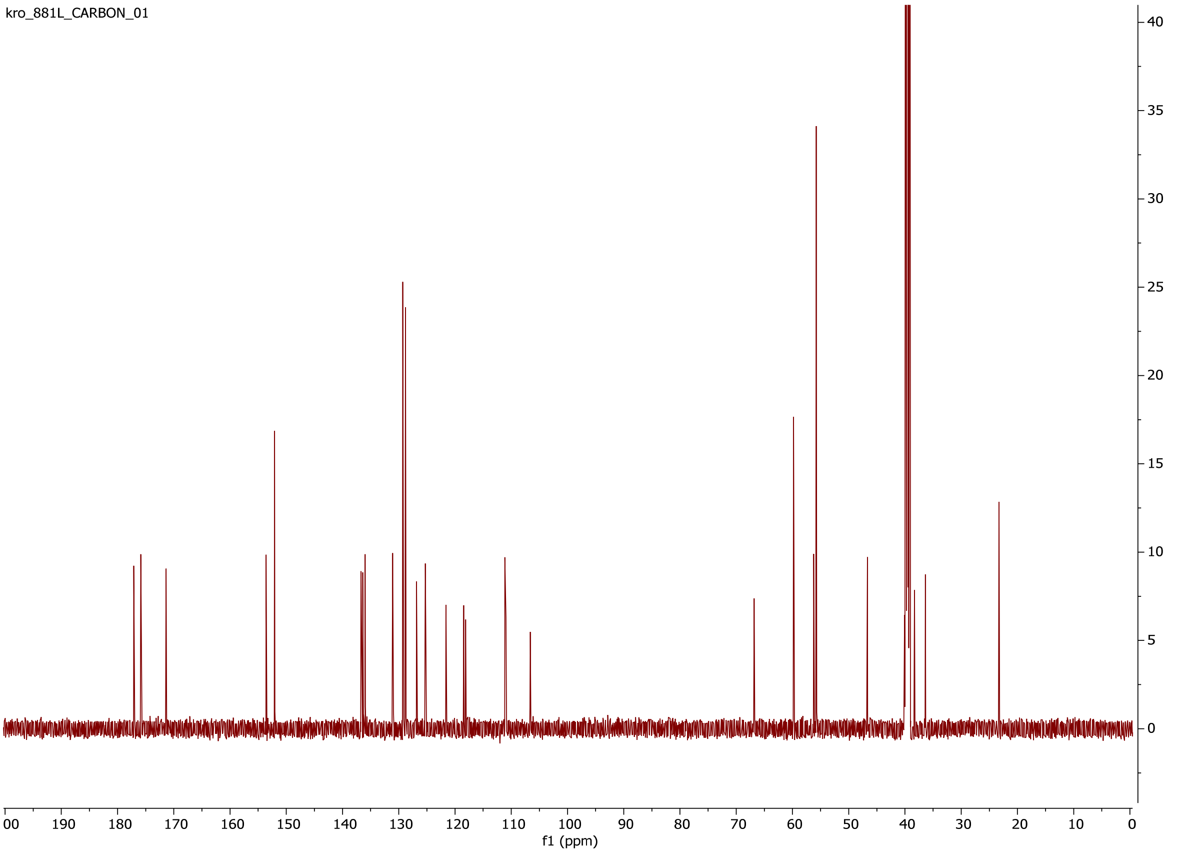

^{1}H NMR spectrum of (+)-**10b** in DMSO-*d*$_6$.

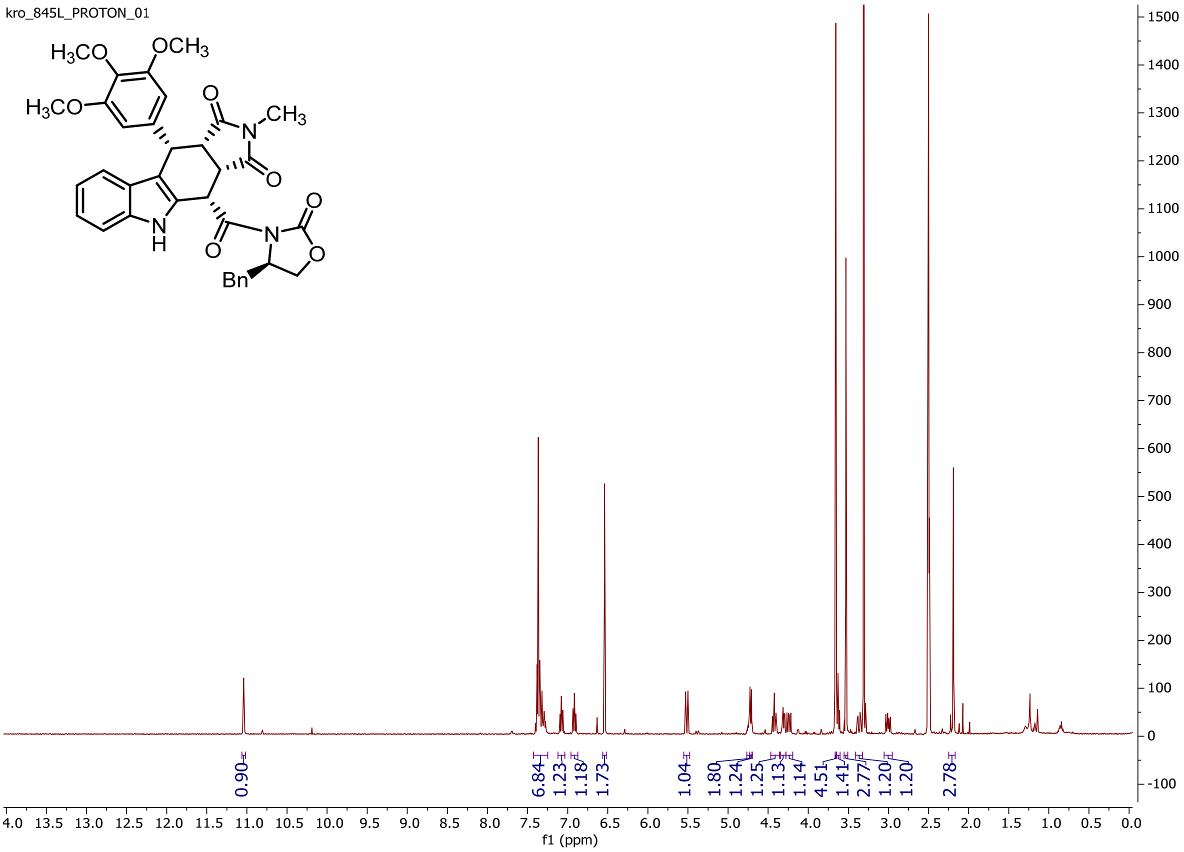

^{13}C NMR spectrum of (+)-**10b** in DMSO-*d*$_6$.

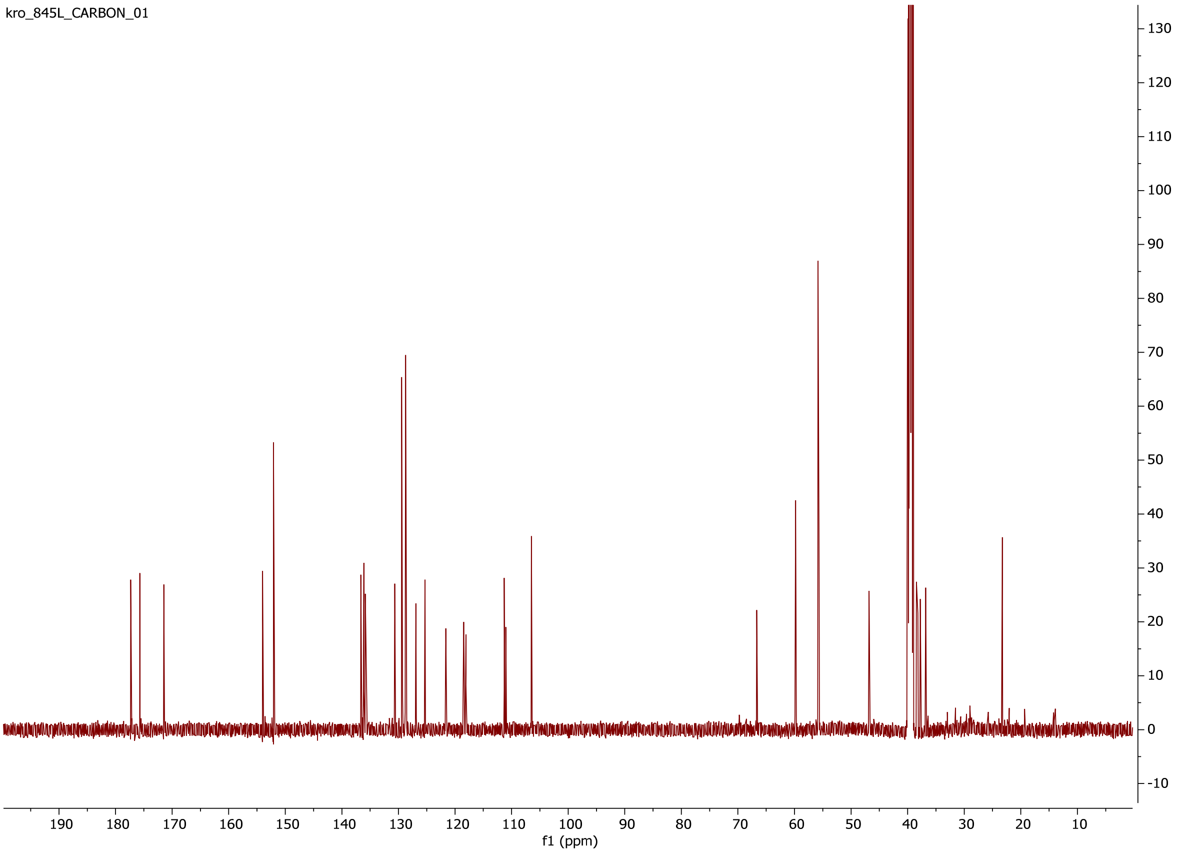

^{1}H NMR spectrum of (±)-**12** in DMSO-d_6.

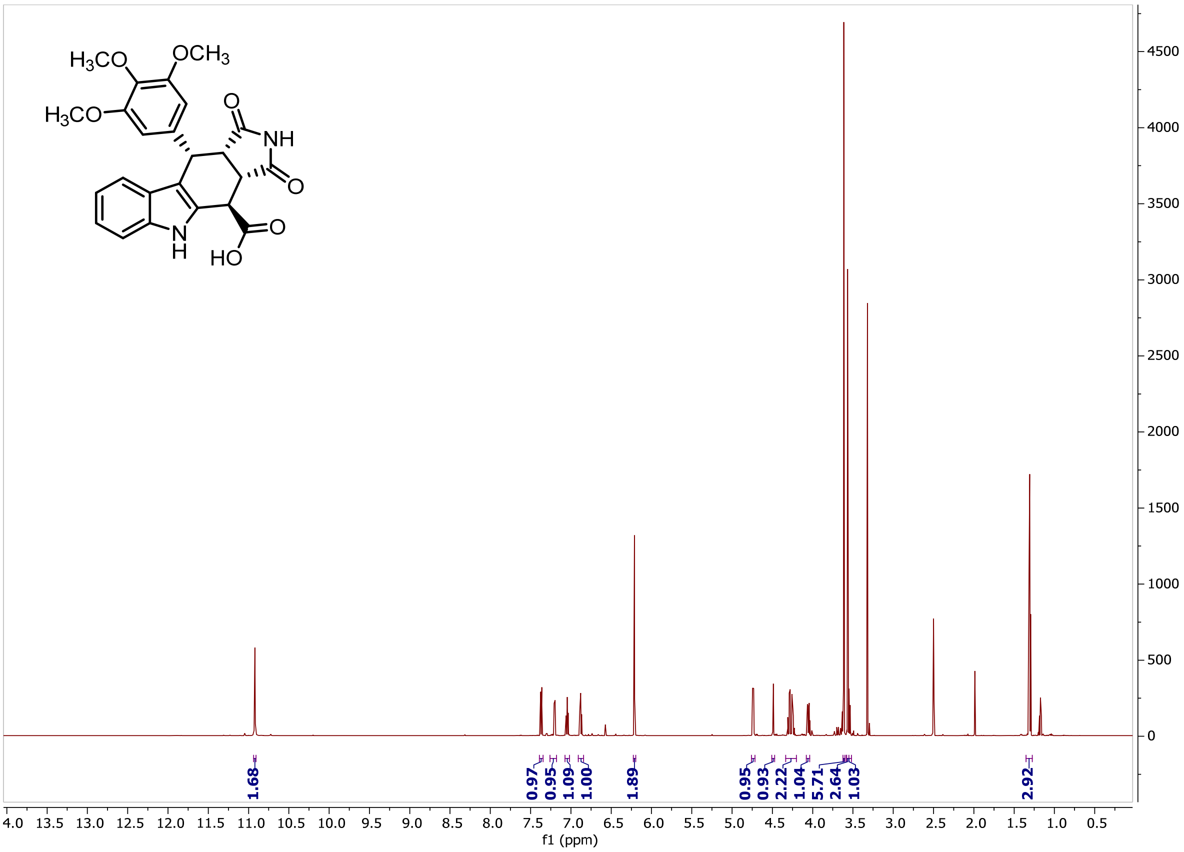

^{13}C NMR spectrum of (±)-**12** in DMSO-d_6.

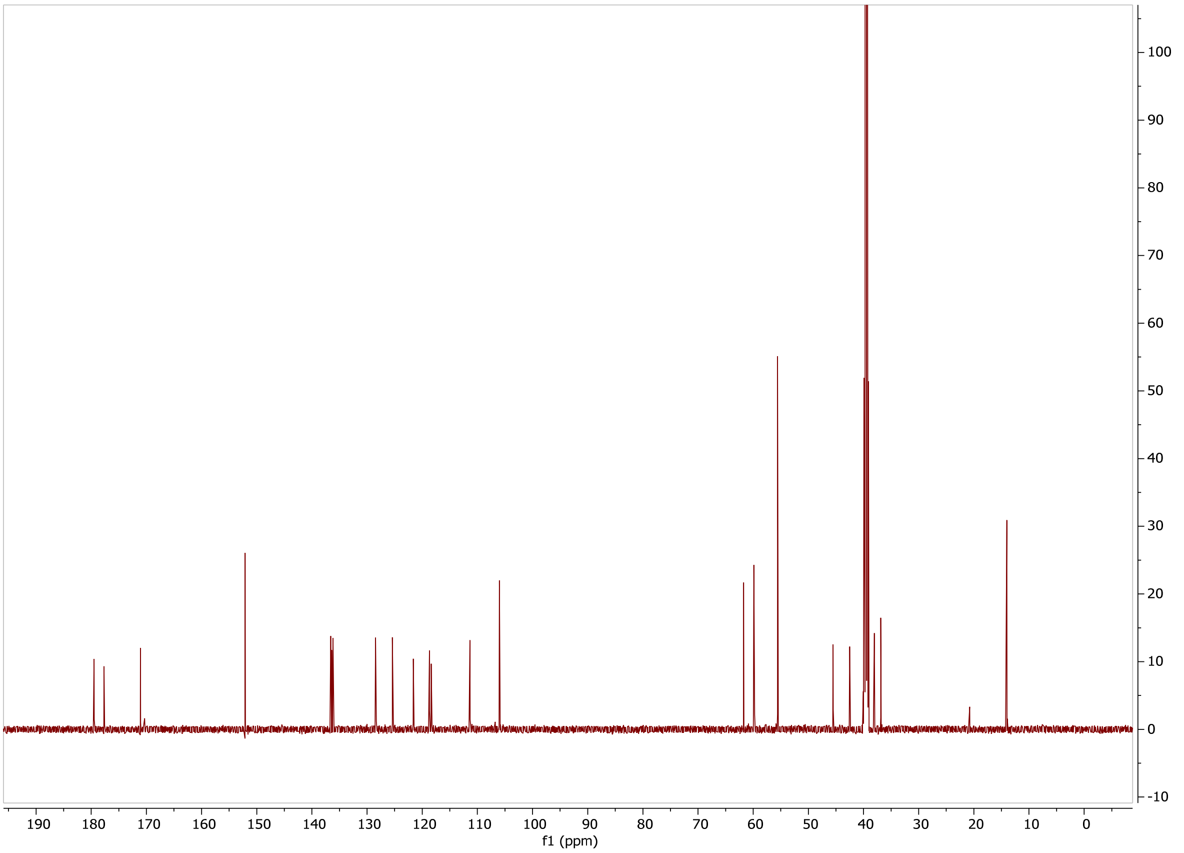

^{1}H NMR spectrum of (-)-**14c** in DMSO-d_6.

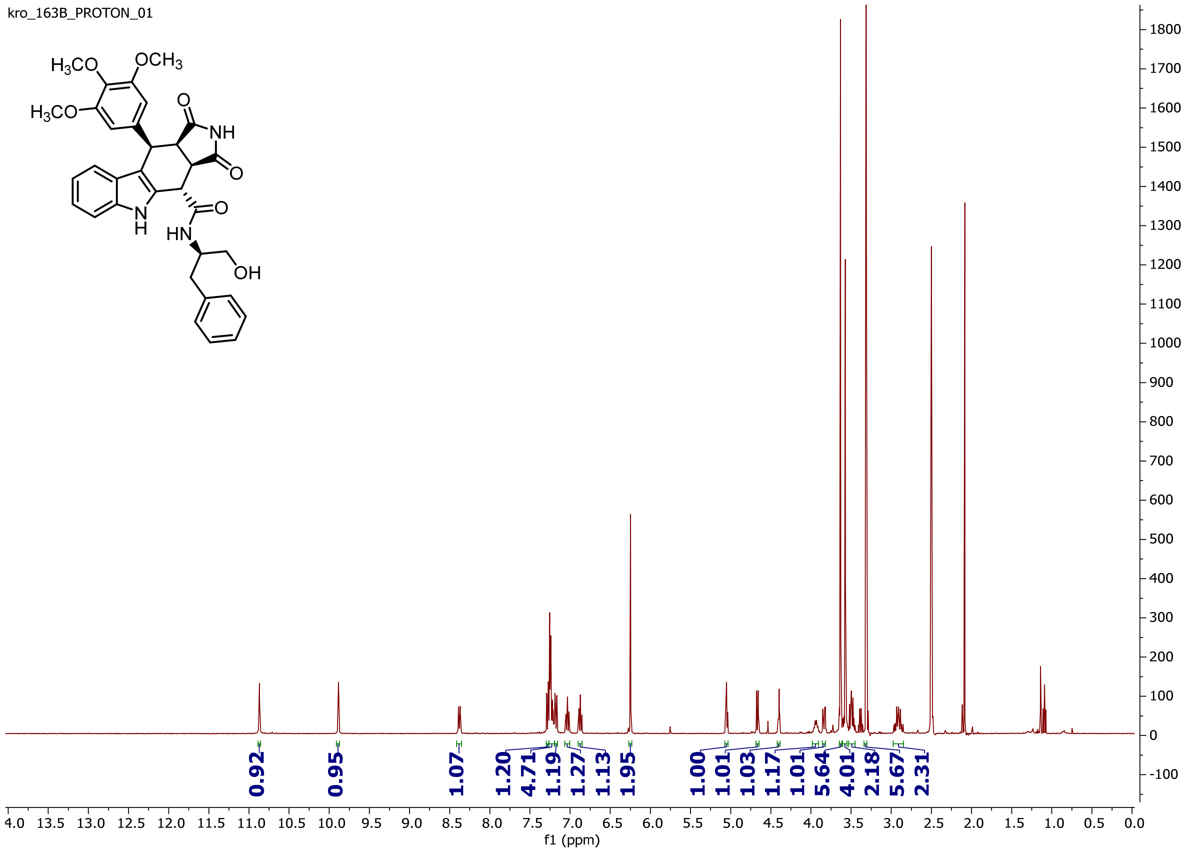

^{13}C NMR spectrum of (-)-**14c** in DMSO-d_6.

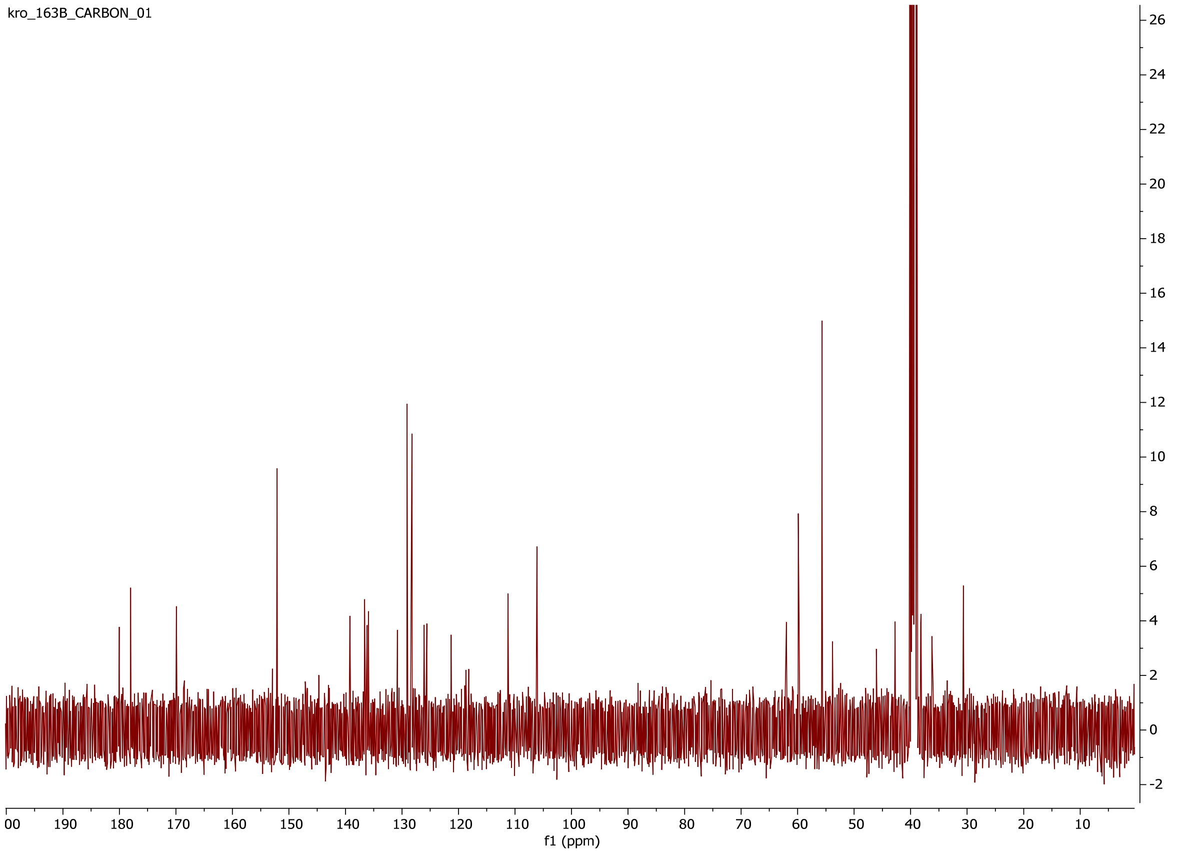

^{1}H NMR spectrum of (-)-**14d** in DMSO-*d*6.

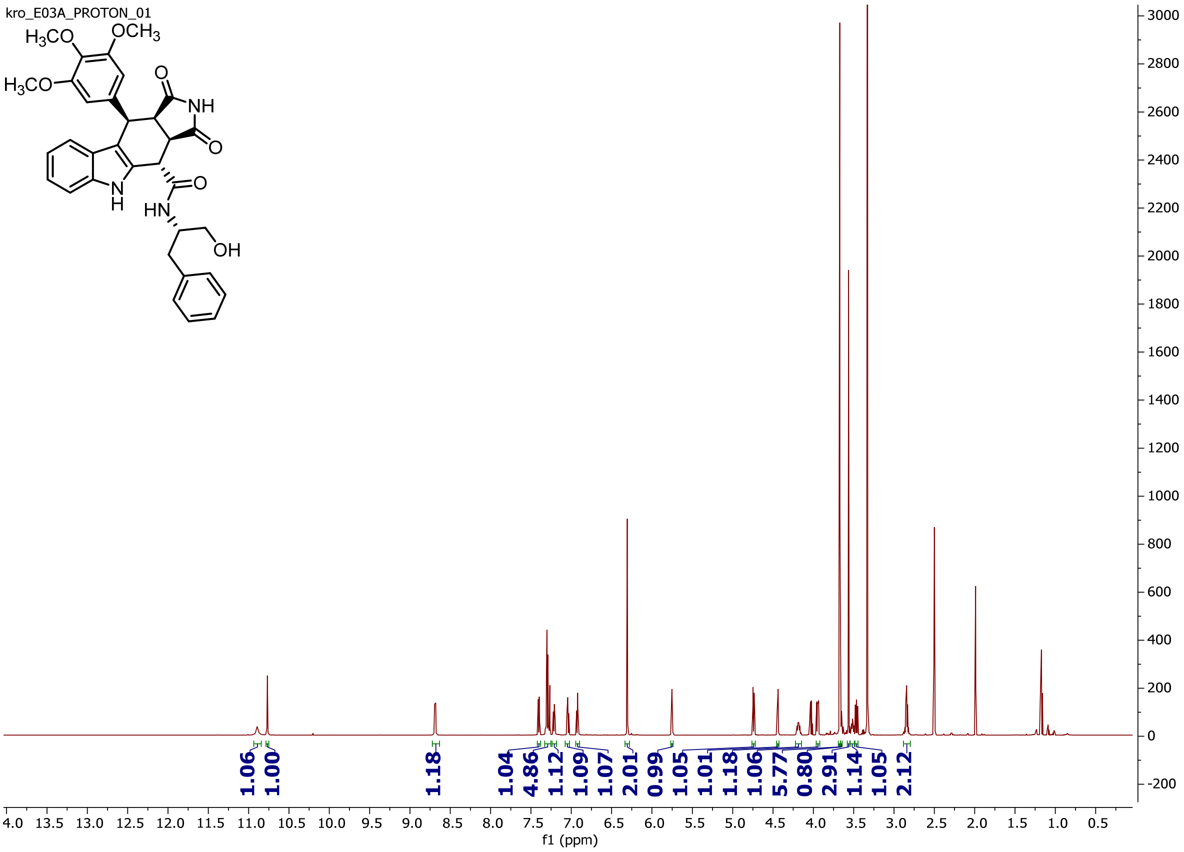

^{13}C NMR spectrum of (-)-**14d** in DMSO-*d*6.

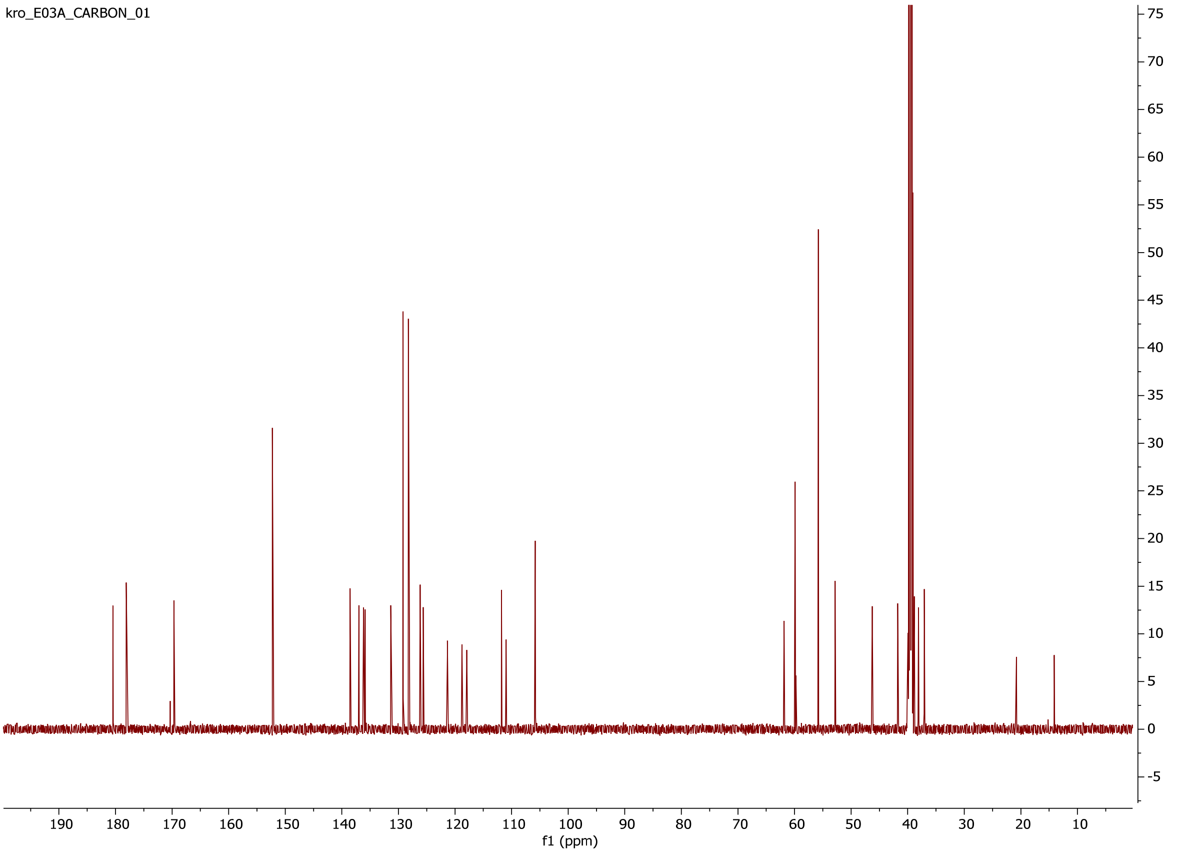

^1H NMR spectrum of (+)-**14c** in DMSO-d_6.

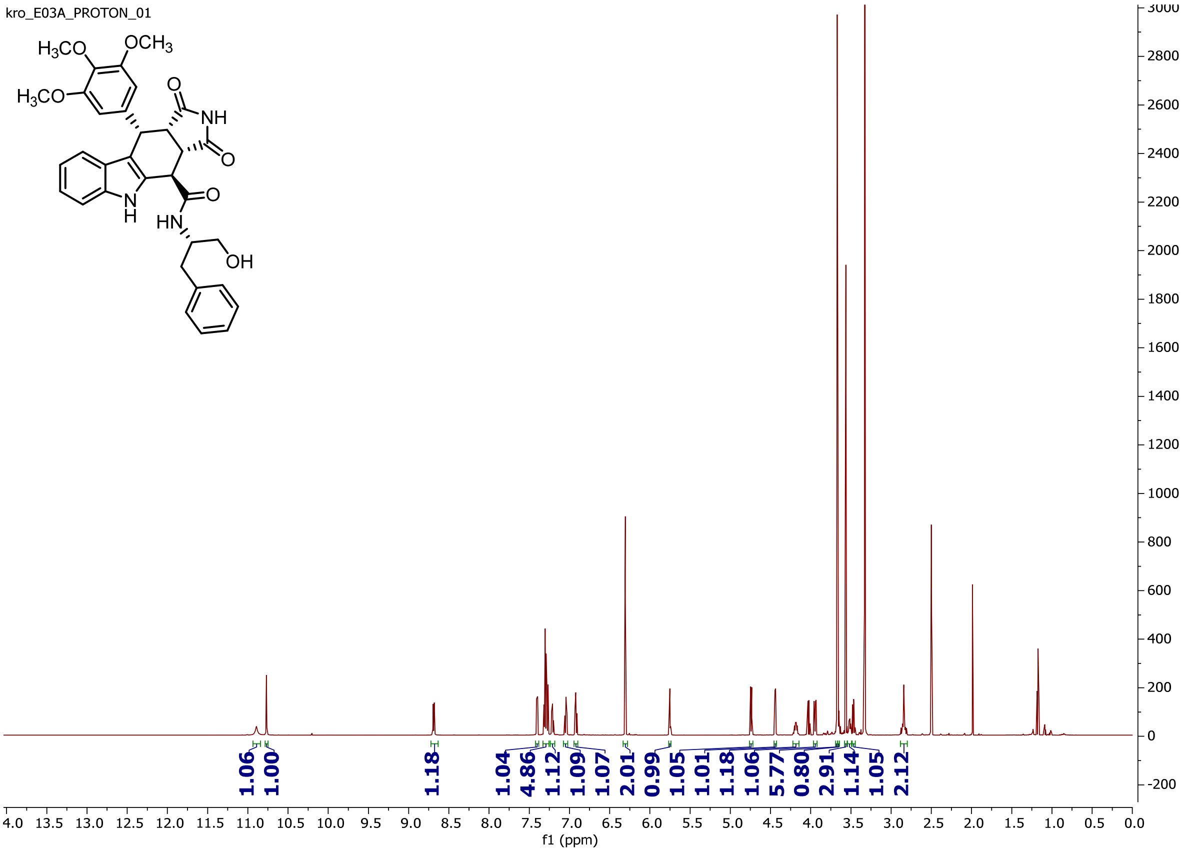

^{13}C NMR spectrum of (+)-**14c** in DMSO-d_6.

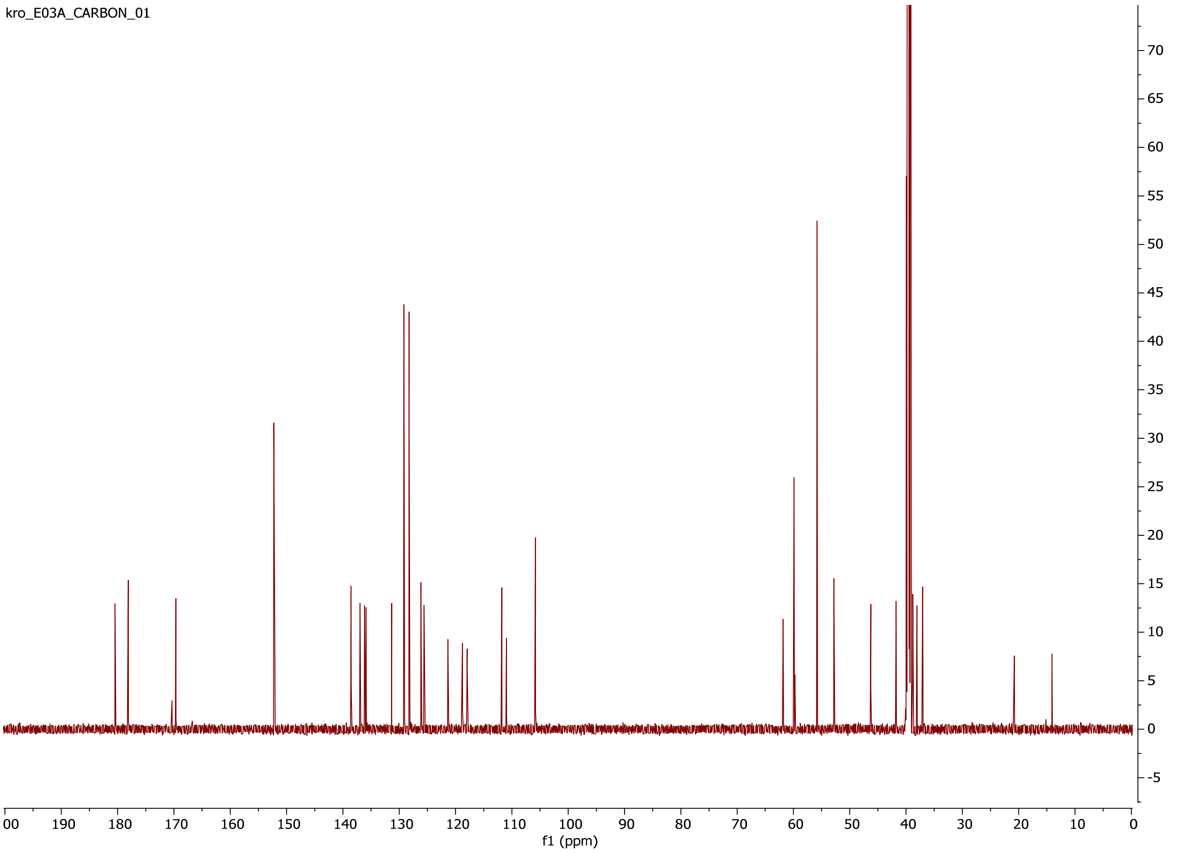

^{1}H NMR spectrum of (+)-**14d** in DMSO-d_6.

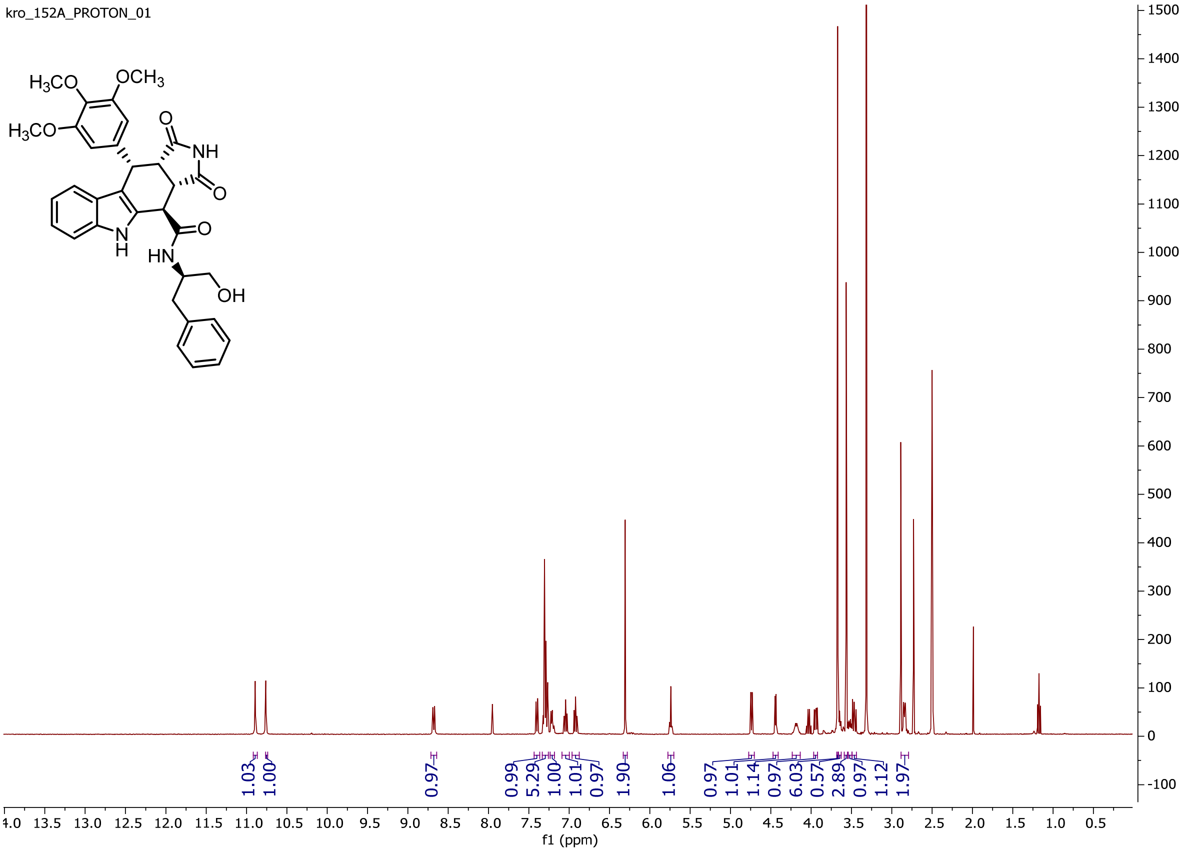

^{13}C NMR spectrum of (+)-**14d** in DMSO-d_6.

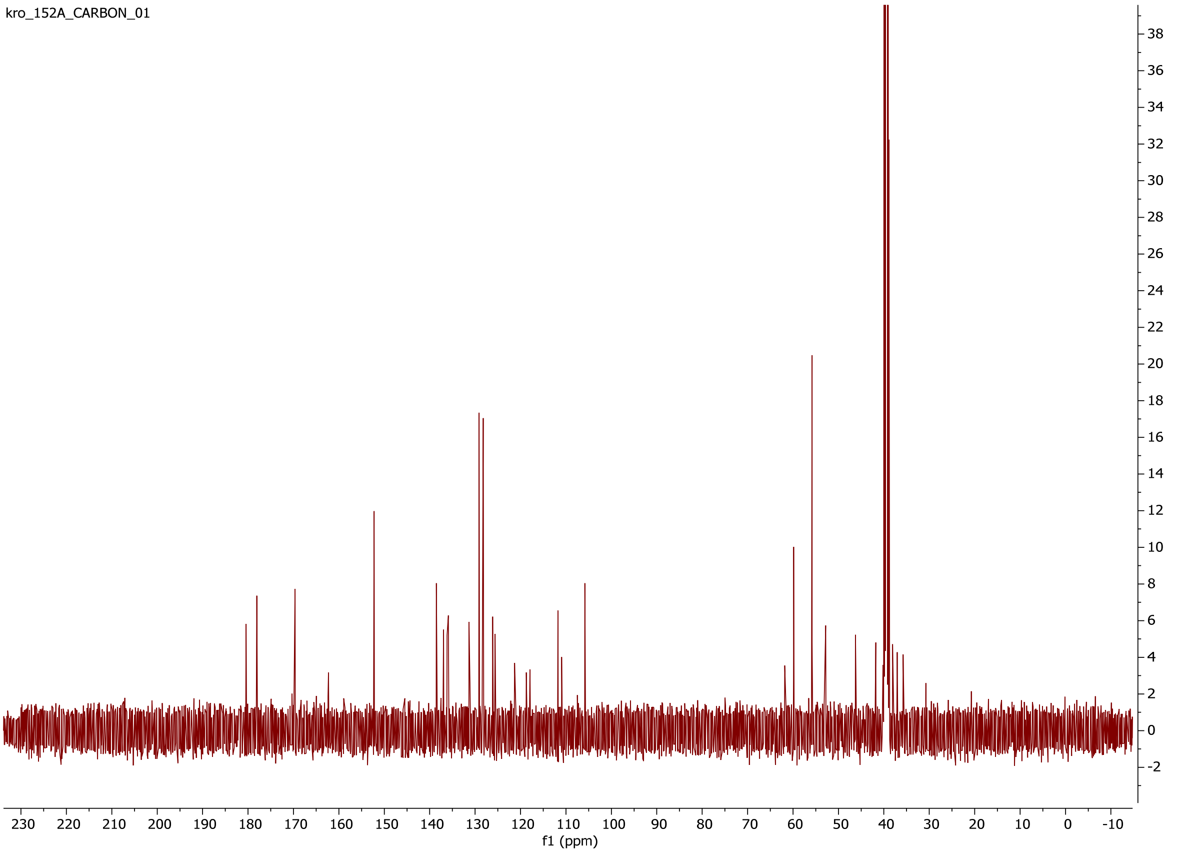

Supplement: Supplementary file 1 — Supplementary [file CMDC-15-871-s001.pdf]
